# Supplementary material for: Impact of pulse oximetry on hospital referral acceptance in children under 5 with severe pneumonia in rural Pakistan (district Jamshoro): protocol for a cluster randomised trial
Source: BMJ Open. 2021 Sep 17;11(9):e046158. doi: 10.1136/bmjopen-2020-046158 (PMC8451312; doi:10.1136/bmjopen-2020-046158)

Appendix 1: Eligibility/Hospital Referral Criteria

**Hospital Referral Criteria**	
**Intervention Group**	**Control Group**
**Severe pneumonia AND/OR Hypoxemia**<br><br>**Children of 0 – 6 days:**<br>**1.** 1. Fast Breathing (≥ 60 breaths/min)<br>**AND/OR**<br>***2. Hypoxemia (<92 SpO2 Blood Oxygen Level)***<br><br>**Children of 7 days – 59 months:**<br>**1.** Fast Breathing and/or Chest In-drawing<br>➢ 0-2months: ≥ 60 breaths/min<br>➢ 2-12months: ≥50 breaths/min<br>➢ 12-59months ≥40 breaths/min<br>**AND**<br>**2.** Any ONE General Danger Sign* and/or Stridor<br>➢ *Unable to drink/eat<br>➢ Vomiting<br>➢ Convulsions<br>➢ Lethargy/Unconsciousness<br><br>**AND/OR**<br><br>**Hypoxemia (<92 SpO2 Blood Oxygen Level)**	**Severe pneumonia**<br><br>**Children of 0 – 6 days:**<br>**1.** Fast Breathing (≥ 60 breaths/min)<br><br>**Children of 7 days – 59 months:**<br>**1.** Fast Breathing and/or Chest In-drawing<br>➢ 0-2months: > 60 breaths/min<br>➢ 2-12months: ≥50 breaths/min<br>➢ 12-59months ≥40 breaths/min<br>AND<br>**2.** Any ONE General Danger Sign* and/or Stridor<br>➢ *Unable to drink/eat<br>➢ Vomiting<br>➢ Convulsions<br>➢ Lethargy/Unconsciousness

Appendix 2: Informed Consent

Research Consent Form

Title of Research Project:

Impact of Pulse Oximetry on Hospital Referral Acceptance in children under 5 with severe pneumonia in rural Pakistan (District Jamshoro): a cluster randomized trial (GAPPD Scale up Project)

Investigators:

Dr. Fatima Mir (Principal investigator PO Project, AKU)

Department of Pediatrics and Child Health, Aga Khan University, Karachi, Pakistan

Tel: 92-21-34864955

Dr. Sajid Soofi (PI, Scale up of GAPPD in Pakistan)

Centre of Excellence in Women & Child Health, The Aga Khan University, Pakistan.

Tel: 92-21-34864955

Professor Zulfiqar Bhutta (Senior Investigator, Scale up of GAPPD in Pakistan)

Centre of Excellence in Women & Child Health, The Aga Khan University, Pakistan.

Tel: 92-21-34864955

Purpose of the Research:

Severe Pneumonia is a serious and possibly life-threatening infection in young children aged under 5 years. It may involve fast breathing, chest indrawing, and danger signs like inability to feed, convulsions, persistent vomiting and decreased movement. Hypoxemia is now recognized as an additional sign of severe pneumonia needing hospital admission. Pulse oximetry (the 'oxygen test') is a simple method whereby lady health workers can check level of oxygen in blood of children and decide which young babies need immediate medical care and referral to hospital for antibiotics and oxygen and which babies can be treated in the health center itself.

In this study, we are testing whether use of pulse oximetry in addition to other danger signs of severe pneumonia will influence families to accept hospital referral in comparison to areas where danger signs other than hypoxemia are used.

This project is a collaboration between Aga Khan University (AKU) in Karachi and the Gates Foundation, Seattle, USA.

Description of the Research:

We are inviting children aged 0 to 59 months screened at home to join this research study. If your baby joins the study, the following activities will take place:

1) **Questionnaire**. We will ask several questions about the health of your baby, as well as check him/her for presence of pneumonia. If he/she has signs of severe pneumonia (with or without hypoxemia), he will be eligible to take part in our study.

2) **Pulse oximetry**. Next your child may or may not undergo a pulse oximetry measurement dependent on whether he resides in an intervention or non-intervention cluster. The process will be painless for the child and will involve placing a sensor on your baby's foot or hand. Each sensor is attached to a pulse oximetry machine. A number on the screen of the machine shows the oxygen level. A level including and above 92% will be regarded as normal.

   All babies with fast breathing or chest in drawing pneumonia AND any one of general danger signs (inability to feed, persistent vomiting, decreased movement, convulsions) with or without hypoxemia (SaO2 <92%) will be advised and helped to go to an assigned hospital for treatment (antibiotics and possibly oxygen supportive therapy). Their clinical status over there will be followed and transfer to hospital and subsequent treatment will be facilitated by the study.

   All responses will be documented on paper forms. We will arrange for quick referral to hospital if your baby has low oxygen level or other signs of serious illness. Follow-up visits by our study staff will be conducted to check the status of your baby.

3) You may be asked detailed questions based on whether you accept or refuse hospital referral to allow us to understand what factors contributed to your decision making. Since this process will take time, we will do it after the child's treatment plan has been started at hospital or at home.

4) **Video recording**. Some infants will be video recorded during the study. The researchers will look at these videos to make quality checks on study conduct and procedures. You can refuse video recording but still join the rest of the study. If you provide permission, some videos may be shown publicly for education purposes, but your child's name will be kept private. The videos or images from them will not be shown or sold for financial profit.

5) **Stored information**. This study is connected to other studies coordinated by the Aga Khan University. If you agree to join this study, we will access information about your baby collected as part of the Aga Khan University demographic surveillance system and other Aga Khan University studies to which you have already allowed, or will allow, your baby to join.

We expect to enrol about 4160 children with severe pneumonia in this study.

Potential Harms, Discomforts, or Inconveniences:

There are no harms or discomforts that could be caused to your baby by taking part in this study.

Potential Benefits to individual participants:

Your baby will receive free treatment at the hospital if referral is accepted. The baby will also receive two follow up visits to check if he/she is recovering as expected on day 7 and 14 as part of the study. If you refuse to accept hospital referral for injectable antibiotics and oxygen, we will provide less ideal oral treatment options at home.

Potential Benefits to Society:

We may learn more about the usefulness of pulse oximetry for assessment of young babies in your community and other similar places.

Confidentiality:

We will respect your privacy. No information about you or your child will be given to anyone or be published without your permission, unless required by law. The paper forms, videos and electronic information made in this study will be stored in a secure, locked location. Only members of the research team will have access to them. BMGF (the sponsor) or AKU Clinical Research Monitors may look at your child's records to check on the study. By signing this consent form, you agree to let these people look at your child's records. We will put a copy of this research consent form in your child's patient health record and give you a copy as well. After the study has been completed, the forms and videos will be kept as long as required by BMGF and AKU policies. They will then be destroyed according to these same policies. Paper and electronic forms from this study will be stored for at least 7 years after publication of the study. Published study results will not reveal your identity or the identity of your baby.

Payment

No payment will be given for participation in this study.

Participation:

You decide if your child joins this study. If you choose to let your child take part, you can take your child out of the study at any time.

In the unlikely situation that your child becomes ill or is harmed because of study participation, we will treat your child for free. Your signing this consent form does not interfere with your legal rights in any way. The staff of the study, any people who gave money for the study, or the hospital are still responsible, legally and professionally, for what they do during this study.

Sponsorship:

The funder of this research is the Bill and Melinda Gates Foundation Seatttle, USA.

Conflicts of interest:

None of the researchers have any conflicts of interest to declare.

Consent:

By signing this form, you agree that:

1) The study has been explained to you.
2) All of your questions have been answered.
3) The possible harms and benefits of this study have been explained to you.
4) You may ask questions about the study now and in the future.
5) You have been told that your child's medical records will be kept private except as described to you.
6) You understand that information about your child will not be given to anyone or be published without first asking your permission.

I agree that my child____ may take part in this study.

I understand that videos may be used to assess the success of pulse oximetry. I understand that I may refuse to permit video-recording of my child, yet take part in other aspects of the study. I understand that even if I permit video-recording now, I may refuse the use of these videos at a later time. I understand that I may permit video-recording of my child for researchers to view, but refuse any public display of the videos.

I agree to permit my child and me to be video-recorded during this study.

☐ YES ☐ NO

I agree to permit whole or parts of videos of my child and me to be shown publicly for educational purposes.

☐ YES ☐ NO

________________________________ ________________________________

Printed Name of Parent/Legal Guardian Parent/Legal Guardian's signature/Thumb print & date

________________________________ ________________________________

Printed Name of person who explained consent Signature of Person who explained consent & date

____ ________________________________

Printed Witness' name Witness' signature & date

(If the parent/legal guardian does not read Sindhi)

If you have any questions about this study, please call Dr Fatima Mir at _________________

If you have questions about your child's rights as a subject in a study or injuries during a study, please call Coordinator, Bioethics Unit at __________________

Appendix 3: Study Activities

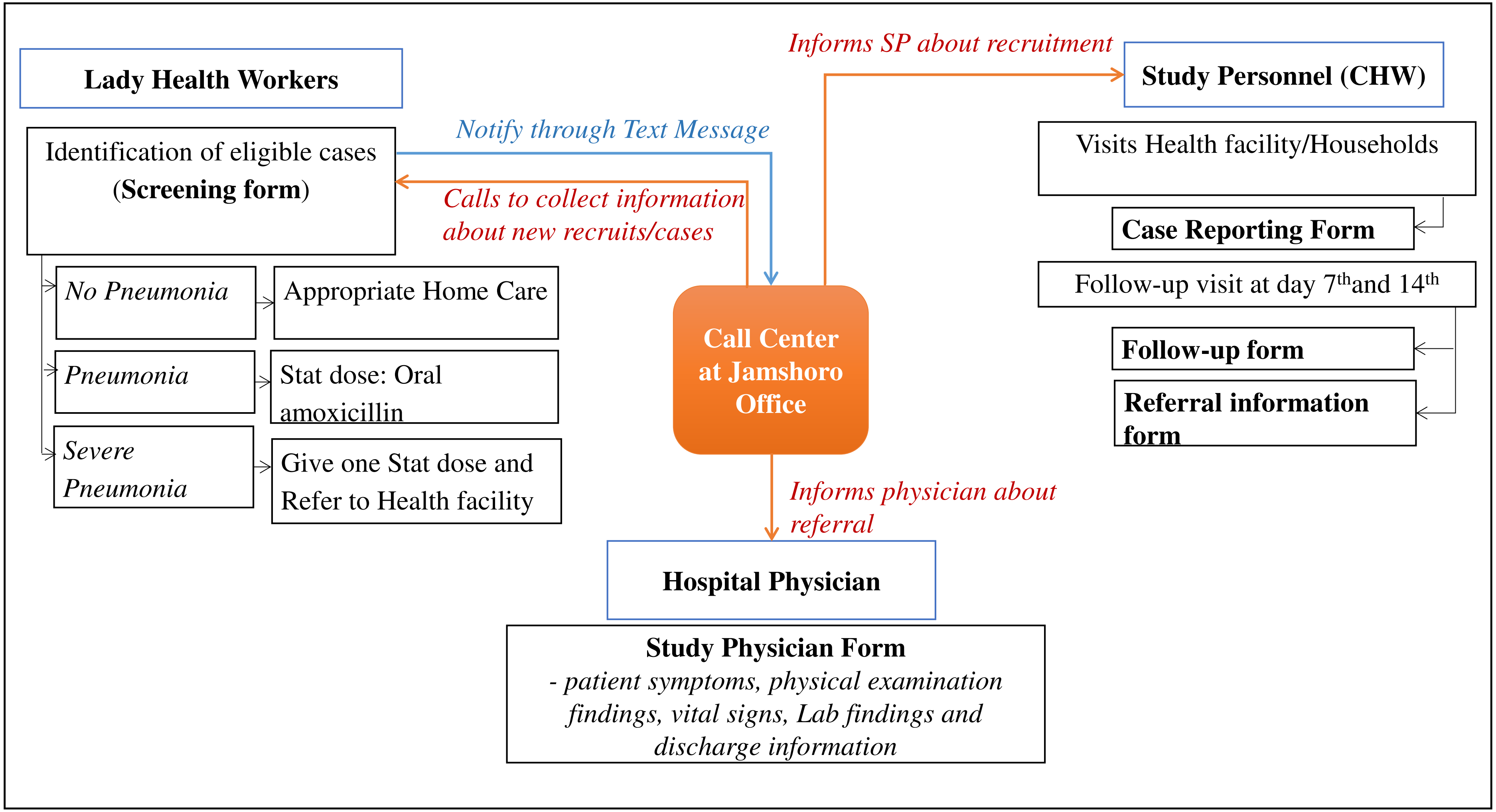

Supplement: Supplementary data [file bmjopen-2020-046158supp001.pdf]
