# Supplementary material for: Impact of pulse oximetry on hospital referral acceptance in children under 5 with severe pneumonia in rural Pakistan (district Jamshoro): protocol for a cluster randomised trial
Source: BMJ Open. 2021 Sep 17;11(9):e046158. doi: 10.1136/bmjopen-2020-046158 (PMC8451312; doi:10.1136/bmjopen-2020-046158)
Supplement: Author's manuscript [file bmjopen-2020-046158.draft_revisions.pdf]

BMJ Open is committed to open peer review. As part of this commitment we make the peer review history of every article we publish publicly available.

When an article is published we post the peer reviewers' comments and the authors' responses online. We also post the versions of the paper that were used during peer review. These are the versions that the peer review comments apply to.

The versions of the paper that follow are the versions that were submitted during the peer review process. They are not the versions of record or the final published versions. They should not be cited or distributed as the published version of this manuscript.

BMJ Open is an open access journal and the full, final, typeset and author-corrected version of record of the manuscript is available on our site with no access controls, subscription charges or pay-per-view fees (http://bmjopen.bmj.com).

If you have any questions on BMJ Open's open peer review process please email info.bmjopen@bmj.com

BMJ Open

Impact of Pulse Oximetry on Hospital Referral Acceptance in Children under 5 with Severe Pneumonia in Rural Pakistan (District Jamshoro): Protocol for a Cluster Randomized Trial

Journal:	*BMJ Open*
Manuscript ID	bmjopen-2020-046158
Article Type:	Protocol
Date Submitted by the Author:	22-Oct-2020

Complete List of Authors:	Mir, Fatima; Aga Khan University, Pediatrics and Child Health; Aga Khan University, Aga Khan University<br>Ali Nathwani, Apsara; Aga Khan University,<br>chanar, suhail; Aga Khan University, Paediatrics and child health<br>Hussain, Amjad; Aga Khan University, Pediatrics and Child Health<br>Rizvi, Arjumand; Aga Khan Medical University, Pediatrics and Child Health<br>Ahmed, Imran; Aga Khan University<br>Memon, Zahid Ali; Aga Khan University, Centre of Excellence in Women and Child Health<br>Habib, Atif; Aga Khan University, Department of Paediatrics & Child Health<br>soofi, sajid; Aga Khan University Hospital, Paediatric & Child Health<br>Bhutta, Zulfiqar; Aga Khan University, Division of Women and Child Health

Keywords:	Public health < INFECTIOUS DISEASES, Paediatric infectious disease & immunisation < PAEDIATRICS, PUBLIC HEALTH, RESPIRATORY MEDICINE (see Thoracic Medicine), Clinical trials < THERAPEUTICS

SCHOLARONE™
Manuscripts

I, the Submitting Author has the right to grant and does grant on behalf of all authors of the Work (as defined in the below author licence), an exclusive licence and/or a non-exclusive licence for contributions from authors who are: i) UK Crown employees; ii) where BMJ has agreed a CC-BY licence shall apply, and/or iii) in accordance with the terms applicable for US Federal Government officers or employees acting as part of their official duties; on a worldwide, perpetual, irrevocable, royalty-free basis to BMJ Publishing Group Ltd ("BMJ") its licensees and where the relevant Journal is co-owned by BMJ to the co-owners of the Journal, to publish the Work in this journal and any other BMJ products and to exploit all rights, as set out in our licence.

The Submitting Author accepts and understands that any supply made under these terms is made by BMJ to the Submitting Author unless you are acting as an employee on behalf of your employer or a postgraduate student of an affiliated institution which is paying any applicable article publishing charge ("APC") for Open Access articles. Where the Submitting Author wishes to make the Work available on an Open Access basis (and intends to pay the relevant APC), the terms of reuse of such Open Access shall be governed by a Creative Commons licence – details of these licences and which Creative Commons licence will apply to this Work are set out in our licence referred to above.

Other than as permitted in any relevant BMJ Author's Self Archiving Policies, I confirm this Work has not been accepted for publication elsewhere, is not being considered for publication elsewhere and does not duplicate material already published. I confirm all authors consent to publication of this Work and authorise the granting of this licence.

For peer review only

Impact of Pulse Oximetry on Hospital Referral Acceptance in Children under 5 with Severe Pneumonia in Rural Pakistan (District Jamshoro): Protocol for a Cluster Randomized Trial

Fatima Mir[1]*, Apsara Ali Nathwani[2], Suhail Chanar[2], Amjad Hussain[2], Arjumand Rizvi[2], Imran Ahmed[2], Zahid Memon[2], Muhammad Atif Habib[2], Sajid Soofi[1,2], Zulfiqar A Bhutta[2]

Affiliations

[1]Department of Pediatrics and Child Health, The Aga Khan University, Pakistan

[2]Centre of Excellence in Women & Child Health, The Aga Khan University, Pakistan.

***Corresponding author**

Dr. Fatima Mir

Associate Professor

Department of Pediatrics and Child Health, the Aga Khan University

Stadium Road, Karachi 74800

Pakistan

Email: fatima.mir@aku.edu

Phone: +92 3005844610

Key words: Severe pneumonia, hypoxemia, pulse-oximetry, Lady Health Workers (LHWs), community case management pneumonia, hospital referral.

For peer review only

Abstract

Background: Pneumonia is a leading cause of death among children under five specifically in South Asia and Sub-Saharan Africa. Hypoxemia is a life-threatening complication among children under five with pneumonia. Hypoxemia increases risk of mortality by 4.3 times in children with pneumonia than those without hypoxemia. Prevalence of hypoxemia varies with geography, altitude and severity (9-39% Asia, 3-10% African countries). In this protocol paper we describe research methods for assessing impact of lady health workers identifying hypoxemia in children with signs of pneumonia during household visits on acceptance of hospital referral in District Jamshoro, Sindh.

Methods and Analysis: A cluster randomized controlled trial using pulse oximetry as intervention for children with severe pneumonia will be conducted in community settings. Children aged 0-59 months with signs of severe pneumonia will be recruited by Lady Health Workers (LHWs) during routine visits in both intervention and control arms after consent. Severe pneumonia will be defined as fast breathing and/or chest in-drawing, and, one or more danger sign and/or hypoxemia (Sa02<92%) in PO (intervention) group and fast breathing and/or chest in-drawing and 1/more danger sign in clinical signs (control) group. Recruits in both groups will receive a stat dose of oral amoxicillin and referral to designated tertiary health facility. Analysis of variance will be used to compare baseline referral acceptance in both groups with that at end of study.

Ethics and Dissemination: Ethical approval was granted by the Ethics Review Committee of the Aga Khan University (4722-Ped-ERC-17), Karachi. Study results will be shared with relevant government and non-governmental organizations, presented at national and international research conferences and published in international peer-reviewed scientific journals.

Trial registration: Clinicaltrials.gov Registry NCT03588377. Trial registration Date: May 05, 2018. Last update Date: July 31 2020. Study Status: ongoing

For peer review only - http://bmjopen.bmj.com/site/about/guidelines.xhtml

For peer review only

Article Summary

Strengths and Limitations of this study

- Our study will directly assess impact of pulse oximetry on family referral acceptance by comparing family acceptance of hospital referral (recommended component of case management for severe pneumonia) in ‘pulse oximetry and clinical signs’ group with ‘clinical signs alone’ group
- The study has applicability because it assesses impact with ‘real-life’ limitations (variability in lady health worker accuracy in identifying severe pneumonia, availability of oxygen and human resource at referral hospital and, availability of private transportation)
- The study design provides means of minimizing the effect of confounding
- The study design avoids bias in allocation to exposure groups
- Blinding is not possible due to nature of intervention
- Some subjects may fail to adhere to protocol and non-adherence may cause an underestimated measure of association.

For peer review only - http://bmjopen.bmj.com/site/about/guidelines.xhtml

Introduction

Pneumonia accounts for an estimated 18% of under-5 mortality across the globe [1] Majority of these pneumonia-specific deaths occur in 15 countries, in which Pakistan ranks fifth. [2] Failure to seek early care and delays in hospital referral are commonly acknowledged determinants of mortality in childhood pneumonia with a higher proportion reported from rural settings than urban. [3-6] Acceptance rates of 'facilitated' hospital referral advice have been reported low between 8% and 23% for sick young infants in peri-urban Karachi. [7, 8] They are even lower for non-facilitated referral in rural settings in children under 5 with severe pneumonia in rural Matiari district, Sindh. [5] The prominent reasons in developing countries behind this delay are inability to recognize seriousness of pneumonia, distance from health facility and lack of money for private health care. [9, 10]

In 1994, the Government of Pakistan introduced the Lady Health Worker (LHW) Program in rural populations with low physician density to address common health problems in women and children under 5 through household visits. To date, a team of over 110,000 LHWs are working for the program nationwide with 23,185 LHWs in Sindh alone [11]. Each LHW is responsible for a population of 1000–1500 individuals (catchment of about 100 families). Recruits are preferably local, with a minimum of 8 years of formal schooling followed by 15 months of training to deliver maternal and child health (MCH) care in community settings. During a monthly home visit, the LHW provide essential maternal and child health care services including family planning needs, nutritional assessments of both mother and child, management of minor and common illnesses, improving immunization coverage and imparting health education. [12]

This study recognizes the LHW as a powerful conduit for reaching under 5 children at household level and identifying severe pneumonia in under five at an early stage. It also recognizes the underestimation of hypoxemia (SpO_2 of <90%), a major risk factor for pneumonia mortality at community level and assesses its prevalence. The reported prevalence of hypoxemia in under 5 acutely ill children is 5 to 58% in facilities [13-16] and 16 to 39% in community settings. [17] A 4.3 times higher risk of mortality has been associated with pneumonia with hypoxemia than in children with pneumonia without hypoxemia. [13] Hypoxemia is also predictive of treatment failure with amoxicillin in 3-59 months old children. [18] In resource poor settings where pulse oximetry is not feasible, signs and symptoms of severe pneumonia (sleepiness, cyanosis, head

For peer review only - http://bmjopen.bmj.com/site/about/guidelines.xhtml

nodding/grunting, and inability to move) are used as predictors of hypoxemia. However, the validity of clinical signs to predict hypoxemia varies and it is often difficult for physicians working in settings, where objective detection of hypoxemia is not available, to decide whether the child coming with severe pneumonia requires administration of oxygen or not. [15, 19, 20]

Pulse oximetry [16] is a rapid, portable, non-invasive and accurate method of measuring arterial hemoglobin oxygenation (Sp02) and has therefore been used in trial and clinical settings to detect hypoxemia. Appropriate oxygen therapy (based on PO findings rather than clinical signs of severity alone) has been associated with lower mortality risk. [19] Assuming access to supplemental oxygen, PO could potentially avert up to 148,000 severe pneumonia related deaths if implemented, and, combining PO with IMCI assessment for pneumonia has been shown to be cost effective in 15 high burden countries. [21] Emdin et al found first level Lady Health Workers in peri-urban Karachi could easily perform pulse oximetry on young infants on well and sick visits to a primary health care facility. [22]

Over the past decade, the possible impact of pulse oximetry in hospital and community settings has been of interest across the globe. Health survey of 54 countries in 2010, suggested that 19.2% of the operating theatres around the globe are not equipped with pulse oximeters [23]. Trials assessing utility of pulse oximetry on a health systems level in Nigeria have shown that health workers reserve PO for the sickest patients [24, 25]. This has also been observed in areas at higher altitudes with higher prevalence of hypoxemia (highlands of Papua New Guinea) [25, 26]. There is lack of clarity about how pulse oximetry can be used in the community where lower hypoxemia prevalence may be a lesser incentive for health workers to use PO such as in interior Sindh settings in Pakistan. Then again, health workers recruited from within communities may be better invested and motivated than hospital personnel in following case management guidelines precluding PO. It is therefore worthwhile to explore and describe contexts behind a family's acceptance (or not) of referral advice whether based on technology and/or clinical examination in rural settings where the highest burden of pneumonia deaths lies.

Feasibility and sustainability audits of oxygen delivery systems in the Gambia and Egypt have shown that providing technology alone is ineffective, and should preclude provision of supplies, education, training and feedback [27-30]. This protocol paper describes a study to assess the

For peer review only - http://bmjopen.bmj.com/site/about/guidelines.xhtml

effect of PO monitoring in community settings on hospital referral acceptance in children under 5 with severe pneumonia.

Methods

Study aims and design:

The overall aim of the study is to the impact of pulse oximetry on hospital referral acceptance in children under 5 with severe pneumonia in rural Pakistan, district Jamshoro.

Specific objectives are:

1) To assess and compare the impact of 'pulse oximetry' used by LHWs at household level on increasing hospital referral acceptance rates in intervention clusters (district Jamshoro) for 0-59 months old children with severe pneumonia with the impact of LHWs using clinical signs alone in non-intervention clusters of the same district
2) To determine prevalence of severe pneumonia ± hypoxemia in 0-59-month olds
3) Investigate the likely predictors (demographic, clinical) of hospital referral acceptance in both the groups
4) To compare clinical outcomes (treatment completion, treatment failure, hypoxemia, duration of hospital) of children 0-59 months admitted with severe pneumonia who accepted hospital referral to those who refused admission and were treated at home

A cluster randomized study will be conducted within the community in district Jamshoro, among children of ages 0-59 months **(Figure 1)**. Cases will be children with the signs and symptoms of severe pneumonia. Data will be collected on demographics, likely predictors, and clinical outcomes using a structured questionnaire.

The primary outcome is hospital referral acceptance in children under 5 with severe pneumonia and to identify demographic and clinical predictors of hospital referral acceptance. The predictors will include distance of child home to referral facility, socio-economic status of household, parental education, child age, nutritional status, respiratory rate, temperature, hypoxemia and presence of other illnesses.

For peer review only - http://bmjopen.bmj.com/site/about/guidelines.xhtml

Secondary outcomes include duration of oxygen therapy, treatment failure, duration of hospital stay, vital and health status of child at day 7th and 14th.

Trial setting:

The study will be conducted within the community of Taluka Kotri in District Jamshoro, Sindh **(Figure 2)**. Jamshoro District has a population of 993,142 [31]. It is predominantly rural, with 33% literacy, and >50% employed daily wage laborers. A baseline survey conducted as a part of current study showed the status of overall health indicators: skilled birth attendance 57%, antenatal care coverage 75%, postnatal care cover for mother and newborn within 48 hours 31.5%, vaccination completeness in children 12-23 months 68% and care seeking for ARI and diarrhea >80% (internal survey). A total of 27 health facilities function in the district including one District Headquarter (DHQ) Hospital, three Taluka Headquarter (THQ) Hospitals, five Rural Health Centers (RHCs) and 18 Basic Health Units (BHUs). The district is divided administratively in 30 union councils. Kotri is one of the 4 Talukas of Jamshoro, consists of 44% (437,561) of the population of district [31].

Participants will be the permanent residents of Kotri, Jamshoro and recruited from their homes during routine monthly visits by Lady Health Workers serving in their catchment areas. This study is expected to run for 48 months with participant identification and enrolment conducted simultaneously in intervention and control clusters over 21 months after an initial pilot of one month. Each enrolled child will be revisited at day 7 and 14 for outcome measurement.

Participant:

Case will be defined as a child aged 0-59 months having signs and symptoms of severe pneumonia at the time of screening (Appendix 1). Exclusion criteria will be the lack of consent from a guardian of the child to participate in the study.

Randomization and masking:

Study clusters were defined as the area covered by an LHW. Each LHW covers a minimum of 100 households. A list of LHWs working in Kotri was collected from the LHW program, Health Department Government of Sindh. There are a total of 188 active LHWs in the study site. A baseline survey was conducted to collect data on health indicators from the LHW catchments. The clusters were randomly allocated to intervention and control groups on 1:1 fashion with a

For peer review only - http://bmjopen.bmj.com/site/about/guidelines.xhtml

computer-generated randomization sequence that was generated by an independent expert. Clusters were matched on under-5 population and distance to referral health facility. No stratification was used for allocation; clusters were selected to ensure that the reporting and training centres of intervention and control LHWs were separate. The investigators and the national and provincial LHW program coordinators will be excluded from the allocation process.

Participant Recruitment and Study Procedures:

Intervention delivery

Children aged 0–59 months with cough and/or difficult breathing during regular home visit (Appendix 2) will be assessed by LHWs for signs and symptoms of severe pneumonia (fast breathing/chest in-drawing and one or more danger sign (unable to eat/drink, vomiting, convulsion and lethargy/unconsciousness) and/or Stridor)) and hypoxemia (SpO2 <92%) using a handheld pulse oximeter (Masimo Rad-5v) to measure blood oxygen level. LHWs will also do case management of children with pneumonia and severe pneumonia. A 3-day course of oral amoxicillin will be given to children with pneumonia at home, whereas children with severe pneumonia and or hypoxemia (eligible for recruitment) will be requested for informed consent **(Appendix 3)** and offered stat dose of oral amoxicillin and referral to nearest referral hospital (DHQ Kotri).

The study investigators will have provided these pulse oximeters to the LHW Program in advance and highlighted which ones will receive them. Physicians at the referral center serving the intervention clusters will also receive handheld pulse oximeters. All the LHWs and staff will be trained on the use, and maintenance of these pulse oximeters. Children with severe pneumonia with or without hypoxemia will be advised to go to hospital for antibiotics and oxygen, using the PO reading as a tool to convince parents. Name of the pre-designated health facility with available oxygen and study physician will be provided to all the LHWs so that follow up visits can be made to ensure the patient receives safe and recommended care at referral facility. Project staff will pretest and regularly monitor PO accuracy and quality of readings.

Hypoxemia will be defined as an arterial oxygen saturation (SpO2) <92%. SpO2 measurement will be recorded after 1 minute of stable observation. If the SpO2 comes 92% or less, the child will first be assessed for nasal obstruction with readings repeated after applying nasal saline drops. If repeat reading shows hypoxemia, the child will be referred to nearest designated referral

For peer review only - http://bmjopen.bmj.com/site/about/guidelines.xhtml

hospital and admitted for oxygen via nasal or nasopharyngeal route and intravenous antibiotics, as per recommendations.

Implementation of active control: Clinical Signs assessment

Children aged 0–59 months with cough and/or difficult breathing during regular home visit will be assessed by LHWs for signs and symptoms of severe pneumonia (fast breathing/chest in-drawing and one or more danger sign (unable to eat/drink, vomiting, convulsion and lethargy/unconsciousness) and/or Stridor)). A 3-day course of oral amoxicillin will be given to children with pneumonia at home, whereas children with severe pneumonia (eligible for recruitment) will be requested for informed consent **(Appendix 3)** and offered stat dose of oral amoxicillin and referral to nearest referral hospital.

Procedure at referral facility

Children who accept hospital referral in both intervention and control clusters, and reach hospital premises with LHW referral slip will be assessed by study nurse at the referral center (Appendix 2). An SMS notification with brief details of referred child will have been provided to trained study personnel (study physician/nurse) in advance at time of referral at both the referral facilities. Children with severe pneumonia and/or hypoxemia who reach referral hospital premises will be examined and subjected to pulse oximetry again. They will be admitted for further appropriate treatment (Oxygen therapy via nasal or nasopharyngeal route and intravenous antibiotics etc.). All the children at referral facility will also undergo 12 hourly monitoring by study personnel and filling of case reporting form (CRF) and hospital physician form (HPF) at day 1, 7 and 14. Those children who refused the referral will be visited by study community health workers after 24 hours to confirm referral refusal and to fill CRF.

Preliminary meetings will be held with the Executive Director Health Jamshoro, Director General Health Sindh, In-charge Lady Health Worker Program Sindh and In-charge Pediatric Units LUMHS to ensure their cooperation through study duration. Emergency and pediatric unit staff at the referral facilities along with study personnel (physician/nurse) will be trained on management of severe pneumonia according to the integrated management of neonatal and childhood illnesses (IMNCI) guidelines [32]. A baseline survey will be conducted at the health facilities to ensure availability of oxygen and necessary intravenous antibiotics. Even though it is ideal to guarantee sustainable oxygen systems at the two chosen referral public sector hospitals,

For peer review only - http://bmjopen.bmj.com/site/about/guidelines.xhtml

this study does not provide oxygen and therefore aims to assess 'real-life' situations in public hospitals and their impact on severe pneumonia outcomes with or without hypoxemia. LHWs will be incentivized on basis of their contribution to the study activities.

Data Collection and Storage

Data will be collected by LHWs during house visits (Screening form), community health workers (during follow up visits day 1, 7 and 14) and hospital based study personnel (for all who accept referral and reach hospital premises) on paper forms **(Appendix 2)**. Given that it will be a new experience for LHWs to assess, classify and manage ARI cases and at the same time record findings on data forms accurately, these will be supervised closely and frequently, at least for the first pneumonia season. Well trained study field supervisory officers and LHW supervisors will be required to perform regular field supervision in their respective clusters and ensure accurate and logically entered data forms and make necessary verifications and corrections at the data collection sites and give feedback to the LHW to avoid repeating the errors. Raw data brought to the program office will be checked once again for accuracy by the technical staff and approved for entry in the computer. All raw data will be safely kept in the AKU office, appropriately numbered by cluster, until seven years after the study is over.

Case History Records

These include the study case report forms (CRF) and hospital physician form (HPF) that will contain information that documents the child's eligibility to participate in the study, the signed consent form, and information from tests and examinations. Wherever possible copies of supporting documentation for the information contained in the CRF should be kept with each patient's case history record. This supporting documentation may include records of physical examinations, progress notes, laboratory reports, X-rays, consultations, correspondence, information and data on the subject's condition, during and after the clinical investigation, diagnoses made, concomitant therapy, etc. All information in the case history records should be attributable to a specific individual. Since the CRF will not contain the patient's name, there will be a unique link between the ID number on the CRF and the patient's name. Each child's case history record will be evaluated to verify validity and completeness of the data on the CRF when a study monitor visits the study site. All corrections to CRF's must be made without obscuring the original entry. The revised entry should be inserted and the person making the correction

For peer review only - http://bmjopen.bmj.com/site/about/guidelines.xhtml

should sign and date the correction. Only authorized study personnel may complete or correct case report forms.

Data Management

Screening data will be collected on paper by LHWs. Case reporting form (CRF) and Hospital physician form will be collected on electronic forms. To ensure proper implementation of the intervention, the field supervisors will make spot checks and will arrange monthly refresher group sessions of the first-line health workers in which the problems encountered will be discussed and resolved. In addition, the data collection activity will be carried out by teams consisting of LHWs/CHWs and study staff will be further monitored by field supervisors who will perform a check on a subset (5%) of households.

An information system will be set up to keep track all patients screened and enrolled and a filing system to keep all study related records - case history records, study protocol or related documentation and drug distribution records. The coordinator at the site will be responsible for the completeness and accuracy of all the study materials.

Supervision of Data Collection

The Project Manager is responsible for oversight of the completion of all appropriate case report forms up to the time that the child is discontinued from the study. Each completed form will be submitted monthly and assessed for completeness by the Project Manager. Inconsistencies, omissions and obvious errors will be compared against the LHW clinical log and corrected. Study data will be collected on case report forms (CRF). At least once a month, the Project Manager or his/her designate must contact the designated person to report on the number of new children enrolled in the study and the status of all enrolled study children. Study specific forms (paper and electronic versions) will be provided to each site. Each month, the Project Manager or his/her designate will be responsible for visually editing the CRF for each child who has completed or withdrawn from the study. All missing data should be identified, and every attempt should be made to complete missing data.

Study Protocol and Related Documentation

All study related documents including the study protocol, manuals of operations, all correspondence sent to or received from the study monitor, materials used for obtaining informed

For peer review only - http://bmjopen.bmj.com/site/about/guidelines.xhtml

consent, protocol modifications and records of the Institutional Review Board approval and all communications with the IRB must be maintained in complete form. These documents will be evaluated to ensure that study documentation is complete and current when a study monitor visits the study site.

Record Retention

Retention of accurate and complete records is essential to establish the validity and completeness of the study. All records must be retained for 7 years after the data set is frozen. Electronic data will be de-identified, unlinked from any personal identifiers and therefore will protect individual identity.

Reporting of Serious Adverse Events and Treatment Failures

Amoxicillin is in widespread use and is not investigational in any study site. However, since oral amoxicillin is not routinely recommended for initial treatment of children who have severe pneumonia, the appropriate case report forms describing the occurrence of a serious adverse event, treatment failure or death must be faxed to the coordinating center within 72 hours of the site coordinator knowing about the event. Adverse events, treatment failure and death must be reported to allow appropriate interpretation of this critical information. If the outcome of the adverse event is unknown when the site coordinator first notifies the coordinating center, a follow-up form must be faxed to the coordinating center within 10 days of knowing about the event. The PI should send a copy of the adverse event data to their local IRB as soon as possible. The coordinating center will summarize the Adverse Event and Death information and send a report to the IRB/ERC of sponsors and to site IRBs. Both the rate of adverse events and the rate of patient accrual at each individual site will be monitored to determine if stopping rules are met. We do not anticipate serious adverse events. However, in case of one, a DSMB will be requested for and convened on ad hoc basis for safety review at any time during the study if there is a concern regarding rates of adverse events or rates of patient accrual. Adverse events will be reported by the study physician to the principal investigator and clinically managed by the study physician in conjunction with other physicians at the institution. Any related and unexpected life-threatening adverse event including death will be reported to the IRB within 2 business days as per IRB protocol and any related, unexpected and serious adverse event will be reported to the IRB within 10 business days as per IRB protocol.

For peer review only - http://bmjopen.bmj.com/site/about/guidelines.xhtml

Compliance with and Deviations from the Study Protocol

The Site coordinator must agree with and sign the protocol and confirm in writing that he or she has read, understands and will work according to the protocol and Good Clinical Practice. The Site coordinator is responsible for making sure that the protocol is strictly followed and should not make any changes to the study unless necessary to eliminate an apparent immediate hazard or damage to a trial subject. Any deviations from the study protocol including but not limited to inappropriate enrollment of a study subject, administration of the wrong study treatment, missed doses of study treatment, missed observation points, incorrect administration of concomitant medications, etc. should be reported to the coordinating center and each site's IRB. The report should include a plan to rectify any problems at the site that may have caused the protocol deviation.

Sample size:

Defining a cluster as (the catchment area of) one Lady Health Worker (LHW), and assuming a power of 90% in detecting 50% increase in referral acceptance from a 10 % baseline to 15% among 2-59 month old children with severe pneumonia (pneumonia prevalence at 2 week recall (MICS Sindh):7.5% (18% of which is assumed severe Pneumonia) with ICC 0.001736. We need to capture a total of 4160 children with severe pneumonia in both intervention and control groups.

Data Analysis:

The primary analysis for each outcome will perform on an 'intention-to-treat' (ITT) basis, i.e. all children included in the analysis who were enrolled in the study according to the group to which they were allocated. All analyses will account for the cluster-randomized design to ensure correct type I error rates and confidence intervals [33]. Baseline characteristics will be compared by analyzing differences in means and proportions among the study arms. Categorical outcomes will be compared using chi square test and continuous outcomes using Student's t-test. For analysis of predictors of referral acceptance, generalized linear model will be used with logit link function. The univariate analysis will be conducted to explore the independent effect of each predictor on outcome. The variables significant at a liberal p-value of <0.20 will be included in multivariate model for adjustment. The results will be reported as relative risk (RR) with 95%

For peer review only - http://bmjopen.bmj.com/site/about/guidelines.xhtml

CI. Type 1 error will be set at 5% level. Interim analysis will be conducted after completion of 50% of the data collection. All analysis will be done using STATA version 15.

Patient and Public Involvement

Patients or the public will not be involved in the design or conduct of the study. Results will be disseminated to the community.

Study Status

Recruitment began in August 2019 and field activities and data collection are in process. As of 16th October 2020, a total of 235 cases and 184 controls have been enrolled. Recruitment is scheduled to end by December 2021.

Discussion

Hypoxemia, a frequent complication of severe pneumonia, is a major risk factor for death in children under 5. Theoretically, detection of hypoxemia at community level among severe pneumonia cases by the use of pulse-oximeter would give awareness to the caregivers about severity of illness and reduce delay in hospital referral. Delayed care-seeking is a recognized risk factor in pneumonia mortality in community settings [34]. Unfortunately, information on prevalence of hypoxemia, effectiveness of its detection in influencing parents to seek hospital care (gold standard for severe pneumonia), and impact of appropriate care at hospital in settings like Pakistan is lacking. Thus, the findings of this study will build evidence for utility of providing front-line workers like LHWs with a tool to detect hypoxemia if signs and symptoms of pneumonia are present. Interestingly various groups across the world are now espousing pulse-oximetry with other strengths like detection of congenital heart disease [35, 36] and newborn sepsis [37]. While recognizing the importance of detection of hypoxemia is extremely important, true impact on pneumonia survival cannot be made without improving oxygen systems and protocols for use at referral centers. Duke et al showed improved case fatality rates by providing oxygen concentrators and pulse-oximeters at five hospitals in PNG along with protocols for use [33].

If our study reveals pulse-oximetry has influenced health-seeking behavior significantly, we will need to follow with a more systematic evaluation of pneumonia care at rural hospitals which vary in quality of care. We will also need to correlate recovery rates in those who accepted

For peer review only - http://bmjopen.bmj.com/site/about/guidelines.xhtml

hospital referral versus those who stayed home on oral amoxicillin. Evidence to support home care for severe pneumonia is poor [38].

Ethics and Dissemination

This study has been approved by the Ethical Review Committee of The Aga Khan University (4722-Ped-ERC-17), Karachi, Pakistan in June 2017. Written informed consent in the local language will be obtained from parents or guardians of all participants. Data forms will contain no identifying information other than age, sex and GIS coordinates. Laboratory forms will contain no identifying information, specimens will be identified by a study number only and test results will not be linked to any individual by name. All survey staff will sign a confidentiality agreement to ensure that they do not release participant identities and test or study results to individuals who are not part of the study team.

Study progress and findings will be shared with sponsors (BMGF) quarterly. Results will be presented at national and international research meetings and conferences and also prepared for publication in international peer-reviewed scientific journals. Study findings will be disseminated to the study communities.

Declarations:

Consent for publication: Yes

Availability of data and materials: The datasets used for the article and the study is available from the corresponding author on request.

Competing interests: The authors declare that they have no competing interests.

Funding: The study is funded by Bill & Melinda Gates Foundation through Grant OPP1148892.

Author Contributions: FM, ZM, MAH, SBS & ZAB: conceptualization of project. FM, AAN, and SC: development of study design and questionnaires. AAN and SC: oversight of data collection. AAN, SC and AH: support of study logistics and field activities. IA and AR: statistical analyses. All authors have read and approved the final manuscript.

Acknowledgements: We would like to acknowledge the mothers and families who have contributed to the study. We are grateful to the Sindh LHW Program, Department of Health for their support and facilitation of the trial.

For peer review only - http://bmjopen.bmj.com/site/about/guidelines.xhtml

For peer review only

For peer review only - http://bmjopen.bmj.com/site/about/guidelines.xhtml

Figures:

Figure 1: *Trial Profile*

Figure 2: *Study intervention and control sites*

References

1. Liu, L., et al., *Global, regional, and national causes of child mortality: an updated systematic analysis for 2010 with time trends since 2000.* Lancet, 2012. **379**(9832): p. 2151-61.
2. Rudan, I., et al., *Epidemiology and etiology of childhood pneumonia.* Bull World Health Organ, 2008. **86**(5): p. 408-16.
3. *WHO/UNICEF Joint Statement: Management of Pneumonia in community settings.* 2002.
4. *Ending Preventable Child Deaths from Pneumonia and Diarrhoea by 2025: The integrated Global Action Plan for Pneumonia and Diarrhoea (GAPPD)*. 2013, World Health Organization: Geneva, Switzerland.
5. Soofi, S., et al., *Effectiveness of community case management of severe pneumonia with oral amoxicillin in children aged 2-59 months in Matiari district, rural Pakistan: a cluster-randomised controlled trial.* Lancet, 2012. **379**(9817): p. 729-37.
6. Kallander, K., D.H. Burgess, and S.A. Qazi, *Early identification and treatment of pneumonia: a call to action.* Lancet Glob Health, 2015. **4**(1): p. e12-3.
7. Owais, A., et al., *Why do families of sick newborns accept hospital care? A community-based cohort study in Karachi, Pakistan.* J Perinatol, 2011. **31**(9): p. 586-92.
8. Zaidi, A.K., et al., *Community-based treatment of serious bacterial infections in newborns and young infants: a randomized controlled trial assessing three antibiotic regimens.* Pediatr Infect Dis J, 2012. **31**(7): p. 667-72.
9. Ferdous, F., et al., *Mothers' perception and healthcare seeking behavior of pneumonia children in rural bangladesh.* ISRN Family Med, 2014: p. 690315.
10. Waiswa, P., et al., *Using the three delays model to understand why newborn babies die in eastern Uganda.* Trop Med Int Health, 2010. **15**(8): p. 964-72.
11. *LADY HEALTH WORKER PROGRAM FOR FAMILY PLANNING AND PRIMARY HEALTH CARE*. Available from: https://www.sindhhealth.gov.pk/LHW-Program.
12. Afsar, H., et al., *Factors affecting unsuccessful referral by the lady health workers in Karachi, Pakistan.* Journal-Pakistan Medical Association, 2003. **53**(11): p. 521-528.
13. Onyango, F.E., et al., *Hypoxaemia in young Kenyan children with acute lower respiratory infection.* BMJ, 1993. **306**(6878): p. 612-5.
14. Smyth, A., H. Carty, and C.A. Hart, *Clinical predictors of hypoxaemia in children with pneumonia.* Ann Trop Paediatr, 1998. **18**(1): p. 31-40.
15. Usen, S., et al., *Clinical predictors of hypoxaemia in Gambian children with acute lower respiratory tract infection: prospective cohort study.* BMJ, 1999. **318**(7176): p. 86-91.
16. McCollum, E.D., et al., *Multicenter study of hypoxemia prevalence and quality of oxygen treatment for hospitalized Malawian children.* Trans R Soc Trop Med Hyg, 2013. **107**(5): p. 285-92.
17. Basnet, S., R.K. Adhikari, and C.K. Gurung, *Hypoxemia in children with pneumonia and its clinical predictors.* Indian J Pediatr, 2006. **73**(9): p. 777-81.
18. Fu, L.Y., et al., *Brief hospitalization and pulse oximetry for predicting amoxicillin treatment failure in children with severe pneumonia.* Pediatrics, 2006. **118**(6): p. e1822-30.
19. Duke, T., et al., *Hypoxaemia in acute respiratory and non-respiratory illnesses in neonates and children in a developing country.* Arch Dis Child, 2002. **86**(2): p. 108-12.
20. Weber, M.W., et al., *Predictors of hypoxaemia in hospital admissions with acute lower respiratory tract infection in a developing country.* Arch Dis Child, 1997. **76**(4): p. 310-4.

For peer review only - http://bmjopen.bmj.com/site/about/guidelines.xhtml

21. Floyd, J., et al., *Evaluating the impact of pulse oximetry on childhood pneumonia mortality in resource-poor settings.* Nature, 2015. **528**(7580): p. S53-9.
22. Emdin, C.A., et al., *Utility and feasibility of integrating pulse oximetry into the routine assessment of young infants at primary care clinics in Karachi, Pakistan: a cross-sectional study.* BMC Pediatr, 2015. **15**: p. 141.
23. Funk, L.M., et al., *Global operating theatre distribution and pulse oximetry supply: an estimation from reported data.* The Lancet, 2010. **376**(9746): p. 1055-1061.
24. Ingram, G. and N. Munro, *The use (or otherwise) of pulse oximetry in general practice*. 2005, British Journal of General Practice.
25. Mokuolu, O.A. and O.A. Ajayi, *Use of an oxygen concentrator in a Nigerian neonatal unit: economic implications and reliability.* Ann Trop Paediatr, 2002. **22**(3): p. 209-12.
26. Matai, S., et al., *Implementing an oxygen programme in hospitals in Papua New Guinea.* Ann Trop Paediatr, 2008. **28**(1): p. 71-8.
27. Dobson, M., D. Peel, and N. Khallaf, *Field trial of oxygen concentrators in upper Egypt.* Lancet, 1996. **347**(9015): p. 1597-9.
28. Enarson, P., et al., *Implementation of an oxygen concentrator system in district hospital paediatric wards throughout Malawi.* Bull World Health Organ, 2008. **86**(5): p. 344-8.
29. Hill, S.E., et al., *Oxygen for treatment of severe pneumonia in The Gambia, West Africa: a situational analysis.* Int J Tuberc Lung Dis, 2009. **13**(5): p. 587-93.
30. Howie, S.R., et al., *Meeting oxygen needs in Africa: an options analysis from the Gambia.* Bull World Health Organ, 2009. **87**(10): p. 763-71.
31. Pakistan, P.B.o.S.G.o. *PROVINCE WISE PROVISIONAL RESULTS OF CENSUS - 2017*. 2017; Available from: http://www.pbs.gov.pk/content/block-wise-provisional-summary-results-6th-population-housing-census-2017-january-03-2018.
32. *Integrated Management of Childhood Illness (IMCI) (revised)*. 2014, Geneva: World Health Organization/The United Nation Children's Fund (UNICEF).
33. Duke, T., et al., *Improved oxygen systems for childhood pneumonia: a multihospital effectiveness study in Papua New Guinea.* The Lancet, 2008. **372**(9646): p. 1328-1333.
34. Ferdous, F., et al., *Pneumonia mortality and healthcare utilization in young children in rural Bangladesh: a prospective verbal autopsy study.* Tropical medicine and health, 2018. **46**(1): p. 1-10.
35. Engel, M.S. and L.K. Kochilas, *Pulse oximetry screening: a review of diagnosing critical congenital heart disease in newborns.* Medical Devices (Auckland, NZ), 2016. **9**: p. 199.
36. Tekleab, A.M. and Y.C. Sewnet, *Role of pulse oximetry in detecting critical congenital heart disease among newborns delivered at a high altitude setting in Ethiopia.* Pediatric Health, Medicine and Therapeutics, 2019. **10**: p. 83.
37. Jawin, V., et al., *Beyond critical congenital heart disease: newborn screening using pulse oximetry for neonatal sepsis and respiratory diseases in a middle-income country.* PLoS One, 2015. **10**(9): p. e0137580.
38. Das, R.R. and M. Singh, *Treatment of severe community-acquired pneumonia with oral amoxicillin in under-five children in developing country: a systematic review.* PloS one, 2013. **8**(6): p. e66232.

For peer review only

For peer review only - http://bmjopen.bmj.com/site/about/guidelines.xhtml

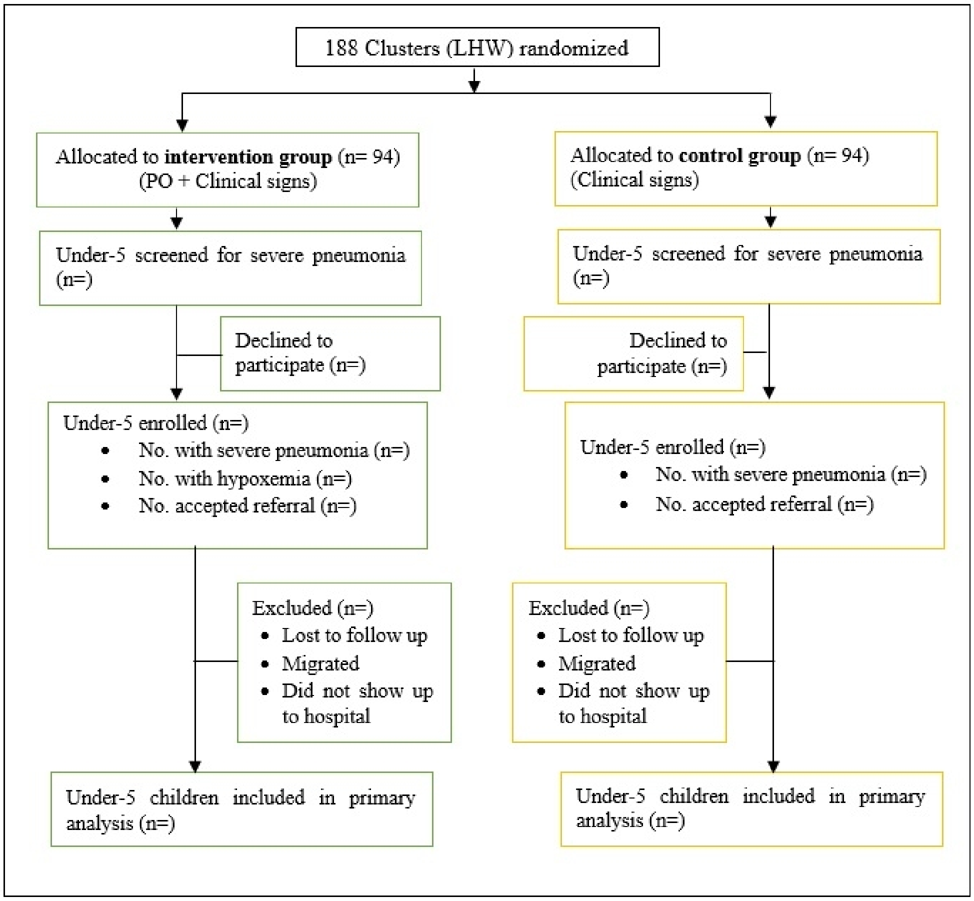

Figure 1: Trial Profile

185x168mm (96 x 96 DPI)

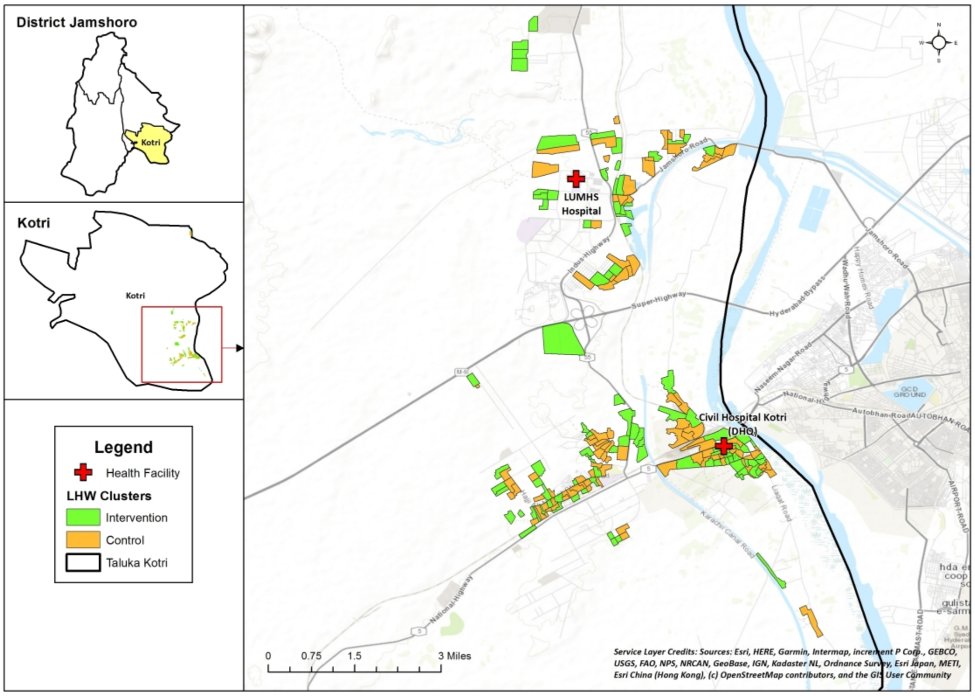

Figure 2: Study Intervention and Control Sites

296x210mm (300 x 300 DPI)

Appendix 1: Case definition of Severe Pneumonia

Case Definition of Severe Pneumonia	
**Intervention Group**	**Control Group**
**Children of 0 – 6 days:**<br>**1.** Fast Breathing (≥ 60 breaths/min)<br>*+/-*<br>***2. Hypoxemia (<92 SpO2 Blood Oxygen Level)***	**Children of 0 – 6 days:**<br>1. Fast Breathing (≥ 60 breaths/min)
**Children of 7 days – 59 months:**<br>**1.** Fast Breathing and/or Chest In-drawing<br>➢ 0-2months: ≥ 60 breaths/min<br>➢ 2-12months: ≥50 breaths/min<br>➢ 12-59months ≥40 breaths/min<br>+<br>**2.** Any ONE General Danger Sign* and/or Stridor<br>➢ *Unable to drink/eat<br>➢ Vomiting<br>➢ Convulsions<br>➢ Lethargy/Unconsciousness<br>*+/-*<br>***3. Hypoxemia (<92 SpO2 Blood Oxygen Level)***	**Children of 7 days – 59 months:**<br>1. Fast Breathing and/or Chest In-drawing<br>➢ 0-2months: > 60 breaths/min<br>➢ 2-12months: ≥50 breaths/min<br>➢ 12-59months ≥40 breaths/min<br>+<br>2. Any ONE General Danger Sign* and/or Stridor<br>➢ *Unable to drink/eat<br>➢ Vomiting<br>➢ Convulsions<br>➢ Lethargy/Unconsciousness

For peer review only

Appendix 2: Study Activities

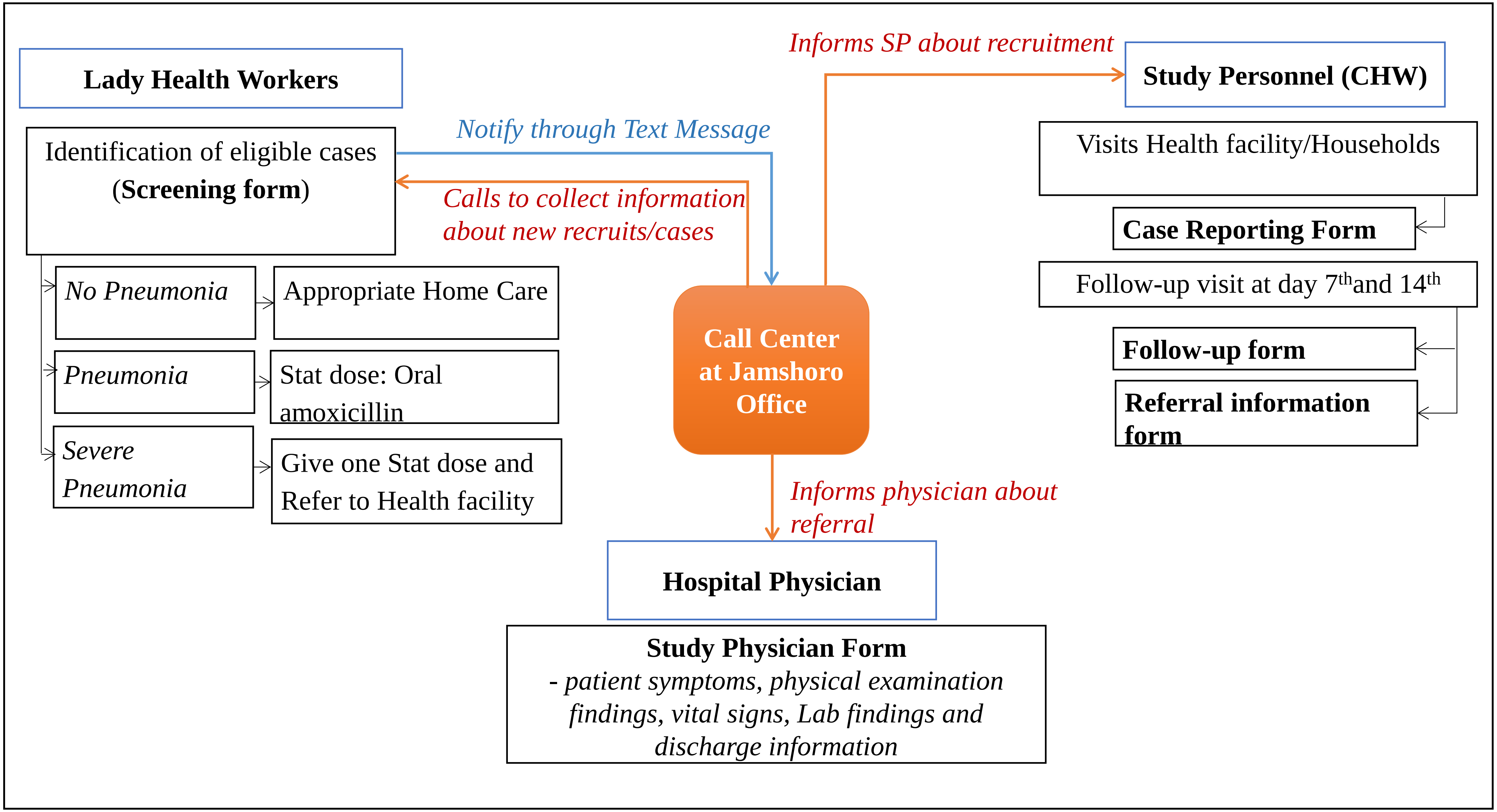

Appendix 3: Informed Consent

Research Consent Form

Title of Research Project:

Impact of Pulse Oximetry on Hospital Referral Acceptance in children under 5 with severe pneumonia in rural Pakistan (District Jamshoro): a cluster randomized trial (GAPPD Scale up Project)

Investigators:

Dr. Fatima Mir (Principal investigator PO Project, AKU)

Department of Pediatrics and Child Health, Aga Khan University, Karachi, Pakistan

Tel: 92-21-34864955

Dr. Sajid Soofi (PI, Scale up of GAPPD in Pakistan)

Centre of Excellence in Women & Child Health, The Aga Khan University, Pakistan.

Tel: 92-21-34864955

Professor Zulfiqar Bhutta (Senior Investigator, Scale up of GAPPD in Pakistan)

Centre of Excellence in Women & Child Health, The Aga Khan University, Pakistan.

Tel: 92-21-34864955

Purpose of the Research:

Severe Pneumonia is a serious and possibly life threatening infection in young children aged under 5 years. It may involve fast breathing, chest indrawing, and danger signs like inability to feed, convulsions, persistent vomiting and decreased movement. Hypoxemia is now recognized as an additional sign of severe pneumonia needing hospital admission. Pulse oximetry (the 'oxygen test') is a simple method whereby lady health workers can check level of oxygen in blood of children and decide which young babies need immediate medical care and referral to hospital for antibiotics and oxygen and which babies can be treated in the health center itself.

In this study, we are testing whether use of pulse oximetry in addition to other danger signs of severe pneumonia will influence families to accept hospital referral in comparison to areas where danger signs other than hypoxemia are used.

This project is a collaboration between Aga Khan University (AKU) in Karachi and the Gates Foundation, Seattle, USA.

For peer review only

Description of the Research:

We are inviting children aged 0 to 59 months screened at home to join this research study. If your baby joins the study, the following activities will take place:

1) **Questionnaire**. We will ask several questions about the health of your baby, as well as check him/her for presence of pneumonia. If he/she has signs of severe pneumonia (with or without hypoxemia), he will be eligible to take part in our study.

2) **Pulse oximetry**. Next your child may or may not undergo a pulse oximetry measurement dependent on whether he resides in an intervention or non-intervention cluster. The process will be painless for the child and will involve placing a sensor on your baby's foot or hand. Each sensor is attached to a pulse oximetry machine. A number on the screen of the machine shows the oxygen level. A level including and above 92% will be regarded as normal.

   All babies with fast breathing or chest in drawing pneumonia AND any one of general danger signs (inability to feed, persistent vomiting, decreased movement, convulsions) with or without hypoxemia (SaO2 <92%) will be advised and helped to go to an assigned hospital for treatment (antibiotics and possibly oxygen supportive therapy). Their clinical status over there will be followed and transfer to hospital and subsequent treatment will be facilitated by the study.

   All responses will be documented on paper forms. We will arrange for quick referral to hospital if your baby has low oxygen level or other signs of serious illness. Follow-up visits by our study staff will be conducted to check the status of your baby.

3) You may be asked detailed questions based on whether you accept or refuse hospital referral to allow us to understand what factors contributed to your decision making. Since this process will take time, we will do it after the child's treatment plan has been started at hospital or at home.

4) **Video recording**. Some infants will be video recorded during the study. The researchers will look at these videos to make quality checks on study conduct and procedures. You can refuse video recording but still join the rest of the study. If you provide permission, some videos may be shown publicly for education purposes, but your child's name will be kept private. The videos or images from them will not be shown or sold for financial profit.

5) **Stored information**. This study is connected to other studies coordinated by the Aga Khan University. If you agree to join this study, we will access information about your baby collected as part of the Aga Khan University demographic surveillance system and other Aga Khan University studies to which you have already allowed, or will allow, your baby to join.

We expect to enroll about 4160 children with severe pneumonia in this study.

Potential Harms, Discomforts or Inconveniences:

There are no harms or discomforts that could be caused to your baby by taking part in this study.

Potential Benefits to individual participants:

Your baby may benefit by joining this study as we will provide facilitated transfer to hospital and free treatment at the hospital if referral is accepted. If you refuse to accept hospital referral for injectable antibiotics and oxygen, we will provide less ideal oral treatment options at home.

Potential Benefits to Society:

We may learn more about the usefulness of pulse oximetry for assessment of young babies in your community and other similar places.

For peer review only

Confidentiality:

We will respect your privacy. No information about you or your child will be given to anyone or be published without your permission, unless required by law. The paper forms, videos and electronic information made in this study will be stored in a secure, locked location. Only members of the research team will have access to them. BMGF (the sponsor) or AKU Clinical Research Monitors may look at your child's records to check on the study. By signing this consent form, you agree to let these people look at your child's records. We will put a copy of this research consent form in your child's patient health record and give you a copy as well. After the study has been completed, the forms and videos will be kept as long as required by BMGF and AKU policies. They will then be destroyed according to these same policies. Paper and electronic forms from this study will be stored for at least 7 years after publication of the study. Published study results will not reveal your identity or the identity of your baby.

Payment

No payment will be given for participation in this study. However, if your baby requires referral to hospital, we will pay for the transportation and medical care at the hospital.

Participation:

You decide if your child joins this study. If you choose to let your child take part, you can take your child out of the study at any time.

In the unlikely situation that your child becomes ill or is harmed because of study participation, we will treat your child for free. Your signing this consent form does not interfere with your legal rights in any way. The staff of the study, any people who gave money for the study, or the hospital are still responsible, legally and professionally, for what they do during this study.

Sponsorship:

The funder of this research is the Bill and Melinda Gates Foundation Seatttle, USA.

Conflicts of interest:

None of the researchers have any conflicts of interest to declare.

Consent*:*

By signing this form, you agree that:

1) The study has been explained to you.
2) All of your questions have been answered.
3) The possible harms and benefits of this study have been explained to you.
4) You may ask questions about the study now and in the future.
5) You have been told that your child's medical records will be kept private except as described to you.
6) You understand that information about your child will not be given to anyone or be published without first asking your permission.

I agree that my child____ may take part in this study.

I understand that videos may be used to assess the success of pulse oximetry. I understand that I may refuse to permit video-recording of my child, yet take part in other aspects of the study. I understand that even if I permit video-recording now, I may refuse the use of these videos at a later time. I understand that I may permit video-recording of my child for researchers to view, but refuse any public display of the videos.

For peer review only

I agree to permit my child and me to be video-recorded during this study.

☐ YES ☐ NO

I agree to permit whole or parts of videos of my child and me to be shown publicly for educational purposes.

☐ YES ☐ NO

______________________________	______________________________
Printed Name of Parent/Legal Guardian date	Parent/Legal Guardian's signature/Thumb print &
______________________________	______________________________
Printed Name of person who explained consent	Signature of Person who explained consent & date
______________________________	______________________________
Printed Witness' name<br>(If the parent/legal guardian does not read Sindhi)	Witness' signature & date

If you have any questions about this study, please call Dr Fatima Mir at ___________________

If you have questions about your child's rights as a subject in a study or injuries during a study, please call Coordinator, Bioethics Unit at ____________________

For peer review only

SPIRIT 2013 Checklist: Recommended items to address in a clinical trial protocol and related documents*

Section/item	Item No	Description	Page numbers:
**Administrative information**			
Title	1	Descriptive title identifying the study design, population, interventions, and, if applicable, trial acronym	Title page
Trial registration	2a	Trial identifier and registry name. If not yet registered, name of intended registry	Page 1
	2b	All items from the World Health Organization Trial Registration Data Set	Page 1
Protocol version	3	Date and version identifier	
Funding	4	Sources and types of financial, material, and other support	Page 14
Roles and responsibilities	5a	Names, affiliations, and roles of protocol contributors	Title page and page 14
	5b	Name and contact information for the trial sponsor	
	5c	Role of study sponsor and funders, if any, in study design; collection, management, analysis, and interpretation of data; writing of the report; and the decision to submit the report for publication, including whether they will have ultimate authority over any of these activities	
	5d	Composition, roles, and responsibilities of the coordinating centre, steering committee, endpoint adjudication committee, data management team, and other individuals or groups overseeing the trial, if applicable (see Item 21a for data monitoring committee)	
**Introduction**			

For peer review only

Background and rationale	6a	Description of research question and justification for undertaking the trial, including summary of relevant studies (published and unpublished) examining benefits and harms for each intervention	Pages 3 - 5
	6b	Explanation for choice of comparators	Page 4
Objectives	7	Specific objectives or hypotheses	Page 5
Trial design	8	Description of trial design including type of trial (eg, parallel group, crossover, factorial, single group), allocation ratio, and framework (eg, superiority, equivalence, noninferiority, exploratory)	Page 5

Methods: Participants, interventions, and outcomes

Study setting	9	Description of study settings (eg, community clinic, academic hospital) and list of countries where data will be collected. Reference to where list of study sites can be obtained	Page 6
Eligibility criteria	10	Inclusion and exclusion criteria for participants. If applicable, eligibility criteria for study centres and individuals who will perform the interventions (eg, surgeons, psychotherapists)	Page 6
Interventions	11a	Interventions for each group with sufficient detail to allow replication, including how and when they will be administered	Pages 7 - 9
	11b	Criteria for discontinuing or modifying allocated interventions for a given trial participant (eg, drug dose change in response to harms, participant request, or improving/worsening disease)	Not applicable
	11c	Strategies to improve adherence to intervention protocols, and any procedures for monitoring adherence (eg, drug tablet return, laboratory tests)	Not applicable
	11d	Relevant concomitant care and interventions that are permitted or prohibited during the trial	

Outcomes	12	Primary, secondary, and other outcomes, including the specific measurement variable (eg, systolic blood pressure), analysis metric (eg, change from baseline, final value, time to event), method of aggregation (eg, median, proportion), and time point for each outcome. Explanation of the clinical relevance of chosen efficacy and harm outcomes is strongly recommended	Pages 5-6
Participant timeline	13	Time schedule of enrolment, interventions (including any run-ins and washouts), assessments, and visits for participants. A schematic diagram is highly recommended (see Figure)	Page 6
Sample size	14	Estimated number of participants needed to achieve study objectives and how it was determined, including clinical and statistical assumptions supporting any sample size calculations	Page 12
Recruitment	15	Strategies for achieving adequate participant enrolment to reach target sample size	Pages 7 - 9
**Methods: Assignment of interventions (for controlled trials)**			
Allocation:			
Sequence generation	16a	Method of generating the allocation sequence (eg, computer-generated random numbers), and list of any factors for stratification. To reduce predictability of a random sequence, details of any planned restriction (eg, blocking) should be provided in a separate document that is unavailable to those who enrol participants or assign interventions	Pages 6 - 7
Allocation concealment mechanism	16b	Mechanism of implementing the allocation sequence (eg, central telephone; sequentially numbered, opaque, sealed envelopes), describing any steps to conceal the sequence until interventions are assigned	Pages 6 - 7
Implementation	16c	Who will generate the allocation sequence, who will enrol participants, and who will assign participants to interventions	Pages 6 - 8
Blinding (masking)	17a	Who will be blinded after assignment to interventions (eg, trial participants, care providers, outcome assessors, data analysts), and how	

For peer review only - http://bmjopen.bmj.com/site/about/guidelines.xhtml

	17b	If blinded, circumstances under which unblinding is permissible, and procedure for revealing a participant's allocated intervention during the trial	
**Methods: Data collection, management, and analysis**			
Data collection methods	18a	Plans for assessment and collection of outcome, baseline, and other trial data, including any related processes to promote data quality (eg, duplicate measurements, training of assessors) and a description of study instruments (eg, questionnaires, laboratory tests) along with their reliability and validity, if known. Reference to where data collection forms can be found, if not in the protocol	Page 9
	18b	Plans to promote participant retention and complete follow-up, including list of any outcome data to be collected for participants who discontinue or deviate from intervention protocols	Page 12
Data management	19	Plans for data entry, coding, security, and storage, including any related processes to promote data quality (eg, double data entry; range checks for data values). Reference to where details of data management procedures can be found, if not in the protocol	Page 10
Statistical methods	20a	Statistical methods for analysing primary and secondary outcomes. Reference to where other details of the statistical analysis plan can be found, if not in the protocol	Page 12
	20b	Methods for any additional analyses (eg, subgroup and adjusted analyses)	Page 12
	20c	Definition of analysis population relating to protocol non-adherence (eg, as randomised analysis), and any statistical methods to handle missing data (eg, multiple imputation)	
**Methods: Monitoring**			
Data monitoring	21a	Composition of data monitoring committee (DMC); summary of its role and reporting structure; statement of whether it is independent from the sponsor and competing interests; and reference to where further details about its charter can be found, if not in the protocol. Alternatively, an explanation of why a DMC is not needed	Page 10

	21b	Description of any interim analyses and stopping guidelines, including who will have access to these interim results and make the final decision to terminate the trial	
Harms	22	Plans for collecting, assessing, reporting, and managing solicited and spontaneously reported adverse events and other unintended effects of trial interventions or trial conduct	Page 11
Auditing	23	Frequency and procedures for auditing trial conduct, if any, and whether the process will be independent from investigators and the sponsor	
**Ethics and dissemination**			
Research ethics approval	24	Plans for seeking research ethics committee/institutional review board (REC/IRB) approval	Page 14
Protocol amendments	25	Plans for communicating important protocol modifications (eg, changes to eligibility criteria, outcomes, analyses) to relevant parties (eg, investigators, REC/IRBs, trial participants, trial registries, journals, regulators)	Page 10-11
Consent or assent	26a	Who will obtain informed consent or assent from potential trial participants or authorised surrogates, and how (see Item 32)	Page 6
	26b	Additional consent provisions for collection and use of participant data and biological specimens in ancillary studies, if applicable	
Confidentiality	27	How personal information about potential and enrolled participants will be collected, shared, and maintained in order to protect confidentiality before, during, and after the trial	Page 14
Declaration of interests	28	Financial and other competing interests for principal investigators for the overall trial and each study site	Page 14
Access to data	29	Statement of who will have access to the final trial dataset, and disclosure of contractual agreements that limit such access for investigators	Page 14
Ancillary and post-trial care	30	Provisions, if any, for ancillary and post-trial care, and for compensation to those who suffer harm from trial participation	

For peer review only

Dissemination policy	31a	Plans for investigators and sponsor to communicate trial results to participants, healthcare professionals, the public, and other relevant groups (eg, via publication, reporting in results databases, or other data sharing arrangements), including any publication restrictions	Page 14
	31b	Authorship eligibility guidelines and any intended use of professional writers	Page 14
	31c	Plans, if any, for granting public access to the full protocol, participant-level dataset, and statistical code	
**Appendices**			
Informed consent materials	32	Model consent form and other related documentation given to participants and authorised surrogates	
Biological specimens	33	Plans for collection, laboratory evaluation, and storage of biological specimens for genetic or molecular analysis in the current trial and for future use in ancillary studies, if applicable	

*It is strongly recommended that this checklist be read in conjunction with the SPIRIT 2013 Explanation & Elaboration for important clarification on the items. Amendments to the protocol should be tracked and dated. The SPIRIT checklist is copyrighted by the SPIRIT Group under the Creative Commons "Attribution-NonCommercial-NoDerivs 3.0 Unported" license.

For peer review only

Impact of Pulse Oximetry on Hospital Referral Acceptance in Children under 5 with Severe Pneumonia in Rural Pakistan (District Jamshoro): Protocol for a Cluster Randomized Trial

Journal:	*BMJ Open*
Manuscript ID	bmjopen-2020-046158.R1
Article Type:	Protocol
Date Submitted by the Author:	08-Apr-2021

Complete List of Authors:	Mir, Fatima; Aga Khan University, Pediatrics and Child Health; Aga Khan University, Aga Khan University<br>Ali Nathwani, Apsara; Aga Khan University,<br>chanar, suhail; Aga Khan University, Paediatrics and child health<br>Hussain, Amjad; Aga Khan University, Pediatrics and Child Health<br>Rizvi, Arjumand; Aga Khan Medical University, Pediatrics and Child Health<br>Ahmed, Imran; Aga Khan University<br>Memon, Zahid Ali; Aga Khan University, Centre of Excellence in Women and Child Health<br>Habib, Atif; Aga Khan University, Department of Paediatrics & Child Health<br>Soofi, Sajid; Aga Khan University Hospital, Paediatric & Child Health<br>Bhutta, Zulfiqar; Aga Khan University, Division of Women and Child Health

**Primary Subject Heading**:	Global health
Secondary Subject Heading:	Health services research, Infectious diseases, Paediatrics, Public health
Keywords:	Public health < INFECTIOUS DISEASES, Paediatric infectious disease & immunisation < PAEDIATRICS, PUBLIC HEALTH, RESPIRATORY MEDICINE (see Thoracic Medicine), Clinical trials < THERAPEUTICS

SCHOLARONE™ Manuscripts

I, the Submitting Author has the right to grant and does grant on behalf of all authors of the Work (as defined in the below author licence), an exclusive licence and/or a non-exclusive licence for contributions from authors who are: i) UK Crown employees; ii) where BMJ has agreed a CC-BY licence shall apply, and/or iii) in accordance with the terms applicable for US Federal Government officers or employees acting as part of their official duties; on a worldwide, perpetual, irrevocable, royalty-free basis to BMJ Publishing Group Ltd ("BMJ") its licensees and where the relevant Journal is co-owned by BMJ to the co-owners of the Journal, to publish the Work in this journal and any other BMJ products and to exploit all rights, as set out in our licence.

The Submitting Author accepts and understands that any supply made under these terms is made by BMJ to the Submitting Author unless you are acting as an employee on behalf of your employer or a postgraduate student of an affiliated institution which is paying any applicable article publishing charge ("APC") for Open Access articles. Where the Submitting Author wishes to make the Work available on an Open Access basis (and intends to pay the relevant APC), the terms of reuse of such Open Access shall be governed by a Creative Commons licence – details of these licences and which Creative Commons licence will apply to this Work are set out in our licence referred to above.

Other than as permitted in any relevant BMJ Author's Self Archiving Policies, I confirm this Work has not been accepted for publication elsewhere, is not being considered for publication elsewhere and does not duplicate material already published. I confirm all authors consent to publication of this Work and authorise the granting of this licence.

For peer review only

Impact of Pulse Oximetry on Hospital Referral Acceptance in Children under 5 with Severe Pneumonia in Rural Pakistan (District Jamshoro): Protocol for a Cluster Randomized Trial

Fatima Mir[1]*, Apsara Ali Nathwani[2], Suhail Chanar[2], Amjad Hussain[2], Arjumand Rizvi[2], Imran Ahmed[2], Zahid Memon[2], Muhammad Atif Habib[2], Sajid Soofi[1,2], Zulfiqar A Bhutta[2]

Affiliations

[1]Department of Pediatrics and Child Health, The Aga Khan University, Pakistan

[2]Centre of Excellence in Women & Child Health, The Aga Khan University, Pakistan.

***Corresponding author**

Dr. Fatima Mir

Associate Professor

Department of Pediatrics and Child Health, the Aga Khan University

Stadium Road, Karachi 74800

Pakistan

Email: fatima.mir@aku.edu

Phone: +92 3005844610

Key words: Severe pneumonia, hypoxemia, pulse-oximetry, Lady Health Workers (LHWs), community case management pneumonia, hospital referral.

For peer review only

Abstract

Background: Pneumonia is a leading cause of death among children under five specifically in South Asia and Sub-Saharan Africa. Hypoxemia is a life-threatening complication among children under five with pneumonia. Hypoxemia increases risk of mortality by 4.3 times in children with pneumonia than those without hypoxemia. Prevalence of hypoxemia varies with geography, altitude and severity (9-39% Asia, 3-10% African countries). In this protocol paper we describe research methods for assessing impact of lady health workers identifying hypoxemia in children with signs of pneumonia during household visits on acceptance of hospital referral in District Jamshoro, Sindh.

Methods and Analysis: A cluster randomized controlled trial using pulse oximetry as intervention for children with severe pneumonia will be conducted in community settings. Children aged 0-59 months with signs of severe pneumonia will be recruited by Lady Health Workers (LHWs) during routine visits in both intervention and control arms after consent. Severe pneumonia will be defined as fast breathing and/or chest in-drawing, and, one or more danger sign and/or hypoxemia (Sa02<92%) in PO (intervention) group and fast breathing and/or chest in-drawing and 1/more danger sign in clinical signs (control) group. Recruits in both groups will receive a stat dose of oral amoxicillin and referral to designated tertiary health facility. Analysis of variance will be used to compare baseline referral acceptance in both groups with that at end of study.

Ethics and Dissemination: Ethical approval was granted by the Ethics Review Committee of the Aga Khan University (4722-Ped-ERC-17), Karachi. Study results will be shared with relevant government and non-governmental organizations, presented at national and international research conferences and published in international peer-reviewed scientific journals.

Trial registration: Clinicaltrials.gov Registry NCT03588377. Trial registration Date: May 05, 2018. Last update Date: July 31 2020. Study Status: ongoing

For peer review only - http://bmjopen.bmj.com/site/about/guidelines.xhtml

Article Summary

Strengths and Limitations of this study

- Our study will directly assess impact of pulse oximetry on family referral acceptance by comparing family acceptance of hospital referral (verbal acceptance with hospital visit) in ‘pulse oximetry and clinical signs’ group with ‘clinical signs alone’ group
- The study has applicability because it assesses impact with ‘real-life’ limitations (variability in lady health worker accuracy in identifying severe pneumonia, availability of oxygen and human resource at referral hospital and, availability of private transportation)
- The study design provides means of minimizing the effect of confounding
- The study design avoids bias in allocation to exposure groups
- Blinding is not possible due to nature of intervention
- Some subjects may fail to adhere to protocol and non-adherence may cause an underestimated measure of association.

For peer review only

For peer review only - http://bmjopen.bmj.com/site/about/guidelines.xhtml

Introduction

Pneumonia accounts for an estimated 18% of under-5 mortality across the globe [1] Majority of these pneumonia-specific deaths occur in 15 countries, in which Pakistan ranks fifth. [2] Failure to seek early care and delays in hospital referral are commonly acknowledged determinants of mortality in childhood pneumonia with a higher proportion reported from rural settings than urban. [3-6] Acceptance rates of 'facilitated' hospital referral advice have been reported low between 8% and 23% for sick young infants in peri-urban Karachi. [7, 8] They are even lower for non-facilitated referral in rural settings in children under 5 with severe pneumonia in rural Matiari district, Sindh. [5] The prominent reasons in developing countries behind this delay are inability to recognize seriousness of pneumonia, distance from health facility and lack of money for private health care. [9, 10]

In 1994, the Government of Pakistan introduced the Lady Health Worker (LHW) Program in rural populations with low physician density to address common health problems in women and children under 5 through household visits. To date, a team of over 110,000 LHWs are working for the program nationwide with 23,185 LHWs in Sindh alone [11]. Each LHW is responsible for a population of 1000–1500 individuals (catchment of about 100 families). Recruits are preferably local, with a minimum of 8 years of formal schooling followed by 15 months of training to deliver maternal and child health (MCH) care in community settings. During a monthly home visit, the LHW provide essential maternal and child health care services including family planning needs, nutritional assessments of both mother and child, management of minor and common illnesses, improving immunization coverage and imparting health education. [12]

This study recognizes the LHW as a powerful conduit for reaching under 5 children at household level and identifying severe pneumonia in under five at an early stage. It also recognizes the underestimation of hypoxemia (SpO_2 of <90%), a major risk factor for pneumonia mortality at community level and assesses its prevalence. The reported prevalence of hypoxemia in under 5 acutely ill children is 5 to 58% in facilities [13-16] and 16 to 39% in community settings. [17] A 4.3 times higher risk of mortality has been associated with pneumonia with hypoxemia than in children with pneumonia without hypoxemia. [13] Hypoxemia is also predictive of treatment failure with amoxicillin in 3-59 months old children. [18] In resource poor settings where pulse oximetry is not feasible, signs and symptoms of severe pneumonia (sleepiness, cyanosis, head

For peer review only - http://bmjopen.bmj.com/site/about/guidelines.xhtml

nodding/grunting, and inability to move) are used as predictors of hypoxemia. However, the validity of clinical signs to predict hypoxemia varies and it is often difficult for physicians working in settings, where objective detection of hypoxemia is not available, to decide whether the child coming with severe pneumonia requires administration of oxygen or not. [15, 19, 20]

Pulse oximetry [16] is a rapid, portable, non-invasive and accurate method of measuring arterial hemoglobin oxygenation (Sp02) and has therefore been used in trial and clinical settings to detect hypoxemia. Appropriate oxygen therapy (based on PO findings rather than clinical signs of severity alone) has been associated with lower mortality risk. [19] Assuming access to supplemental oxygen, PO could potentially avert up to 148,000 severe pneumonia related deaths if implemented, and, combining PO with IMCI assessment for pneumonia has been shown to be cost effective in 15 high burden countries. [21] Emdin et al found first level Lady Health Workers in peri-urban Karachi could easily perform pulse oximetry on young infants on well and sick visits to a primary health care facility. [22]

Over the past decade, the possible impact of pulse oximetry in hospital and community settings has been of interest across the globe. Health survey of 54 countries in 2010, suggested that 19.2% of the operating theatres around the globe are not equipped with pulse oximeters [23]. Trials assessing utility of pulse oximetry on a health systems level in Nigeria have shown that health workers reserve PO for the sickest patients [24, 25]. This has also been observed in areas at higher altitudes with higher prevalence of hypoxemia (highlands of Papua New Guinea) [25, 26]. There is lack of clarity about how pulse oximetry can be used in the community where lower hypoxemia prevalence may be a lesser incentive for health workers to use PO such as in interior Sindh settings in Pakistan. Then again, health workers recruited from within communities may be better invested and motivated than hospital personnel in following case management guidelines precluding PO. It is therefore worthwhile to explore and describe contexts behind a family's acceptance (or not) of referral advice whether based on technology and/or clinical examination in rural settings where the highest burden of pneumonia deaths lies.

Feasibility and sustainability audits of oxygen delivery systems in the Gambia and Egypt have shown that providing technology alone is ineffective, and should preclude provision of supplies, education, training and feedback [27-30]. This protocol paper describes a study to assess the

For peer review only - http://bmjopen.bmj.com/site/about/guidelines.xhtml

effect of PO monitoring in community settings on hospital referral acceptance in children under 5 with severe pneumonia.

Methods

Study aims and design:

The overall aim of the study is to assess if detection of hypoxemia and/or severe pneumonia in children 0-59 months by LHWs during their monthly home visits will increase hospital referral acceptance among families in District Jamshoro, Sindh, Pakistan.

Specific objectives are:

1) To assess and compare the impact of 'pulse oximetry' used by LHWs at household level on increasing hospital referral acceptance rates in intervention clusters (district Jamshoro) for 0-59 months old children with severe pneumonia with the impact of LHWs using clinical signs alone in non-intervention clusters of the same district
2) To determine prevalence of severe pneumonia ± hypoxemia in 0-59-month olds
3) To investigate the likely predictors (demographic, clinical) of hospital referral acceptance in both the groups
4) To compare clinical outcomes (treatment completion, treatment failure, hypoxemia) of children 0-59 months who accepted referral to those who refused admission and were treated at home

A community-based cluster randomized trial will be conducted in district Jamshoro, among children of ages 0-59 months **(Figure 1)**. In intervention areas, all children with cough, fever or difficulty in breathing (acute respiratory illness) will be assessed for study eligibility/referral criteria (signs and symptoms of severe pneumonia, OR hypoxemia alone, OR severe pneumonia with hypoxemia) by LHWs during their monthly home visit in their catchment area. In control areas, study eligibility will require detection of signs of severe pneumonia alone (Appendix 1) during LHW monthly visits. Data will be collected on demographics, likely predictors, and clinical outcomes using a structured questionnaire.

The primary outcome is hospital referral acceptance in children under 5 with severe pneumonia and to identify demographic and clinical predictors of hospital referral acceptance. The

For peer review only - http://bmjopen.bmj.com/site/about/guidelines.xhtml

predictors will include distance of child home to referral facility, socio-economic status of household, parental education, child age, nutritional status, respiratory rate, temperature, hypoxemia and presence of other illnesses.

Secondary outcomes include duration of oxygen therapy, treatment failure, duration of hospital stay, vital and health status of child at day 7th and 14th.

Trial setting:

The study will be conducted within the community of Taluka Kotri in District Jamshoro, Sindh **(Figure 2)**. Jamshoro District has a population of 993,142 [31]. It is predominantly rural, with 33% literacy, and >50% employed daily wage laborers. A baseline survey conducted as a part of current study showed the status of overall health indicators: skilled birth attendance 57%, antenatal care coverage 75%, postnatal care cover for mother and newborn within 48 hours 31.5%, vaccination completeness in children 12-23 months 68% and care seeking for ARI and diarrhea >80% (internal survey). A total of 27 health facilities function in the district including one District Headquarter (DHQ) Hospital, three Taluka Headquarter (THQ) Hospitals, five Rural Health Centers (RHCs) and 18 Basic Health Units (BHUs). The district is divided administratively in 30 union councils. Kotri is one of the 4 Talukas of Jamshoro, consists of 44% (437,561) of the population of district [31].

Participants will be the permanent residents of Kotri, Jamshoro and recruited from their homes during routine monthly visits by Lady Health Workers serving in their catchment areas. This study is expected to run for 48 months with participant identification and enrolment conducted simultaneously in intervention and control clusters over 21 months after an initial pilot of one month. Each enrolled child will be revisited at day 7 and 14 for outcome measurement.

Participant:

Any child aged 0-59 months having signs and symptoms of acute respiratory illness (cough, fever, difficulty in breathing) in intervention clusters will undergo assessment of 1) signs and symptoms of severe pneumonia and, 2) pulse oximetry during monthly LHW home visits. Presence of severe pneumonia with or without hypoxemia, or hypoxemia alone will merit hospital referral (non-facilitated). Any child aged 0-59 months having signs and symptoms of acute respiratory illness (cough, fever, difficulty in breathing) in control clusters will undergo

For peer review only - http://bmjopen.bmj.com/site/about/guidelines.xhtml

assessment of 1) signs and symptoms of severe pneumonia alone. Presence of severe pneumonia will merit hospital referral (non-facilitated). Exclusion criteria will include lack of consent and, non-availability (lost to follow up, migration) on days of scheduled follow-up visits (days 7 and 14). Those who accepted referral but did not show up at hospital will be included in final analysis **(Appendix 1).**

Randomization and masking:

Study clusters were defined as the area covered by an LHW. Each LHW covers a minimum of 100 households. A list of LHWs working in Kotri was collected from the LHW program, Health Department Government of Sindh. There are a total of 188 active LHWs in the study site. A baseline survey was conducted to collect data on health indicators from the LHW catchments. The clusters were randomly allocated to intervention and control groups on 1:1 fashion with a computer-generated randomization sequence that was generated by an independent expert. Clusters were matched on under-5 population and distance to referral health facility. No stratification was used for allocation; clusters were selected to ensure that the reporting and training centres of intervention and control LHWs were separate. The investigators and the national and provincial LHW program coordinators will be excluded from the allocation process.

Training of Lady Health Workers:

The Lady Health Worker (LHW) program of Pakistan consists of a community based group of first-level health workers with the principal mandate of home-based Maternal and Child Health. The recruitment process is well defined and selection criteria include: at least 8 years of education with middle school pass, local residency, recommendation from the community, and preferably married [32]. Once selected, they receive 15 months of basic training in mid-wifery and family planning using standardized training manuals and curriculum, and periodic refresher training courses. Each of these LHWs is typically responsible for approximately 1000 people, or 150 homes, and often serve as the primary health care contact in these rural communities [32, 33].

For peer review only - http://bmjopen.bmj.com/site/about/guidelines.xhtml

We chose LHWs as study personnel due to their access to homes on regular monthly basis. We trained intervention and control LHWs in separate groups for all of the following: 1) classification of ARI (no pneumonia, pneumonia, severe pneumonia) using standard acute respiratory infections training modules (WHO and IMNCI) 2) identification of danger signs 3) case management of pneumonia at home with oral amoxicillin and severe pneumonia with stat dose of antibiotic before hospital referral. Intervention LHWs received an additional training in using a pulse oximeter and obtaining a valid reading. The Principal Investigator led these training sessions with senior trainers of the LHW program (Lady Health Supervisers).

Participant Recruitment and Study Procedures:

Intervention delivery

Children aged 0–59 months with cough and/or difficult breathing during regular home visits of LHWs will be assessed for firstly, signs and symptoms of severe pneumonia (fast breathing/chest in-drawing and one or more danger sign (unable to eat/drink, vomiting, convulsion and lethargy/unconsciousness) and/or Stridor)) and secondly, hypoxemia (SpO_2 <92%) using a handheld pulse oximeter (Masimo Rad-5v) to measure blood oxygen saturation level. LHWs will also do case management of children with pneumonia and severe pneumonia. A 3-day course of oral amoxicillin will be given to children with pneumonia at home, whereas children meeting referral criteria (severe pneumonia alone, hypoxemia alone, or severe pneumonia and hypoxemia) after obtaining informed consent, will be administered a stat dose of oral amoxicillin and referred to nearest referral hospital (DHQ Kotri).

The study investigators will have provided these pulse oximeters to the LHW Program in advance and highlighted which ones will receive them. Physicians at the referral center serving the intervention clusters will also receive handheld pulse oximeters. All the LHWs and staff will be trained on the use, and maintenance of these pulse oximeters. Children with severe pneumonia with or without hypoxemia will be advised to go to hospital for antibiotics and oxygen, using the PO reading as a tool to convince parents. Children with hypoxemia alone, without signs of severe pneumonia will be referred to hospital to rule out cyanotic congenital heart disease. Name of the pre-designated health facility with available oxygen and study physician will be provided to all the LHWs so that Study Workers (non-LHW study personnel) can coordinate with Study Physicians and ensure the patient has reached and is receiving safe and recommended care at

For peer review only - http://bmjopen.bmj.com/site/about/guidelines.xhtml

referral facility. Project staff will pretest and regularly monitor PO accuracy and quality of readings.

Hypoxemia will be defined as an arterial oxygen saturation (SpO2) <92%. SpO2 measurement will be recorded after 1 minute of stable observation. If the SpO2 comes 92% or less, the child will first be assessed for nasal obstruction with readings repeated after applying nasal saline drops. If repeat reading shows hypoxemia, the child will be referred to nearest designated referral hospital and admitted for oxygen via nasal or nasopharyngeal route and intravenous antibiotics, as per recommendations.

Implementation of active control: Clinical Signs assessment

Children aged 0–59 months with cough and/or difficult breathing during regular home visit will be assessed by LHWs for signs and symptoms of severe pneumonia (fast breathing/chest in-drawing and one or more danger sign (unable to eat/drink, vomiting, convulsion and lethargy/unconsciousness) and/or Stridor)). A 3-day course of oral amoxicillin will be given to children with pneumonia at home, whereas children with severe pneumonia (eligible for recruitment) will be requested for informed consent and offered stat dose of oral amoxicillin and referral to nearest referral hospital.

Procedure at referral facility

Children who accept hospital referral in both intervention and control clusters, and reach hospital premises with LHW referral slip will be assessed by study physician at the referral center. An SMS notification with brief details of referred child will have been provided to trained study personnel (study physician) in advance at time of referral at both the referral facilities. Children with severe pneumonia and/or hypoxemia as per LHWs who reach referral hospital premises will be examined and subjected to pulse oximetry again by the study physician at referral facility. If signs and symptoms of severe pneumonia are present, the child will be admitted for further appropriate treatment (Oxygen therapy via nasal or nasopharyngeal route and intravenous antibiotics etc.) and if the symptoms are not severe (absence of danger sign), the child will be treated in outpatient care as per the standard of referral facility. All the children admitted at referral facility will undergo 12 hourly monitoring by study personnel and filling of case reporting form (CRF) and hospital physician form (HPF) at day 1, 7 and 14. Those children who

For peer review only - http://bmjopen.bmj.com/site/about/guidelines.xhtml

refused the referral will be visited by study community health workers after 24 hours to confirm referral refusal and to fill CRF.

Preliminary meetings will be held with the Executive Director Health Jamshoro, Director General Health Sindh, In-charge Lady Health Worker Program Sindh and In-charge Pediatric Units LUMHS to ensure their cooperation through study duration. Emergency and pediatric unit staff at the referral facilities along with study personnel (physician/nurse) will be trained on management of severe pneumonia according to the integrated management of neonatal and childhood illnesses (IMNCI) guidelines [34]. A baseline survey will be conducted at the health facilities to ensure availability of oxygen and necessary intravenous antibiotics. Even though it is ideal to guarantee sustainable oxygen systems at the two chosen referral public sector hospitals, this study does not provide oxygen and therefore aims to assess 'real-life' situations in public hospitals and their impact on severe pneumonia outcomes with or without hypoxemia. LHWs will be incentivized on basis of their contribution to the study activities.

For peer review only

Data Collection and Storage

Data will be collected by LHWs during house visits (Screening form), community health workers (during follow up visits day 1, 7 and 14) and hospital based study personnel (for all who accept referral and reach hospital premises) on paper forms **(Appendix 2)**. Given that it will be a new experience for LHWs to assess, classify and manage ARI cases and at the same time record findings on data forms accurately, these will be supervised closely and frequently, at least for the first pneumonia season. Well trained study field supervisory officers and LHW supervisors will be required to perform regular field supervision in their respective clusters and ensure accurate and logically entered data forms and make necessary verifications and corrections at the data collection sites and give feedback to the LHW to avoid repeating the errors. Raw data brought to the program office will be checked once again for accuracy by the technical staff and approved for entry in the computer. All raw data will be safely kept in the AKU office, appropriately numbered by cluster, until seven years after the study is over.

Case History Records

These include the study case report forms (CRF) and hospital physician form (HPF) that will contain information that documents the child's eligibility to participate in the study, the signed consent form, and information from tests and examinations. Wherever possible copies of

For peer review only - http://bmjopen.bmj.com/site/about/guidelines.xhtml

supporting documentation for the information contained in the CRF should be kept with each patient's case history record. This supporting documentation may include records of physical examinations, progress notes, laboratory reports, X-rays, consultations, correspondence, information and data on the subject's condition, during and after the clinical investigation, diagnoses made, concomitant therapy, etc. All information in the case history records should be attributable to a specific individual. Since the CRF will not contain the patient's name, there will be a unique link between the ID number on the CRF and the patient's name. Each child's case history record will be evaluated to verify validity and completeness of the data on the CRF when a study monitor visits the study site. All corrections to CRF's must be made without obscuring the original entry. The revised entry should be inserted and the person making the correction should sign and date the correction. Only authorized study personnel may complete or correct case report forms.

Data Management

Screening data will be collected on paper by LHWs. Case reporting form (CRF) and Hospital physician form will be collected on electronic forms. To ensure proper implementation of the intervention, the field supervisors will make spot checks and will arrange monthly refresher group sessions of the first-line health workers in which the problems encountered will be discussed and resolved. In addition, the data collection activity will be carried out by teams consisting of LHWs/CHWs and study staff will be further monitored by field supervisors who will perform a check on a subset (5%) of households.

An information system will be set up to keep track all patients screened and enrolled and a filing system to keep all study related records - case history records, study protocol or related documentation and drug distribution records. The coordinator at the site will be responsible for the completeness and accuracy of all the study materials.

Study Protocol and Related Documentation

All study related documents including the study protocol, manuals of operations, all correspondence sent to or received from the study monitor, materials used for obtaining informed consent, protocol modifications and records of the Institutional Review Board approval and all communications with the IRB must be maintained in complete form. These documents will be

For peer review only - http://bmjopen.bmj.com/site/about/guidelines.xhtml

evaluated to ensure that study documentation is complete and current when a study monitor visits the study site.

Record Retention

Retention of accurate and complete records is essential to establish the validity and completeness of the study. All records must be retained for 7 years after the data set is frozen. Electronic data will be de-identified, unlinked from any personal identifiers and therefore will protect individual identity.

Reporting of Serious Adverse Events and Treatment Failures

Amoxicillin is in widespread use and is not investigational in any study site. However, since oral amoxicillin is not routinely recommended for initial treatment of children who have severe pneumonia, the appropriate case report forms describing the occurrence of a serious adverse event, treatment failure or death must be faxed to the coordinating center within 72 hours of the site coordinator knowing about the event. Adverse events, treatment failure and death must be reported to allow appropriate interpretation of this critical information. If the outcome of the adverse event is unknown when the site coordinator first notifies the coordinating center, a follow-up form must be faxed to the coordinating center within 10 days of knowing about the event. The PI should send a copy of the adverse event data to their local IRB as soon as possible. The coordinating center will summarize the Adverse Event and Death information and send a report to the IRB/ERC of sponsors and to site IRBs. Both the rate of adverse events and the rate of patient accrual at each individual site will be monitored to determine if stopping rules are met. We do not anticipate serious adverse events. However, in case of one, a DSMB will be requested for and convened on ad hoc basis for safety review at any time during the study if there is a concern regarding rates of adverse events or rates of patient accrual. Adverse events will be reported by the study physician to the principal investigator and clinically managed by the study physician in conjunction with other physicians at the institution. Any related and unexpected life-threatening adverse event including death will be reported to the IRB within 2 business days as per IRB protocol and any related, unexpected and serious adverse event will be reported to the IRB within 10 business days as per IRB protocol.

For peer review only - http://bmjopen.bmj.com/site/about/guidelines.xhtml

Compliance with and Deviations from the Study Protocol

The Site coordinator must agree with and sign the protocol and confirm in writing that he or she has read, understands and will work according to the protocol and Good Clinical Practice. The Site coordinator is responsible for making sure that the protocol is strictly followed and should not make any changes to the study unless necessary to eliminate an apparent immediate hazard or damage to a trial subject. Any deviations from the study protocol including but not limited to inappropriate enrollment of a study subject, administration of the wrong study treatment, missed doses of study treatment, missed observation points, incorrect administration of concomitant medications, etc. should be reported to the coordinating center and each site's IRB. The report should include a plan to rectify any problems at the site that may have caused the protocol deviation.

Sample size:

Defining a cluster as (the catchment area of) one Lady Health Worker (LHW), and assuming a power of 90% in detecting 50% increase in referral acceptance from a 10 % baseline to 15% among 2-59 month old children with severe pneumonia (pneumonia prevalence at 2 week recall (MICS Sindh):7.5% (18% of which is assumed severe Pneumonia) with ICC 0.001736. We need to capture a total of 4160 children with severe pneumonia in both intervention and control groups.

Data Analysis:

The primary analysis for each outcome will perform on an 'intention-to-treat' (ITT) basis, i.e. all children included in the analysis who were enrolled in the study according to the group to which they were allocated. All analyses will account for the cluster-randomized design to ensure correct type I error rates and confidence intervals [35]. Baseline characteristics will be compared by analyzing differences in means and proportions among the study arms. Categorical outcomes will be compared using chi square test and continuous outcomes using Student's t-test. For analysis of predictors of referral acceptance, generalized linear model will be used with logit link function. The univariate analysis will be conducted to explore the independent effect of each predictor on outcome. The variables significant at a liberal p-value of <0.20 will be included in multivariate model for adjustment. The results will be reported as relative risk (RR) with 95% CI. Type 1 error will be set at 5% level. All analysis will be done using STATA version 15.

For peer review only - http://bmjopen.bmj.com/site/about/guidelines.xhtml

Patient and Public Involvement

Patients or the public will not be involved in the design or conduct of the study. Results will be disseminated to the community.

Study Status

Recruitment began in August 2019 and field activities and data collection are in process with end date Dec 31, 2021. As of 16th October 2020, a total of 235 cases and 184 controls have been enrolled. Extension of study duration is in discussion to achieve sample size.

Discussion

Hypoxemia, a frequent complication of severe pneumonia, is a major risk factor for death in children under 5. Theoretically, detection of hypoxemia at community level among severe pneumonia cases by the use of pulse-oximeter would give awareness to the caregivers about severity of illness and reduce delay in hospital referral. Delayed care-seeking is a recognized risk factor in pneumonia mortality in community settings [36]. Unfortunately, information on prevalence of hypoxemia, effectiveness of its detection in influencing parents to seek hospital care (gold standard for severe pneumonia), and impact of appropriate care at hospital in settings like Pakistan is lacking. Thus, the findings of this study will build evidence for utility of providing front-line workers like LHWs with a tool to detect hypoxemia if signs and symptoms of pneumonia are present. Interestingly various groups across the world are now espousing pulse-oximetry with other strengths like detection of congenital heart disease [37, 38] and newborn sepsis [39].

Limitations

Some union councils with poor LHW coverage were not included in this study. This was a compromise on generalizability in favour of feasibility. It may have led to exclusion of children whose referral patterns were important to gauge. We also did not offer facilitated referral in either arm. This may have affected referral acceptance in both groups however will allow assessment of real-life impact of transportation barriers.

Though we kept hospital referral (as recommended by WHO) for severe pneumonia as our standard of care, it was beyond the scope of this study to ensure that referral centres had sustainable oxygen systems/capacity for non-invasive ventilation in children with severe

For peer review only - http://bmjopen.bmj.com/site/about/guidelines.xhtml

pneumonia, or readily available cardiac evaluation resources for children with hypoxemia alone. Duke et al showed improved case fatality rates by providing oxygen concentrators and pulse-oximeters at five hospitals in PNG along with protocols for use [35]. Lack of quality care at referral hospitals in developing countries is a recognized barrier to health care seeking behavior[40, 41].

If our study reveals pulse-oximetry has influenced health-seeking behavior significantly, we will need to follow with a more systematic evaluation of pneumonia care at rural hospitals which vary in quality of care. We will also need to correlate recovery rates in those who accepted hospital referral versus those who stayed home on oral amoxicillin. Evidence to support home care for severe pneumonia is poor [42].

The COVID pandemic has adversely affected recruitment in the study with disruption of field activities for many months in 2020. We anticipate prolongation of study duration in order to achieve sample size.

Ethics and Dissemination

This study has been approved by the Ethical Review Committee of The Aga Khan University (4722-Ped-ERC-17), Karachi, Pakistan in June 2017. Written informed consent in the local language will be obtained from parents or guardians of all participants. Data forms will contain no identifying information other than age, sex and GIS coordinates. Laboratory forms will contain no identifying information, specimens will be identified by a study number only and test results will not be linked to any individual by name. All survey staff will sign a confidentiality agreement to ensure that they do not release participant identities and test or study results to individuals who are not part of the study team.

Study progress and findings will be shared with sponsors (BMGF) quarterly. Results will be presented at national and international research meetings and conferences and also prepared for publication in international peer-reviewed scientific journals. Study findings will be disseminated to the study communities.

For peer review only - http://bmjopen.bmj.com/site/about/guidelines.xhtml

Declarations:

Consent for publication: Yes

Availability of data and materials: The datasets used for the article and the study is available from the corresponding author on request.

Competing interests: The authors declare that they have no competing interests.

Funding: The study is funded by Bill & Melinda Gates Foundation through Grant OPP1148892.

Author Contributions: FM, ZM, MAH, SBS & ZAB: conceptualization of project. FM, AAN, and SC: development of study design and questionnaires. AAN and SC: oversight of data collection. AAN, SC and AH: support of study logistics and field activities. IA and AR: statistical analyses. SBS and ZAB: overall supervision and critical input. All authors have read and approved the final manuscript.

Acknowledgements: We would like to acknowledge the mothers and families who have contributed to the study. We are grateful to the Sindh LHW Program, Department of Health for their support and facilitation of the trial.

For peer review only

For peer review only - http://bmjopen.bmj.com/site/about/guidelines.xhtml

References

1. Liu, L., et al., *Global, regional, and national causes of child mortality: an updated systematic analysis for 2010 with time trends since 2000.* Lancet, 2012. **379**(9832): p. 2151-61.
2. Rudan, I., et al., *Epidemiology and etiology of childhood pneumonia.* Bull World Health Organ, 2008. **86**(5): p. 408-16.
3. *WHO/UNICEF Joint Statement: Management of Pneumonia in community settings.* 2002.
4. *Ending Preventable Child Deaths from Pneumonia and Diarrhoea by 2025: The integrated Global Action Plan for Pneumonia and Diarrhoea (GAPPD).* 2013, World Health Organization: Geneva, Switzerland.
5. Soofi, S., et al., *Effectiveness of community case management of severe pneumonia with oral amoxicillin in children aged 2-59 months in Matiari district, rural Pakistan: a cluster-randomised controlled trial.* Lancet, 2012. **379**(9817): p. 729-37.
6. Kallander, K., D.H. Burgess, and S.A. Qazi, *Early identification and treatment of pneumonia: a call to action.* Lancet Glob Health, 2015. **4**(1): p. e12-3.
7. Owais, A., et al., *Why do families of sick newborns accept hospital care? A community-based cohort study in Karachi, Pakistan.* J Perinatol, 2011. **31**(9): p. 586-92.
8. Zaidi, A.K., et al., *Community-based treatment of serious bacterial infections in newborns and young infants: a randomized controlled trial assessing three antibiotic regimens.* Pediatr Infect Dis J, 2012. **31**(7): p. 667-72.
9. Ferdous, F., et al., *Mothers' perception and healthcare seeking behavior of pneumonia children in rural bangladesh.* ISRN Family Med, 2014: p. 690315.
10. Waiswa, P., et al., *Using the three delays model to understand why newborn babies die in eastern Uganda.* Trop Med Int Health, 2010. **15**(8): p. 964-72.
11. *LADY HEALTH WORKER PROGRAM FOR FAMILY PLANNING AND PRIMARY HEALTH CARE.* Available from: https://www.sindhhealth.gov.pk/LHW-Program.
12. Afsar, H., et al., *Factors affecting unsuccessful referral by the lady health workers in Karachi, Pakistan.* Journal-Pakistan Medical Association, 2003. **53**(11): p. 521-528.
13. Onyango, F.E., et al., *Hypoxaemia in young Kenyan children with acute lower respiratory infection.* BMJ, 1993. **306**(6878): p. 612-5.
14. Smyth, A., H. Carty, and C.A. Hart, *Clinical predictors of hypoxaemia in children with pneumonia.* Ann Trop Paediatr, 1998. **18**(1): p. 31-40.
15. Usen, S., et al., *Clinical predictors of hypoxaemia in Gambian children with acute lower respiratory tract infection: prospective cohort study.* BMJ, 1999. **318**(7176): p. 86-91.
16. McCollum, E.D., et al., *Multicenter study of hypoxemia prevalence and quality of oxygen treatment for hospitalized Malawian children.* Trans R Soc Trop Med Hyg, 2013. **107**(5): p. 285-92.
17. Basnet, S., R.K. Adhikari, and C.K. Gurung, *Hypoxemia in children with pneumonia and its clinical predictors.* Indian J Pediatr, 2006. **73**(9): p. 777-81.
18. Fu, L.Y., et al., *Brief hospitalization and pulse oximetry for predicting amoxicillin treatment failure in children with severe pneumonia.* Pediatrics, 2006. **118**(6): p. e1822-30.
19. Duke, T., et al., *Hypoxaemia in acute respiratory and non-respiratory illnesses in neonates and children in a developing country.* Arch Dis Child, 2002. **86**(2): p. 108-12.
20. Weber, M.W., et al., *Predictors of hypoxaemia in hospital admissions with acute lower respiratory tract infection in a developing country.* Arch Dis Child, 1997. **76**(4): p. 310-4.
21. Floyd, J., et al., *Evaluating the impact of pulse oximetry on childhood pneumonia mortality in resource-poor settings.* Nature, 2015. **528**(7580): p. S53-9.

For peer review only - http://bmjopen.bmj.com/site/about/guidelines.xhtml

22. Emdin, C.A., et al., *Utility and feasibility of integrating pulse oximetry into the routine assessment of young infants at primary care clinics in Karachi, Pakistan: a cross-sectional study.* BMC Pediatr, 2015. **15**: p. 141.
23. Funk, L.M., et al., *Global operating theatre distribution and pulse oximetry supply: an estimation from reported data.* The Lancet, 2010. **376**(9746): p. 1055-1061.
24. Ingram, G. and N. Munro, *The use (or otherwise) of pulse oximetry in general practice*. 2005, British Journal of General Practice.
25. Mokuolu, O.A. and O.A. Ajayi, *Use of an oxygen concentrator in a Nigerian neonatal unit: economic implications and reliability.* Ann Trop Paediatr, 2002. **22**(3): p. 209-12.
26. Matai, S., et al., *Implementing an oxygen programme in hospitals in Papua New Guinea.* Ann Trop Paediatr, 2008. **28**(1): p. 71-8.
27. Dobson, M., D. Peel, and N. Khallaf, *Field trial of oxygen concentrators in upper Egypt.* Lancet, 1996. **347**(9015): p. 1597-9.
28. Enarson, P., et al., *Implementation of an oxygen concentrator system in district hospital paediatric wards throughout Malawi.* Bull World Health Organ, 2008. **86**(5): p. 344-8.
29. Hill, S.E., et al., *Oxygen for treatment of severe pneumonia in The Gambia, West Africa: a situational analysis.* Int J Tuberc Lung Dis, 2009. **13**(5): p. 587-93.
30. Howie, S.R., et al., *Meeting oxygen needs in Africa: an options analysis from the Gambia.* Bull World Health Organ, 2009. **87**(10): p. 763-71.
31. Pakistan, P.B.o.S.G.o. *PROVINCE WISE PROVISIONAL RESULTS OF CENSUS - 2017*. 2017; Available from: http://www.pbs.gov.pk/content/block-wise-provisional-summary-results-6th-population-housing-census-2017-january-03-2018.
32. Zhu, N., et al., *Lady health workers in Pakistan: improving access to health care for rural women and families.* Boston: Harvard School of Public Health, 2014.
33. Organization, W.H., *Global health workforce alliance. Pakistan's lady health worker Programme: Global Health workforce alliance.* World Health Organization, Case study. Geneva: World Health Organization, 2008.
34. *Integrated Management of Childhood Illness (IMCI) (revised)*. 2014, Geneva: World Health Organization/The United Nation Children's Fund (UNICEF).
35. Duke, T., et al., *Improved oxygen systems for childhood pneumonia: a multihospital effectiveness study in Papua New Guinea.* The Lancet, 2008. **372**(9646): p. 1328-1333.
36. Ferdous, F., et al., *Pneumonia mortality and healthcare utilization in young children in rural Bangladesh: a prospective verbal autopsy study.* Tropical medicine and health, 2018. **46**(1): p. 1-10.
37. Engel, M.S. and L.K. Kochilas, *Pulse oximetry screening: a review of diagnosing critical congenital heart disease in newborns.* Medical Devices (Auckland, NZ), 2016. **9**: p. 199.
38. Tekleab, A.M. and Y.C. Sewnet, *Role of pulse oximetry in detecting critical congenital heart disease among newborns delivered at a high altitude setting in Ethiopia.* Pediatric Health, Medicine and Therapeutics, 2019. **10**: p. 83.
39. Jawin, V., et al., *Beyond critical congenital heart disease: newborn screening using pulse oximetry for neonatal sepsis and respiratory diseases in a middle-income country.* PLoS One, 2015. **10**(9): p. e0137580.
40. Latunji, O. and O. Akinyemi, *Factors influencing health-seeking behaviour among civil servants in Ibadan, Nigeria.* Annals of Ibadan postgraduate medicine, 2018. **16**(1): p. 52-60.
41. Musoke, D., et al., *Health seeking behaviour and challenges in utilising health facilities in Wakiso district, Uganda.* African health sciences, 2014. **14**(4): p. 1046-1055.

For peer review only - http://bmjopen.bmj.com/site/about/guidelines.xhtml

42. Das, R.R. and M. Singh, *Treatment of severe community-acquired pneumonia with oral amoxicillin in under-five children in developing country: a systematic review.* PloS one, 2013. **8**(6): p. e66232.

For peer review only

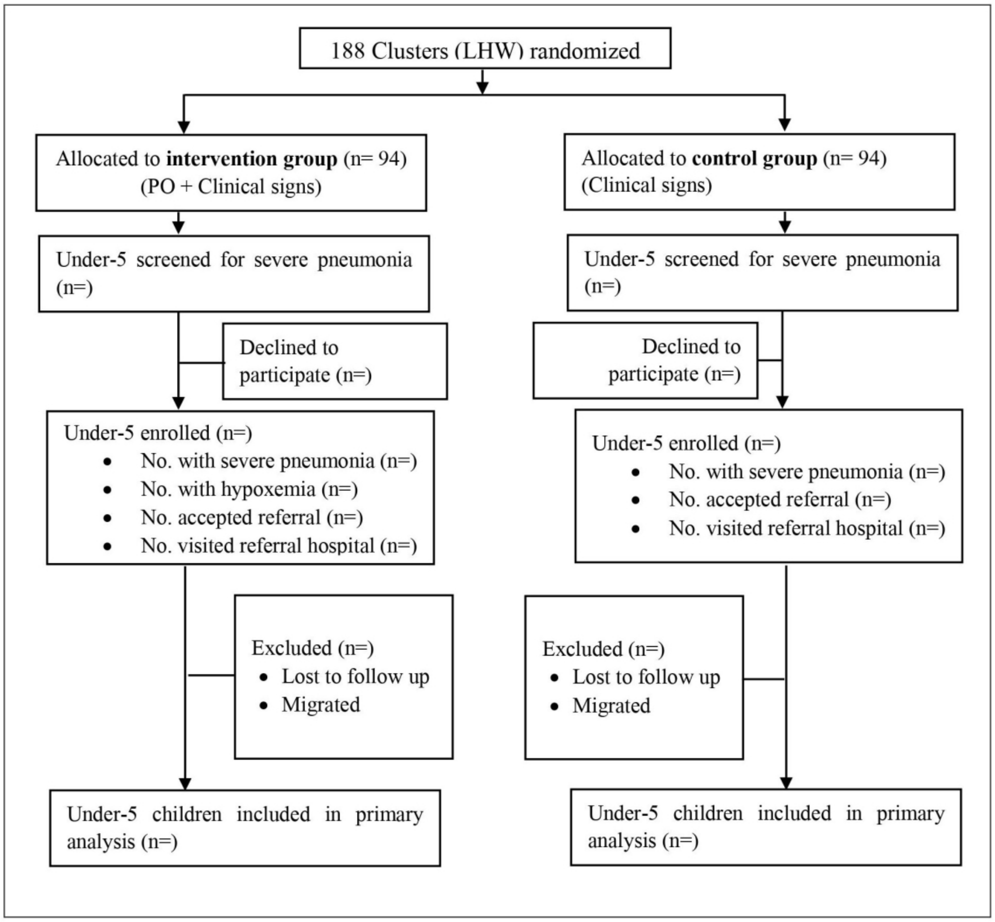

Trial Profile

514x481mm (96 x 96 DPI)

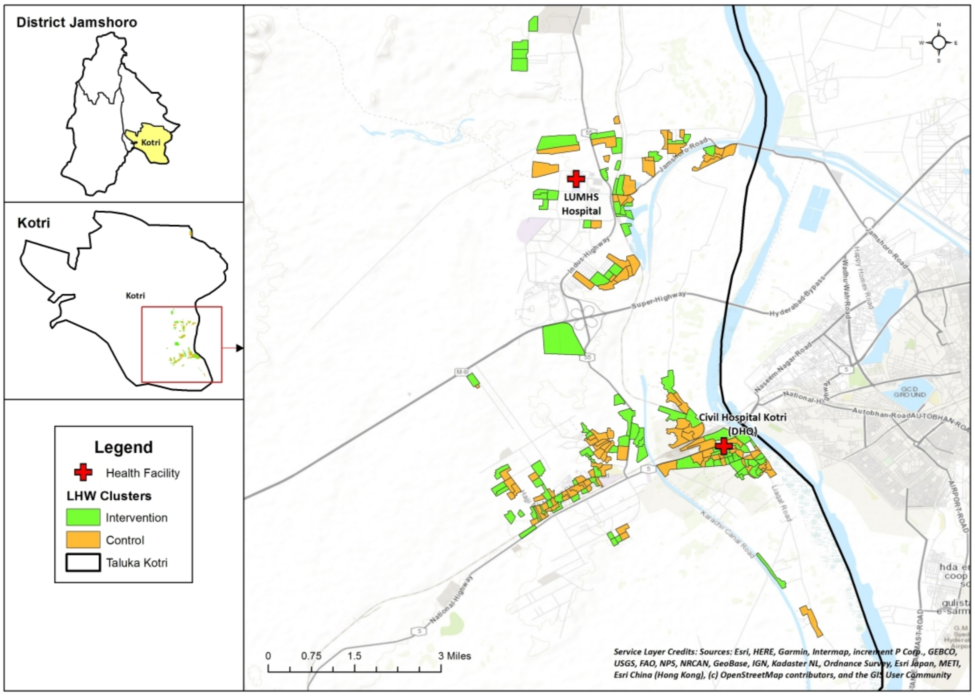

Study Intervention and Control Sites

296x210mm (300 x 300 DPI)

Appendix 1: Eligibility/Hospital Referral Criteria

**Hospital Referral Criteria**	
**Intervention Group**	**Control Group**
**Severe pneumonia AND/OR Hypoxemia**<br><br>**Children of 0 – 6 days:**<br>**1.** 1. Fast Breathing (≥ 60 breaths/min)<br>**AND/OR**<br>***2. Hypoxemia (<92 SpO2 Blood Oxygen Level)***<br><br>**Children of 7 days – 59 months:**<br>**1.** Fast Breathing and/or Chest In-drawing<br>➢ 0-2months: ≥ 60 breaths/min<br>➢ 2-12months: ≥50 breaths/min<br>➢ 12-59months ≥40 breaths/min<br>**AND**<br>**2.** Any ONE General Danger Sign* and/or Stridor<br>➢ *Unable to drink/eat<br>➢ Vomiting<br>➢ Convulsions<br>➢ Lethargy/Unconsciousness<br><br>**AND/OR**<br>**Hypoxemia (<92 SpO2 Blood Oxygen Level)**	**Severe pneumonia**<br><br>**Children of 0 – 6 days:**<br>1. Fast Breathing (≥ 60 breaths/min)<br><br>**Children of 7 days – 59 months:**<br>1. Fast Breathing and/or Chest In-drawing<br>➢ 0-2months: > 60 breaths/min<br>➢ 2-12months: ≥50 breaths/min<br>➢ 12-59months ≥40 breaths/min<br>AND<br>2. Any ONE General Danger Sign* and/or Stridor<br>➢ *Unable to drink/eat<br>➢ Vomiting<br>➢ Convulsions<br>➢ Lethargy/Unconsciousness

For peer review only

Appendix 2: Study Activities

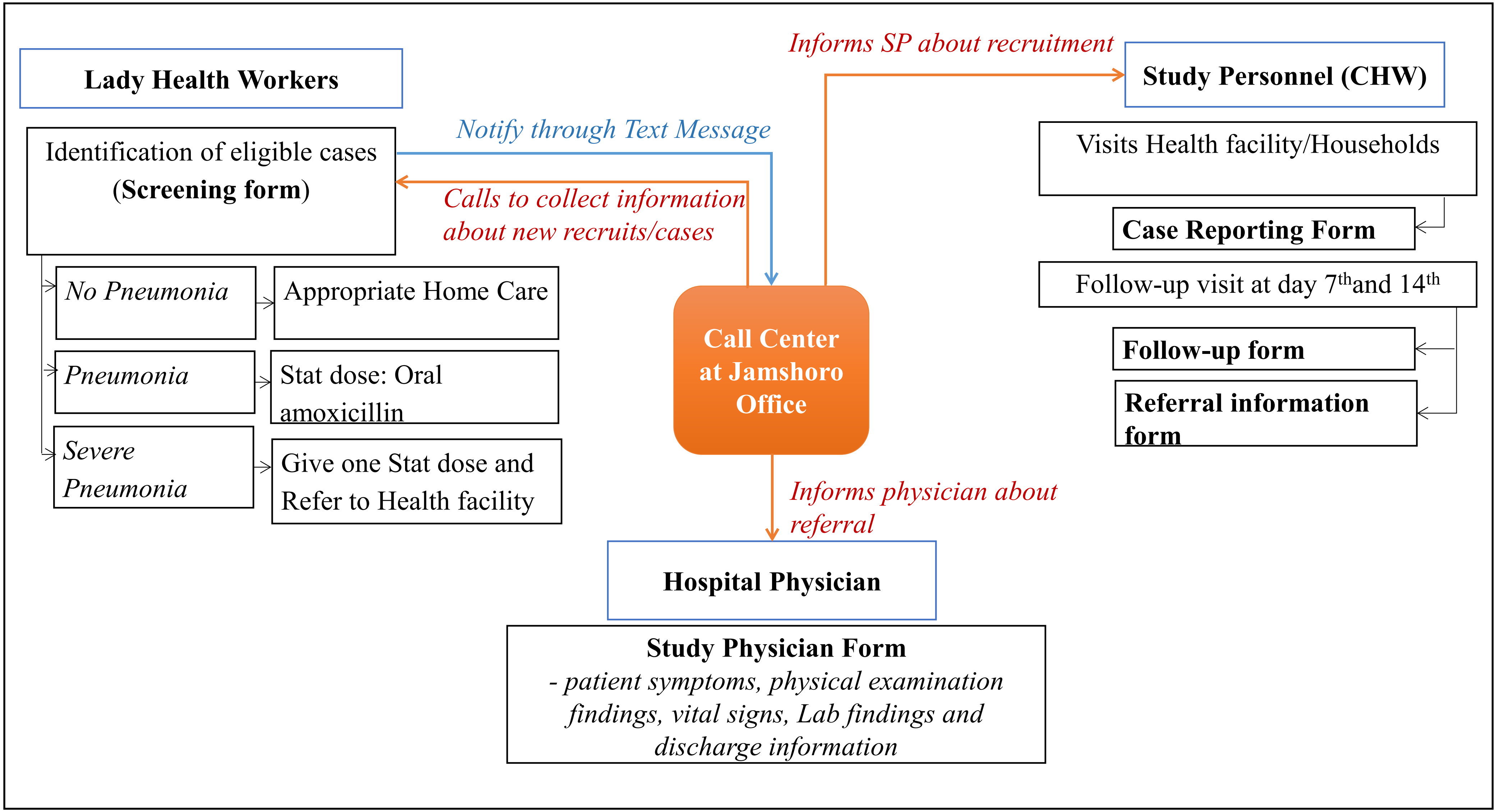

Appendix 3: Informed Consent

Research Consent Form

Title of Research Project:

Impact of Pulse Oximetry on Hospital Referral Acceptance in children under 5 with severe pneumonia in rural Pakistan (District Jamshoro): a cluster randomized trial (GAPPD Scale up Project)

Investigators:

Dr. Fatima Mir (Principal investigator PO Project, AKU)

Department of Pediatrics and Child Health, Aga Khan University, Karachi, Pakistan

Tel: 92-21-34864955

Dr. Sajid Soofi (PI, Scale up of GAPPD in Pakistan)

Centre of Excellence in Women & Child Health, The Aga Khan University, Pakistan.

Tel: 92-21-34864955

Professor Zulfiqar Bhutta (Senior Investigator, Scale up of GAPPD in Pakistan)

Centre of Excellence in Women & Child Health, The Aga Khan University, Pakistan.

Tel: 92-21-34864955

Purpose of the Research:

Severe Pneumonia is a serious and possibly life threatening infection in young children aged under 5 years. It may involve fast breathing, chest indrawing, and danger signs like inability to feed, convulsions, persistent vomiting and decreased movement. Hypoxemia is now recognized as an additional sign of severe pneumonia needing hospital admission. Pulse oximetry (the 'oxygen test') is a simple method whereby lady health workers can check level of oxygen in blood of children and decide which young babies need immediate medical care and referral to hospital for antibiotics and oxygen and which babies can be treated in the health center itself.

In this study, we are testing whether use of pulse oximetry in addition to other danger signs of severe pneumonia will influence families to accept hospital referral in comparison to areas where danger signs other than hypoxemia are used.

This project is a collaboration between Aga Khan University (AKU) in Karachi and the Gates Foundation, Seattle, USA.

For peer review only

Description of the Research:

We are inviting children aged 0 to 59 months screened at home to join this research study. If your baby joins the study, the following activities will take place:

1) **Questionnaire**. We will ask several questions about the health of your baby, as well as check him/her for presence of pneumonia. If he/she has signs of severe pneumonia (with or without hypoxemia), he will be eligible to take part in our study.

2) **Pulse oximetry**. Next your child may or may not undergo a pulse oximetry measurement dependent on whether he resides in an intervention or non-intervention cluster. The process will be painless for the child and will involve placing a sensor on your baby's foot or hand. Each sensor is attached to a pulse oximetry machine. A number on the screen of the machine shows the oxygen level. A level including and above 92% will be regarded as normal.

   All babies with fast breathing or chest in drawing pneumonia AND any one of general danger signs (inability to feed, persistent vomiting, decreased movement, convulsions) with or without hypoxemia (SaO_2 <92%) will be advised and helped to go to an assigned hospital for treatment (antibiotics and possibly oxygen supportive therapy). Their clinical status over there will be followed and transfer to hospital and subsequent treatment will be facilitated by the study.

   All responses will be documented on paper forms. We will arrange for quick referral to hospital if your baby has low oxygen level or other signs of serious illness. Follow-up visits by our study staff will be conducted to check the status of your baby.

3) You may be asked detailed questions based on whether you accept or refuse hospital referral to allow us to understand what factors contributed to your decision making. Since this process will take time, we will do it after the child's treatment plan has been started at hospital or at home.

4) **Video recording**. Some infants will be video recorded during the study. The researchers will look at these videos to make quality checks on study conduct and procedures. You can refuse video recording but still join the rest of the study. If you provide permission, some videos may be shown publicly for education purposes, but your child's name will be kept private. The videos or images from them will not be shown or sold for financial profit.

5) **Stored information**. This study is connected to other studies coordinated by the Aga Khan University. If you agree to join this study, we will access information about your baby collected as part of the Aga Khan University demographic surveillance system and other Aga Khan University studies to which you have already allowed, or will allow, your baby to join.

We expect to enroll about 4160 children with severe pneumonia in this study.

Potential Harms, Discomforts or Inconveniences:

There are no harms or discomforts that could be caused to your baby by taking part in this study.

Potential Benefits to individual participants:

Your baby will receive free treatment at the hospital if referral is accepted. The baby will also receive two follow up visits to check if he/she is recovering as expected on day 7 and 14 as part of the study. If you refuse to accept hospital referral for injectable antibiotics and oxygen, we will provide less ideal oral treatment options at home.

Potential Benefits to Society:

For peer review only

We may learn more about the usefulness of pulse oximetry for assessment of young babies in your community and other similar places.

Confidentiality:

We will respect your privacy. No information about you or your child will be given to anyone or be published without your permission, unless required by law. The paper forms, videos and electronic information made in this study will be stored in a secure, locked location. Only members of the research team will have access to them. BMGF (the sponsor) or AKU Clinical Research Monitors may look at your child's records to check on the study. By signing this consent form, you agree to let these people look at your child's records. We will put a copy of this research consent form in your child's patient health record and give you a copy as well. After the study has been completed, the forms and videos will be kept as long as required by BMGF and AKU policies. They will then be destroyed according to these same policies. Paper and electronic forms from this study will be stored for at least 7 years after publication of the study. Published study results will not reveal your identity or the identity of your baby.

Payment

No payment will be given for participation in this study.

Participation:

You decide if your child joins this study. If you choose to let your child take part, you can take your child out of the study at any time.

In the unlikely situation that your child becomes ill or is harmed because of study participation, we will treat your child for free. Your signing this consent form does not interfere with your legal rights in any way. The staff of the study, any people who gave money for the study, or the hospital are still responsible, legally and professionally, for what they do during this study.

Sponsorship:

The funder of this research is the Bill and Melinda Gates Foundation Seatttle, USA.

Conflicts of interest:

None of the researchers have any conflicts of interest to declare.

Consent:

By signing this form, you agree that:

1) The study has been explained to you.
2) All of your questions have been answered.
3) The possible harms and benefits of this study have been explained to you.
4) You may ask questions about the study now and in the future.
5) You have been told that your child's medical records will be kept private except as described to you.
6) You understand that information about your child will not be given to anyone or be published without first asking your permission.

I agree that my child____ may take part in this study.

I understand that videos may be used to assess the success of pulse oximetry. I understand that I may refuse to permit video-recording of my child, yet take part in other aspects of the study. I understand that even if I permit video-recording now, I may refuse the use of these videos at a later time. I understand that I may permit video-recording of my child for researchers to view, but refuse any public display of the videos.

For peer review only

I agree to permit my child and me to be video-recorded during this study.

☐ YES ☐ NO

I agree to permit whole or parts of videos of my child and me to be shown publicly for educational purposes.

☐ YES ☐ NO

______________________________ ______________________________

Printed Name of Parent/Legal Guardian | Parent/Legal Guardian's signature/Thumb print & date

______________________________ ______________________________

Printed Name of person who explained consent | Signature of Person who explained consent & date

______________________________ ______________________________

Printed Witness' name | Witness' signature & date

(If the parent/legal guardian does not read Sindhi)

If you have any questions about this study, please call Dr Fatima Mir at ____________________

If you have questions about your child's rights as a subject in a study or injuries during a study, please call Coordinator, Bioethics Unit at ____________________

For peer review only

STANDARD PROTOCOL ITEMS: RECOMMENDATIONS FOR INTERVENTIONAL TRIALS

SPIRIT 2013 Checklist: Recommended items to address in a clinical trial protocol and related documents*

Section/item	Item No	Description	Page numbers:
**Administrative information**			
Title	1	Descriptive title identifying the study design, population, interventions, and, if applicable, trial acronym	Title page
Trial registration	2a	Trial identifier and registry name. If not yet registered, name of intended registry	Page 1
	2b	All items from the World Health Organization Trial Registration Data Set	Page 1
Protocol version	3	Date and version identifier	
Funding	4	Sources and types of financial, material, and other support	Page 14
Roles and responsibilities	5a	Names, affiliations, and roles of protocol contributors	Title page and page 14
	5b	Name and contact information for the trial sponsor	
	5c	Role of study sponsor and funders, if any, in study design; collection, management, analysis, and interpretation of data; writing of the report; and the decision to submit the report for publication, including whether they will have ultimate authority over any of these activities	
	5d	Composition, roles, and responsibilities of the coordinating centre, steering committee, endpoint adjudication committee, data management team, and other individuals or groups overseeing the trial, if applicable (see Item 21a for data monitoring committee)	
**Introduction**			

For peer review only

Background and rationale	6a	Description of research question and justification for undertaking the trial, including summary of relevant studies (published and unpublished) examining benefits and harms for each intervention	Pages 3 - 5
	6b	Explanation for choice of comparators	Page 4
Objectives	7	Specific objectives or hypotheses	Page 5
Trial design	8	Description of trial design including type of trial (eg, parallel group, crossover, factorial, single group), allocation ratio, and framework (eg, superiority, equivalence, noninferiority, exploratory)	Page 5

Methods: Participants, interventions, and outcomes

Study setting	9	Description of study settings (eg, community clinic, academic hospital) and list of countries where data will be collected. Reference to where list of study sites can be obtained	Page 6
Eligibility criteria	10	Inclusion and exclusion criteria for participants. If applicable, eligibility criteria for study centres and individuals who will perform the interventions (eg, surgeons, psychotherapists)	Page 6
Interventions	11a	Interventions for each group with sufficient detail to allow replication, including how and when they will be administered	Pages 7 - 9
	11b	Criteria for discontinuing or modifying allocated interventions for a given trial participant (eg, drug dose change in response to harms, participant request, or improving/worsening disease)	Not applicable
	11c	Strategies to improve adherence to intervention protocols, and any procedures for monitoring adherence (eg, drug tablet return, laboratory tests)	Not applicable
	11d	Relevant concomitant care and interventions that are permitted or prohibited during the trial	

For peer review only - http://bmjopen.bmj.com/site/about/guidelines.xhtml

Outcomes	12	Primary, secondary, and other outcomes, including the specific measurement variable (eg, systolic blood pressure), analysis metric (eg, change from baseline, final value, time to event), method of aggregation (eg, median, proportion), and time point for each outcome. Explanation of the clinical relevance of chosen efficacy and harm outcomes is strongly recommended	Pages 5-6
Participant timeline	13	Time schedule of enrolment, interventions (including any run-ins and washouts), assessments, and visits for participants. A schematic diagram is highly recommended (see Figure)	Page 6
Sample size	14	Estimated number of participants needed to achieve study objectives and how it was determined, including clinical and statistical assumptions supporting any sample size calculations	Page 12
Recruitment	15	Strategies for achieving adequate participant enrolment to reach target sample size	Pages 7 - 9

Methods: Assignment of interventions (for controlled trials)

Allocation:

Sequence generation	16a	Method of generating the allocation sequence (eg, computer-generated random numbers), and list of any factors for stratification. To reduce predictability of a random sequence, details of any planned restriction (eg, blocking) should be provided in a separate document that is unavailable to those who enrol participants or assign interventions	Pages 6 - 7
Allocation concealment mechanism	16b	Mechanism of implementing the allocation sequence (eg, central telephone; sequentially numbered, opaque, sealed envelopes), describing any steps to conceal the sequence until interventions are assigned	Pages 6 - 7
Implementation	16c	Who will generate the allocation sequence, who will enrol participants, and who will assign participants to interventions	Pages 6 - 8
Blinding (masking)	17a	Who will be blinded after assignment to interventions (eg, trial participants, care providers, outcome assessors, data analysts), and how	

	17b	If blinded, circumstances under which unblinding is permissible, and procedure for revealing a participant's allocated intervention during the trial	
**Methods: Data collection, management, and analysis**			
Data collection methods	18a	Plans for assessment and collection of outcome, baseline, and other trial data, including any related processes to promote data quality (eg, duplicate measurements, training of assessors) and a description of study instruments (eg, questionnaires, laboratory tests) along with their reliability and validity, if known. Reference to where data collection forms can be found, if not in the protocol	Page 9
	18b	Plans to promote participant retention and complete follow-up, including list of any outcome data to be collected for participants who discontinue or deviate from intervention protocols	Page 12
Data management	19	Plans for data entry, coding, security, and storage, including any related processes to promote data quality (eg, double data entry; range checks for data values). Reference to where details of data management procedures can be found, if not in the protocol	Page 10
Statistical methods	20a	Statistical methods for analysing primary and secondary outcomes. Reference to where other details of the statistical analysis plan can be found, if not in the protocol	Page 12
	20b	Methods for any additional analyses (eg, subgroup and adjusted analyses)	Page 12
	20c	Definition of analysis population relating to protocol non-adherence (eg, as randomised analysis), and any statistical methods to handle missing data (eg, multiple imputation)	
**Methods: Monitoring**			
Data monitoring	21a	Composition of data monitoring committee (DMC); summary of its role and reporting structure; statement of whether it is independent from the sponsor and competing interests; and reference to where further details about its charter can be found, if not in the protocol. Alternatively, an explanation of why a DMC is not needed	Page 10

	21b	Description of any interim analyses and stopping guidelines, including who will have access to these interim results and make the final decision to terminate the trial	
Harms	22	Plans for collecting, assessing, reporting, and managing solicited and spontaneously reported adverse events and other unintended effects of trial interventions or trial conduct	Page 11
Auditing	23	Frequency and procedures for auditing trial conduct, if any, and whether the process will be independent from investigators and the sponsor	
**Ethics and dissemination**			
Research ethics approval	24	Plans for seeking research ethics committee/institutional review board (REC/IRB) approval	Page 14
Protocol amendments	25	Plans for communicating important protocol modifications (eg, changes to eligibility criteria, outcomes, analyses) to relevant parties (eg, investigators, REC/IRBs, trial participants, trial registries, journals, regulators)	Page 10-11
Consent or assent	26a	Who will obtain informed consent or assent from potential trial participants or authorised surrogates, and how (see Item 32)	Page 6
	26b	Additional consent provisions for collection and use of participant data and biological specimens in ancillary studies, if applicable	
Confidentiality	27	How personal information about potential and enrolled participants will be collected, shared, and maintained in order to protect confidentiality before, during, and after the trial	Page 14
Declaration of interests	28	Financial and other competing interests for principal investigators for the overall trial and each study site	Page 14
Access to data	29	Statement of who will have access to the final trial dataset, and disclosure of contractual agreements that limit such access for investigators	Page 14
Ancillary and post-trial care	30	Provisions, if any, for ancillary and post-trial care, and for compensation to those who suffer harm from trial participation	

For peer review only

Dissemination policy	31a	Plans for investigators and sponsor to communicate trial results to participants, healthcare professionals, the public, and other relevant groups (eg, via publication, reporting in results databases, or other data sharing arrangements), including any publication restrictions	Page 14
	31b	Authorship eligibility guidelines and any intended use of professional writers	Page 14
	31c	Plans, if any, for granting public access to the full protocol, participant-level dataset, and statistical code	
**Appendices**			
Informed consent materials	32	Model consent form and other related documentation given to participants and authorised surrogates	
Biological specimens	33	Plans for collection, laboratory evaluation, and storage of biological specimens for genetic or molecular analysis in the current trial and for future use in ancillary studies, if applicable	

*It is strongly recommended that this checklist be read in conjunction with the SPIRIT 2013 Explanation & Elaboration for important clarification on the items. Amendments to the protocol should be tracked and dated. The SPIRIT checklist is copyrighted by the SPIRIT Group under the Creative Commons "Attribution-NonCommercial-NoDerivs 3.0 Unported" license.

For peer review only

BMJ Open

Impact of Pulse Oximetry on Hospital Referral Acceptance in Children under 5 with Severe Pneumonia in Rural Pakistan (District Jamshoro): Protocol for a Cluster Randomized Trial

Journal:	*BMJ Open*
Manuscript ID	bmjopen-2020-046158.R2
Article Type:	Protocol
Date Submitted by the Author:	03-Aug-2021
Complete List of Authors:	Mir, Fatima; Aga Khan University, Pediatrics and Child Health; Aga Khan University, Aga Khan University<br>Ali Nathwani, Apsara; Aga Khan University,<br>chanar, suhail; Aga Khan University, Paediatrics and child health<br>Hussain, Amjad; Aga Khan University, Pediatrics and Child Health<br>Rizvi, Arjumand; Aga Khan Medical University, Pediatrics and Child Health<br>Ahmed, Imran; Aga Khan University<br>Memon, Zahid Ali; Aga Khan University, Centre of Excellence in Women and Child Health<br>Habib, Atif; Aga Khan University, Department of Paediatrics & Child Health<br>Soofi, Sajid; Aga Khan University Hospital, Paediatric & Child Health<br>Bhutta, Zulfiqar; Aga Khan University, Division of Women and Child Health
**Primary Subject Heading**:	Global health
Secondary Subject Heading:	Health services research, Infectious diseases, Paediatrics, Public health
Keywords:	Public health < INFECTIOUS DISEASES, Paediatric infectious disease & immunisation < PAEDIATRICS, PUBLIC HEALTH, RESPIRATORY MEDICINE (see Thoracic Medicine), Clinical trials < THERAPEUTICS

SCHOLARONE™
Manuscripts

I, the Submitting Author has the right to grant and does grant on behalf of all authors of the Work (as defined in the below author licence), an exclusive licence and/or a non-exclusive licence for contributions from authors who are: i) UK Crown employees; ii) where BMJ has agreed a CC-BY licence shall apply, and/or iii) in accordance with the terms applicable for US Federal Government officers or employees acting as part of their official duties; on a worldwide, perpetual, irrevocable, royalty-free basis to BMJ Publishing Group Ltd ("BMJ") its licensees and where the relevant Journal is co-owned by BMJ to the co-owners of the Journal, to publish the Work in this journal and any other BMJ products and to exploit all rights, as set out in our licence.

The Submitting Author accepts and understands that any supply made under these terms is made by BMJ to the Submitting Author unless you are acting as an employee on behalf of your employer or a postgraduate student of an affiliated institution which is paying any applicable article publishing charge ("APC") for Open Access articles. Where the Submitting Author wishes to make the Work available on an Open Access basis (and intends to pay the relevant APC), the terms of reuse of such Open Access shall be governed by a Creative Commons licence – details of these licences and which Creative Commons licence will apply to this Work are set out in our licence referred to above.

Other than as permitted in any relevant BMJ Author's Self Archiving Policies, I confirm this Work has not been accepted for publication elsewhere, is not being considered for publication elsewhere and does not duplicate material already published. I confirm all authors consent to publication of this Work and authorise the granting of this licence.

For peer review only

Impact of Pulse Oximetry on Hospital Referral Acceptance in Children under 5 with Severe Pneumonia in Rural Pakistan (District Jamshoro): Protocol for a Cluster Randomized Trial

Fatima Mir[1]*, Apsara Ali Nathwani[2], Suhail Chanar[2], Amjad Hussain[2], Arjumand Rizvi[2], Imran Ahmed[2], Zahid Memon[2], Muhammad Atif Habib[2], Sajid Soofi[1,2], Zulfiqar A Bhutta[2]

Affiliations

[1]Department of Pediatrics and Child Health, The Aga Khan University, Pakistan

[2]Centre of Excellence in Women & Child Health, The Aga Khan University, Pakistan.

***Corresponding author**

Dr. Fatima Mir

Associate Professor

Department of Pediatrics and Child Health, the Aga Khan University

Stadium Road, Karachi 74800

Pakistan

Email: fatima.mir@aku.edu

Phone: +92 3005844610

Key words: Severe pneumonia, hypoxemia, pulse-oximetry, Lady Health Workers (LHWs), community case management pneumonia, hospital referral.

For peer review only

Abstract

Background: Pneumonia is a leading cause of death among children under five specifically in South Asia and Sub-Saharan Africa. Hypoxemia is a life-threatening complication among children under five with pneumonia. Hypoxemia increases risk of mortality by 4.3 times in children with pneumonia than those without hypoxemia. Prevalence of hypoxemia varies with geography, altitude and severity (9-39% Asia, 3-10% African countries). In this protocol paper we describe research methods for assessing impact of lady health workers identifying hypoxemia in children with signs of pneumonia during household visits on acceptance of hospital referral in District Jamshoro, Sindh.

Methods and Analysis: A cluster randomized controlled trial using pulse oximetry as intervention for children with severe pneumonia will be conducted in community settings. Children aged 0-59 months with signs of severe pneumonia will be recruited by Lady Health Workers (LHWs) during routine visits in both intervention and control arms after consent. Severe pneumonia will be defined as fast breathing and/or chest in-drawing, and, one or more danger sign and/or hypoxemia (Sa02<92%) in PO (intervention) group and fast breathing and/or chest in-drawing and 1/more danger sign in clinical signs (control) group. Recruits in both groups will receive a stat dose of oral amoxicillin and referral to designated tertiary health facility. Analysis of variance will be used to compare baseline referral acceptance in both groups with that at end of study.

Ethics and Dissemination: Ethical approval was granted by the Ethics Review Committee of the Aga Khan University (4722-Ped-ERC-17), Karachi. Study results will be shared with relevant government and non-governmental organizations, presented at national and international research conferences and published in international peer-reviewed scientific journals.

Trial registration: Clinicaltrials.gov Registry NCT03588377. Trial registration Date: May 05, 2018. Last update Date: July 26, 2021. Study Status: ongoing

Article Summary

Strengths and Limitations of this study

- Our study will directly assess impact of pulse oximetry on family referral acceptance by comparing family acceptance of hospital referral (verbal acceptance with hospital visit) in ‘pulse oximetry and clinical signs’ group with ‘clinical signs alone’ group

For peer review only - http://bmjopen.bmj.com/site/about/guidelines.xhtml

- The study has applicability because it assesses impact with ‘real-life’ limitations (variability in lady health worker accuracy in identifying severe pneumonia, availability of oxygen and human resource at referral hospital and, availability of private transportation)
- The study design provides means of minimizing the effect of confounding
- The study design avoids bias in allocation to exposure groups
- Blinding is not possible due to nature of intervention
- Some subjects may fail to adhere to protocol and non-adherence may cause an underestimated measure of association.

For peer review only

Introduction

Pneumonia accounts for an estimated 18% of under-5 mortality across the globe [1] Majority of these pneumonia-specific deaths occur in 15 countries, in which Pakistan ranks fifth. [2] Failure to seek early care and delays in hospital referral are commonly acknowledged determinants of mortality in childhood pneumonia with a higher proportion reported from rural settings than urban. [3-6] Acceptance rates of 'facilitated' hospital referral advice have been reported low between 8% and 23% for sick young infants in peri-urban Karachi. [7, 8] They are even lower for non-facilitated referral in rural settings in children under 5 with severe pneumonia in rural Matiari district, Sindh. [5] The prominent reasons in developing countries behind this delay are inability to recognize seriousness of pneumonia, distance from health facility and lack of money for private health care. [9, 10]

In 1994, the Government of Pakistan introduced the Lady Health Worker (LHW) Program in rural populations with low physician density to address common health problems in women and children under 5 through household visits. To date, a team of over 110,000 LHWs are working for the program nationwide with 23,185 LHWs in Sindh alone [11]. Each LHW is responsible for a population of 1000–1500 individuals (catchment of about 100 families). Recruits are preferably local, with a minimum of 8 years of formal schooling followed by 15 months of training to deliver maternal and child health (MCH) care in community settings. During a monthly home visit, the LHW provide essential maternal and child health care services including family planning needs, nutritional assessments of both mother and child, management of minor and common illnesses, improving immunization coverage and imparting health education. [12]

This study recognizes the LHW as a powerful conduit for reaching under 5 children at household level and identifying severe pneumonia in under five at an early stage. It also recognizes the underestimation of hypoxemia (SpO_2 of <90%), a major risk factor for pneumonia mortality at community level and assesses its prevalence. The reported prevalence of hypoxemia in under 5 acutely ill children is 5 to 58% in facilities [13-16] and 16 to 39% in community settings. [17] A 4.3 times higher risk of mortality has been associated with pneumonia with hypoxemia than in children with pneumonia without hypoxemia. [13] Hypoxemia is also predictive of treatment failure with amoxicillin in 3-59 months old children. [18] In resource poor settings where pulse oximetry is not feasible, signs and symptoms of severe pneumonia (sleepiness, cyanosis, head

For peer review only - http://bmjopen.bmj.com/site/about/guidelines.xhtml

nodding/grunting, and inability to move) are used as predictors of hypoxemia. However, the validity of clinical signs to predict hypoxemia varies and it is often difficult for physicians working in settings, where objective detection of hypoxemia is not available, to decide whether the child coming with severe pneumonia requires administration of oxygen or not. [15, 19, 20]

Pulse oximetry [16] is a rapid, portable, non-invasive and accurate method of measuring arterial hemoglobin oxygenation (Sp02) and has therefore been used in trial and clinical settings to detect hypoxemia. Appropriate oxygen therapy (based on PO findings rather than clinical signs of severity alone) has been associated with lower mortality risk. [19] Assuming access to supplemental oxygen, PO could potentially avert up to 148,000 severe pneumonia related deaths if implemented, and, combining PO with IMCI assessment for pneumonia has been shown to be cost effective in 15 high burden countries. [21] Emdin et al found first level Lady Health Workers in peri-urban Karachi could easily perform pulse oximetry on young infants on well and sick visits to a primary health care facility. [22]

Over the past decade, the possible impact of pulse oximetry in hospital and community settings has been of interest across the globe. Health survey of 54 countries in 2010, suggested that 19.2% of the operating theatres around the globe are not equipped with pulse oximeters [23]. Trials assessing utility of pulse oximetry on a health systems level in Nigeria have shown that health workers reserve PO for the sickest patients [24, 25]. This has also been observed in areas at higher altitudes with higher prevalence of hypoxemia (highlands of Papua New Guinea) [25, 26]. There is lack of clarity about how pulse oximetry can be used in the community where lower hypoxemia prevalence may be a lesser incentive for health workers to use PO such as in interior Sindh settings in Pakistan. Then again, health workers recruited from within communities may be better invested and motivated than hospital personnel in following case management guidelines precluding PO. It is therefore worthwhile to explore and describe contexts behind a family's acceptance (or not) of referral advice whether based on technology and/or clinical examination in rural settings where the highest burden of pneumonia deaths lies.

Feasibility and sustainability audits of oxygen delivery systems in the Gambia and Egypt have shown that providing technology alone is ineffective, and should preclude provision of supplies, education, training and feedback [27-30]. This protocol paper describes a study to assess the effect

For peer review only - http://bmjopen.bmj.com/site/about/guidelines.xhtml

of PO monitoring in community settings on hospital referral acceptance in children under 5 with severe pneumonia.

Methods

Study aims and design:

The overall aim of the study is to assess if detection of hypoxemia, and/or severe pneumonia in children 0-59 months by LHWs during their monthly home visits will increase hospital referral acceptance among families in District Jamshoro, Sindh, Pakistan.

Primary objectives are:

1) To assess and compare the impact of 'pulse oximetry' used by LHWs at household level on increasing hospital referral acceptance rates in intervention clusters (district Jamshoro) for 0-59 months old children with severe pneumonia with the impact of LHWs using clinical signs alone in non-intervention clusters of the same district

2) To investigate the likely predictors (demographic, clinical) of hospital referral acceptance in both the groups

Secondary objective is:

3) To compare clinical outcomes (treatment completion, treatment failure, hypoxemia) of children 0-59 months who accepted referral to those who refused admission and were treated at home

A community-based cluster randomized trial will be conducted in district Jamshoro, among children of ages 0-59 months **(Figure 1)**. In intervention areas, all children with cough, fever or difficulty in breathing (acute respiratory illness) will be assessed for study eligibility (signs and symptoms of severe pneumonia, OR hypoxemia alone, OR severe pneumonia with hypoxemia) by LHWs during their monthly home visit in their catchment area. In control areas, study eligibility will require detection of signs of severe pneumonia alone (**Appendix 1**) during LHW monthly visits. Data will be collected on demographics, likely predictors, and clinical outcomes using a structured questionnaire.

The primary outcome is hospital referral acceptance in children under 5 with severe pneumonia and to identify demographic and clinical predictors of hospital referral acceptance. The predictors

For peer review only - http://bmjopen.bmj.com/site/about/guidelines.xhtml

will include distance of child home to referral facility, socio-economic status of household, parental education, child age, nutritional status, respiratory rate, temperature, hypoxemia and presence of other illnesses.

Secondary outcomes include duration of oxygen therapy, treatment failure, duration of hospital stay, vital and health status of child at day 7th and 14th.

Trial setting:

The study will be conducted within the community of Taluka Kotri in District Jamshoro, Sindh **(Figure 2)**. Jamshoro District has a population of 993,142 [31]. It is predominantly rural, with 33% literacy, and >50% employed daily wage laborers. A baseline survey conducted as a part of current study showed the status of overall health indicators: skilled birth attendance 57%, antenatal care coverage 75%, postnatal care cover for mother and newborn within 48 hours 31.5%, vaccination completeness in children 12-23 months 68% and care seeking for ARI and diarrhea >80% (internal survey). A total of 27 health facilities function in the district including one District Headquarter (DHQ) Hospital, three Taluka Headquarter (THQ) Hospitals, five Rural Health Centers (RHCs) and 18 Basic Health Units (BHUs). The district is divided administratively in 30 union councils. Kotri is one of the 4 Talukas of Jamshoro, consists of 44% (437,561) of the population of district [31].

Participants will be the permanent residents of Kotri, Jamshoro and recruited from their homes during routine monthly visits by Lady Health Workers serving in their catchment areas. This study is expected to run for 48 months with participant identification and enrolment conducted simultaneously in intervention and control clusters over 21 months after an initial pilot of one month. Each enrolled child will be revisited at day 7 and 14 for outcome measurement.

Participant:

Any child aged 0-59 months having signs and symptoms of acute respiratory illness (cough, fever, difficulty in breathing) in intervention clusters will undergo assessment of 1) signs and symptoms of severe pneumonia and, 2) pulse oximetry during monthly LHW home visits). Presence of severe pneumonia with or without hypoxemia, or hypoxemia alone will merit hospital referral (non-facilitated). Any child aged 0-59 months having signs and symptoms of acute respiratory illness (cough, fever, difficulty in breathing) in control clusters will undergo assessment of 1) signs and

For peer review only - http://bmjopen.bmj.com/site/about/guidelines.xhtml

symptoms of severe pneumonia alone. Presence of severe pneumonia will merit hospital referral (non-facilitated). Exclusion criteria will include lack of consent and, non-availability (lost to follow up, migration) on days of scheduled follow-up visits (days 7 and 14). Those who accepted referral but did not show up at hospital will be included in final analysis.

Randomization and masking:

Study clusters were defined as the area covered by an LHW. Each LHW covers a minimum of 100 households. A list of LHWs working in Kotri was collected from the LHW program, Health Department Government of Sindh. There is a total of 188 active LHWs in the study site. A baseline survey was conducted to collect data on health indicators from the LHW catchments. The clusters were randomly allocated to intervention and control groups on 1:1 fashion with a computer-generated randomization sequence that was generated by an independent expert. Clusters were matched on under-5 population and distance to referral health facility. No stratification was used for allocation; clusters were selected to ensure that the reporting and training centres of intervention and control LHWs were separate. The investigators and the national and provincial LHW program coordinators will be excluded from the allocation process.

Training of Lady Health Workers:

The Lady Health Worker (LHW) program of Pakistan consists of a community-based group of first-level health workers with the principal mandate of home-based Maternal and Child Health. The recruitment process is well defined and selection criteria include: at least 8 years of education with middle school pass, local residency, recommendation from the community, and preferably married [32]. Once selected, they receive 15 months of basic training in mid-wifery and family planning using standardized training manuals and curriculum, and periodic refresher training courses. Each of these LHWs is typically responsible for approximately 1000 people, or 150 homes, and often serve as the primary health care contact in these rural communities [32, 33].

We chose LHWs as study personnel due to their access to homes on regular monthly basis. We trained intervention and control LHWs in separate groups for all of the following: 1) classification of ARI (no pneumonia, pneumonia, severe pneumonia) using standard acute respiratory infections

For peer review only - http://bmjopen.bmj.com/site/about/guidelines.xhtml

training modules (WHO and IMNCI) 2) identification of danger signs 3) case management of pneumonia at home with oral amoxicillin and severe pneumonia with stat dose of antibiotic before hospital referral. Intervention LHWs received an additional training in using a pulse oximeter and obtaining a valid reading. The Principal Investigator led these training sessions with senior trainers of the LHW program (Lady Health Supervisors).

Participant Recruitment and Study Procedures:

Intervention delivery

Children aged 0–59 months with cough and/or difficult breathing during regular home visits of LHWs will be assessed for firstly, signs and symptoms of severe pneumonia (fast breathing/chest in-drawing and one or more danger sign (unable to eat/drink, vomiting, convulsion and lethargy/unconsciousness) and/or Stridor)) and secondly, hypoxemia (SpO2 <92%) using a handheld pulse oximeter (Masimo Rad-5v) to measure blood oxygen saturation level. LHWs will also do case management of children with pneumonia and severe pneumonia. A 3-day course of oral amoxicillin will be given to children with pneumonia at home, whereas children meeting referral criteria (severe pneumonia alone, hypoxemia alone, or severe pneumonia and hypoxemia) after obtaining informed consent (**Appendix 2**), will be administered a stat dose of oral amoxicillin and referred to nearest referral hospital (DHQ Kotri).

The study investigators will have provided these pulse oximeters to the LHW Program in advance and highlighted which ones will receive them. Physicians at the referral center serving the intervention clusters will also receive handheld pulse oximeters. All the LHWs and staff will be trained on the use, and maintenance of these pulse oximeters. Children with severe pneumonia with or without hypoxemia will be advised to go to hospital for antibiotics and oxygen, using the PO reading as a tool to convince parents. Children with hypoxemia alone, without signs of severe pneumonia will be referred to hospital to rule out cyanotic congenital heart disease. Name of the pre-designated health facility with available oxygen and study physician will be provided to all the LHWs so that Study Workers (non-LHW study personnel) can coordinate with Study Physicians and ensure the patient has reached and is receiving safe and recommended care at referral facility. Project staff will pretest and regularly monitor PO accuracy and quality of readings.

Hypoxemia will be defined as an arterial oxygen saturation (SpO2) <92%. SpO2 measurement will be recorded after 1 minute of stable observation. If the SpO2 comes 92% or less, the child will

For peer review only - http://bmjopen.bmj.com/site/about/guidelines.xhtml

first be assessed for nasal obstruction with readings repeated after applying nasal saline drops. If repeat reading shows hypoxemia, the child will be referred to nearest designated referral hospital and admitted for oxygen via nasal or nasopharyngeal route and intravenous antibiotics, as per recommendations.

Implementation of active control: Clinical Signs assessment

Children aged 0–59 months with cough and/or difficult breathing during regular home visit will be assessed by LHWs for signs and symptoms of severe pneumonia (fast breathing/chest in-drawing and one or more danger sign (unable to eat/drink, vomiting, convulsion and lethargy/unconsciousness) and/or Stridor)). A 3-day course of oral amoxicillin will be given to children with pneumonia at home, whereas children with severe pneumonia (eligible for recruitment) will be requested for informed consent and offered stat dose of oral amoxicillin and referral to nearest referral hospital.

Procedure at referral facility

Children who accept hospital referral in both intervention and control clusters and reach hospital premises with LHW referral slip will be assessed by study physician at the referral center. An SMS notification with brief details of referred child will have been provided to trained study personnel (study physician) in advance at time of referral at both the referral facilities. Children with severe pneumonia and/or hypoxemia as per LHWs who reach referral hospital premises will be examined and subjected to pulse oximetry again by the study physician at referral facility. If signs and symptoms of severe pneumonia are present, the child will be admitted for further appropriate treatment (Oxygen therapy via nasal or nasopharyngeal route and intravenous antibiotics etc.) and if the symptoms are not severe (absence of danger sign), the child will be treated in outpatient care as per the standard of referral facility. All the children admitted at referral facility will undergo 12 hourly monitoring by study personnel and filling of case reporting form (CRF) and hospital physician form (HPF) at day 1, 7 and 14. Those children who refused the referral will be visited by study community health workers after 24 hours to confirm referral refusal and to fill CRF.

Preliminary meetings will be held with the Executive Director Health Jamshoro, Director General Health Sindh, In-charge Lady Health Worker Program Sindh and In-charge Pediatric Units LUMHS to ensure their cooperation through study duration. Emergency and pediatric unit staff at the referral facilities along with study personnel (physician/nurse) will be trained on

For peer review only - http://bmjopen.bmj.com/site/about/guidelines.xhtml

management of severe pneumonia according to the integrated management of neonatal and childhood illnesses (IMNCI) guidelines [34]. A baseline survey will be conducted at the health facilities to ensure availability of oxygen and necessary intravenous antibiotics. Even though it is ideal to guarantee sustainable oxygen systems at the two chosen referral public sector hospitals, this study does not provide oxygen and therefore aims to assess 'real-life' situations in public hospitals and their impact on severe pneumonia outcomes with or without hypoxemia. LHWs will be incentivized on basis of their contribution to the study activities.

Data Collection and Storage

Data will be collected by LHWs during house visits (Screening form), community health workers (during follow up visits day 1, 7 and 14) and hospital based study personnel (for all who accept referral and reach hospital premises) on paper forms **(Appendix 3)**. Given that it will be a new experience for LHWs to assess, classify and manage ARI cases and at the same time record findings on data forms accurately, these will be supervised closely and frequently, at least for the first pneumonia season. Well trained study field supervisory officers and LHW supervisors will be required to perform regular field supervision in their respective clusters and ensure accurate and logically entered data forms and make necessary verifications and corrections at the data collection sites and give feedback to the LHW to avoid repeating the errors. Raw data brought to the program office will be checked once again for accuracy by the technical staff and approved for entry in the computer. All raw data will be safely kept in the AKU office, appropriately numbered by cluster, until seven years after the study is over.

Case History Records

These include the study case report forms (CRF) and hospital physician form (HPF) that will contain information that documents the child's eligibility to participate in the study, the signed consent form, and information from tests and examinations. Wherever possible copies of supporting documentation for the information contained in the CRF should be kept with each patient's case history record. This supporting documentation may include records of physical examinations, progress notes, laboratory reports, X-rays, consultations, correspondence, information and data on the subject's condition, during and after the clinical investigation, diagnoses made, concomitant therapy, etc. All information in the case history records should be attributable to a specific individual. Since the CRF will not contain the patient's name, there will

For peer review only - http://bmjopen.bmj.com/site/about/guidelines.xhtml

be a unique link between the ID number on the CRF and the patient's name. Each child's case history record will be evaluated to verify validity and completeness of the data on the CRF when a study monitor visits the study site. All corrections to CRF's must be made without obscuring the original entry. The revised entry should be inserted and the person making the correction should sign and date the correction. Only authorized study personnel may complete or correct case report forms.

Data Management

Screening data will be collected on paper by LHWs. Case reporting form (CRF) and Hospital physician form will be collected on electronic forms. To ensure proper implementation of the intervention, the field supervisors will make spot checks and will arrange monthly refresher group sessions of the first-line health workers in which the problems encountered will be discussed and resolved. In addition, the data collection activity will be carried out by teams consisting of LHWs/CHWs and study staff will be further monitored by field supervisors who will perform a check on a subset (5%) of households.

An information system will be set up to keep track all patients screened and enrolled and a filing system to keep all study related records - case history records, study protocol or related documentation and drug distribution records. The coordinator at the site will be responsible for the completeness and accuracy of all the study materials.

Study Protocol and Related Documentation

All study related documents including the study protocol, manuals of operations, all correspondence sent to or received from the study monitor, materials used for obtaining informed consent, protocol modifications and records of the Institutional Review Board approval and all communications with the IRB must be maintained in complete form. These documents will be evaluated to ensure that study documentation is complete and current when a study monitor visits the study site.

Record Retention

Retention of accurate and complete records is essential to establish the validity and completeness of the study. All records must be retained for 7 years after the data set is frozen. Electronic data

For peer review only

For peer review only - http://bmjopen.bmj.com/site/about/guidelines.xhtml

will be de-identified, unlinked from any personal identifiers and therefore will protect individual identity.

Reporting of Serious Adverse Events and Treatment Failures

Amoxicillin is in widespread use and is not investigational in any study site. However, since oral amoxicillin is not routinely recommended for initial treatment of children who have severe pneumonia, the appropriate case report forms describing the occurrence of a serious adverse event, treatment failure or death must be faxed to the coordinating center within 72 hours of the site coordinator knowing about the event. Adverse events, treatment failure and death must be reported to allow appropriate interpretation of this critical information. If the outcome of the adverse event is unknown when the site coordinator first notifies the coordinating center, a follow-up form must be faxed to the coordinating center within 10 days of knowing about the event. The PI should send a copy of the adverse event data to their local IRB as soon as possible. The coordinating center will summarize the Adverse Event and Death information and send a report to the IRB/ERC of sponsors and to site IRBs. Both the rate of adverse events and the rate of patient accrual at each individual site will be monitored to determine if stopping rules are met. We do not anticipate serious adverse events. However, in case of one, a DSMB will be requested for and convened on ad hoc basis for safety review at any time during the study if there is a concern regarding rates of adverse events or rates of patient accrual. Adverse events will be reported by the study physician to the principal investigator and clinically managed by the study physician in conjunction with other physicians at the institution. Any related and unexpected life-threatening adverse event including death will be reported to the IRB within 2 business days as per IRB protocol and any related, unexpected and serious adverse event will be reported to the IRB within 10 business days as per IRB protocol.

Compliance with and Deviations from the Study Protocol

The Site coordinator must agree with and sign the protocol and confirm in writing that he or she has read, understands and will work according to the protocol and Good Clinical Practice. The Site coordinator is responsible for making sure that the protocol is strictly followed and should not make any changes to the study unless necessary to eliminate an apparent immediate hazard or damage to a trial subject. Any deviations from the study protocol including but not limited to inappropriate enrollment of a study subject, administration of the wrong study treatment, missed

For peer review only - http://bmjopen.bmj.com/site/about/guidelines.xhtml

doses of study treatment, missed observation points, incorrect administration of concomitant medications, etc. should be reported to the coordinating center and each site's IRB. The report should include a plan to rectify any problems at the site that may have caused the protocol deviation.

Sample size:

Defining a cluster as (the catchment area of) one Lady Health Worker (LHW), and assuming a power of 90% in detecting 50% increase in referral acceptance from a 10 % baseline to 15% among 0-59 month old children with severe pneumonia (pneumonia prevalence at 2 week recall (MICS Sindh):7.5% (18% of which is assumed severe Pneumonia) with ICC 0.001736. We need to capture a total of 4160 children with severe pneumonia in both intervention and control groups.

Data Analysis:

The primary analysis for each outcome will perform on an 'intention-to-treat' (ITT) basis, i.e. all children included in the analysis who were enrolled in the study according to the group to which they were allocated. All analyses will account for the cluster-randomized design to ensure correct type I error rates and confidence intervals [35]. Baseline characteristics will be compared by analyzing differences in means and proportions among the study arms. Categorical outcomes will be compared using chi square test and continuous outcomes using Student's t-test. For analysis of predictors of referral acceptance, generalized linear model will be used with logit link function. The univariate analysis will be conducted to explore the independent effect of each predictor on outcome. The variables significant at a liberal p-value of <0.20 will be included in multivariate model for adjustment. The results will be reported as relative risk (RR) with 95% CI. Type 1 error will be set at 5% level. All analysis will be done using STATA version 15.

Patient and Public Involvement

Patients or the public will not be involved in the design or conduct of the study. Results will be disseminated to the community.

Study Status

Recruitment began in August 2019 and field activities and data collection are in process. As of 16th October 2020, a total of 235 cases and 184 controls have been enrolled. Extension of study duration is in discussion to achieve sample size.

For peer review only - http://bmjopen.bmj.com/site/about/guidelines.xhtml

Discussion

Hypoxemia, a frequent complication of severe pneumonia, is a major risk factor for death in children under 5. Theoretically, detection of hypoxemia at community level among severe pneumonia cases by the use of pulse-oximeter would give awareness to the caregivers about severity of illness and reduce delay in hospital referral. Delayed care-seeking is a recognized risk factor in pneumonia mortality in community settings [36]. Unfortunately, information on prevalence of hypoxemia, effectiveness of its detection in influencing parents to seek hospital care (gold standard for severe pneumonia), and impact of appropriate care at hospital in settings like Pakistan is lacking. Thus, the findings of this study will build evidence for utility of providing front-line workers like LHWs with a tool to detect hypoxemia if signs and symptoms of pneumonia are present. Interestingly various groups across the world are now espousing pulse-oximetry with other strengths like detection of congenital heart disease [37, 38] and newborn sepsis [39].

Limitations

Some union councils with poor LHW coverage were not included in this study. This was a compromise on generalizability in favour of feasibility. It may have led to exclusion of children whose referral patterns were important to gauge. We also did not offer facilitated referral in either arm. This may have affected referral acceptance in both groups however will allow assessment of real-life impact of transportation barriers.

Though we kept hospital referral (as recommended by WHO) for severe pneumonia as our standard of care, it was beyond the scope of this study to ensure that referral centres had sustainable oxygen systems/capacity for non-invasive ventilation in children with severe pneumonia, or readily available cardiac evaluation resources for children with hypoxemia alone. Duke et al showed improved case fatality rates by providing oxygen concentrators and pulse-oximeters at five hospitals in PNG along with protocols for use [35]. Lack of quality care at referral hospitals in developing countries is a recognized barrier to health care seeking behavior[40, 41].

If our study reveals pulse-oximetry has influenced health-seeking behavior significantly, we will need to follow with a more systematic evaluation of pneumonia care at rural hospitals which vary in quality of care. We will also need to correlate recovery rates in those who accepted hospital referral versus those who stayed home on oral amoxicillin. Evidence to support home care for severe pneumonia is poor [42].

For peer review only - http://bmjopen.bmj.com/site/about/guidelines.xhtml

The COVID pandemic has adversely affected recruitment in the study with disruption of field activities for many months in 2020. We anticipate prolongation of study duration in order to achieve sample size.

Ethics and Dissemination

This study has been approved by the Ethical Review Committee of The Aga Khan University (4722-Ped-ERC-17), Karachi, Pakistan in June 2017. Written informed consent in the local language will be obtained from parents or guardians of all participants. Data forms will contain no identifying information other than age, sex and GIS coordinates. Laboratory forms will contain no identifying information, specimens will be identified by a study number only and test results will not be linked to any individual by name. All survey staff will sign a confidentiality agreement to ensure that they do not release participant identities and test or study results to individuals who are not part of the study team.

Study progress and findings will be shared with sponsors (BMGF) quarterly. Results will be presented at national and international research meetings and conferences and also prepared for publication in international peer-reviewed scientific journals. Study findings will be disseminated to the study communities.

Declarations:

Consent for publication: Yes

Availability of data and materials: The datasets used for the article and the study is available from the corresponding author on request.

Competing interests: The authors declare that they have no competing interests.

Funding: The study is funded by Bill & Melinda Gates Foundation through Grant OPP1148892.

Author Contributions: FM, ZM, MAH, SBS & ZAB: conceptualization of project. FM, AAN, and SC: development of study design and questionnaires. AAN and SC: oversight of data collection. AAN, SC and AH: support of study logistics and field activities. IA and AR: statistical analyses. SBS and ZAB: overall supervision and critical input. All authors have read and approved the final manuscript.

For peer review only - http://bmjopen.bmj.com/site/about/guidelines.xhtml

Acknowledgements: We would like to acknowledge the mothers and families who have contributed to the study. We are grateful to the Sindh LHW Program, Department of Health for their support and facilitation of the trial.

For peer review only

For peer review only - http://bmjopen.bmj.com/site/about/guidelines.xhtml

Figure caption

Figure 1: Trial Profile

Figure 2: Study Map: intervention and control sites

References

1. Liu, L., et al., *Global, regional, and national causes of child mortality: an updated systematic analysis for 2010 with time trends since 2000.* Lancet, 2012. **379**(9832): p. 2151-61.
2. Rudan, I., et al., *Epidemiology and etiology of childhood pneumonia.* Bull World Health Organ, 2008. **86**(5): p. 408-16.
3. *WHO/UNICEF Joint Statement: Management of Pneumonia in community settings.* 2002.
4. *Ending Preventable Child Deaths from Pneumonia and Diarrhoea by 2025: The integrated Global Action Plan for Pneumonia and Diarrhoea (GAPPD)*. 2013, World Health Organization: Geneva, Switzerland.
5. Soofi, S., et al., *Effectiveness of community case management of severe pneumonia with oral amoxicillin in children aged 2-59 months in Matiari district, rural Pakistan: a cluster-randomised controlled trial.* Lancet, 2012. **379**(9817): p. 729-37.
6. Kallander, K., D.H. Burgess, and S.A. Qazi, *Early identification and treatment of pneumonia: a call to action.* Lancet Glob Health, 2015. **4**(1): p. e12-3.
7. Owais, A., et al., *Why do families of sick newborns accept hospital care? A community-based cohort study in Karachi, Pakistan.* J Perinatol, 2011. **31**(9): p. 586-92.
8. Zaidi, A.K., et al., *Community-based treatment of serious bacterial infections in newborns and young infants: a randomized controlled trial assessing three antibiotic regimens.* Pediatr Infect Dis J, 2012. **31**(7): p. 667-72.
9. Ferdous, F., et al., *Mothers' perception and healthcare seeking behavior of pneumonia children in rural bangladesh.* ISRN Family Med, 2014: p. 690315.
10. Waiswa, P., et al., *Using the three delays model to understand why newborn babies die in eastern Uganda.* Trop Med Int Health, 2010. **15**(8): p. 964-72.
11. *LADY HEALTH WORKER PROGRAM FOR FAMILY PLANNING AND PRIMARY HEALTH CARE*. Available from: https://www.sindhhealth.gov.pk/LHW-Program.
12. Afsar, H., et al., *Factors affecting unsuccessful referral by the lady health workers in Karachi, Pakistan.* Journal-Pakistan Medical Association, 2003. **53**(11): p. 521-528.
13. Onyango, F.E., et al., *Hypoxaemia in young Kenyan children with acute lower respiratory infection.* BMJ, 1993. **306**(6878): p. 612-5.
14. Smyth, A., H. Carty, and C.A. Hart, *Clinical predictors of hypoxaemia in children with pneumonia.* Ann Trop Paediatr, 1998. **18**(1): p. 31-40.
15. Usen, S., et al., *Clinical predictors of hypoxaemia in Gambian children with acute lower respiratory tract infection: prospective cohort study.* BMJ, 1999. **318**(7176): p. 86-91.
16. McCollum, E.D., et al., *Multicenter study of hypoxemia prevalence and quality of oxygen treatment for hospitalized Malawian children.* Trans R Soc Trop Med Hyg, 2013. **107**(5): p. 285-92.
17. Basnet, S., R.K. Adhikari, and C.K. Gurung, *Hypoxemia in children with pneumonia and its clinical predictors.* Indian J Pediatr, 2006. **73**(9): p. 777-81.
18. Fu, L.Y., et al., *Brief hospitalization and pulse oximetry for predicting amoxicillin treatment failure in children with severe pneumonia.* Pediatrics, 2006. **118**(6): p. e1822-30.

For peer review only - http://bmjopen.bmj.com/site/about/guidelines.xhtml

19. Duke, T., et al., *Hypoxaemia in acute respiratory and non-respiratory illnesses in neonates and children in a developing country.* Arch Dis Child, 2002. **86**(2): p. 108-12.

20. Weber, M.W., et al., *Predictors of hypoxaemia in hospital admissions with acute lower respiratory tract infection in a developing country.* Arch Dis Child, 1997. **76**(4): p. 310-4.

21. Floyd, J., et al., *Evaluating the impact of pulse oximetry on childhood pneumonia mortality in resource-poor settings.* Nature, 2015. **528**(7580): p. S53-9.

22. Emdin, C.A., et al., *Utility and feasibility of integrating pulse oximetry into the routine assessment of young infants at primary care clinics in Karachi, Pakistan: a cross-sectional study.* BMC Pediatr, 2015. **15**: p. 141.

23. Funk, L.M., et al., *Global operating theatre distribution and pulse oximetry supply: an estimation from reported data.* The Lancet, 2010. **376**(9746): p. 1055-1061.

24. Ingram, G. and N. Munro, *The use (or otherwise) of pulse oximetry in general practice*. 2005, British Journal of General Practice.

25. Mokuolu, O.A. and O.A. Ajayi, *Use of an oxygen concentrator in a Nigerian neonatal unit: economic implications and reliability.* Ann Trop Paediatr, 2002. **22**(3): p. 209-12.

26. Matai, S., et al., *Implementing an oxygen programme in hospitals in Papua New Guinea.* Ann Trop Paediatr, 2008. **28**(1): p. 71-8.

27. Dobson, M., D. Peel, and N. Khallaf, *Field trial of oxygen concentrators in upper Egypt.* Lancet, 1996. **347**(9015): p. 1597-9.

28. Enarson, P., et al., *Implementation of an oxygen concentrator system in district hospital paediatric wards throughout Malawi.* Bull World Health Organ, 2008. **86**(5): p. 344-8.

29. Hill, S.E., et al., *Oxygen for treatment of severe pneumonia in The Gambia, West Africa: a situational analysis.* Int J Tuberc Lung Dis, 2009. **13**(5): p. 587-93.

30. Howie, S.R., et al., *Meeting oxygen needs in Africa: an options analysis from the Gambia.* Bull World Health Organ, 2009. **87**(10): p. 763-71.

31. Pakistan, P.B.o.S.G.o. *PROVINCE WISE PROVISIONAL RESULTS OF CENSUS - 2017*. 2017; Available from: http://www.pbs.gov.pk/content/block-wise-provisional-summary-results-6th-population-housing-census-2017-january-03-2018.

32. Zhu, N., et al., *Lady health workers in Pakistan: improving access to health care for rural women and families.* Boston: Harvard School of Public Health, 2014.

33. Organization, W.H., *Global health workforce alliance. Pakistan's lady health worker Programme: Global Health workforce alliance.* World Health Organization, Case study. Geneva: World Health Organization, 2008.

34. *Integrated Management of Childhood Illness (IMCI) (revised)*. 2014, Geneva: World Health Organization/The United Nation Children's Fund (UNICEF).

35. Duke, T., et al., *Improved oxygen systems for childhood pneumonia: a multihospital effectiveness study in Papua New Guinea.* The Lancet, 2008. **372**(9646): p. 1328-1333.

36. Ferdous, F., et al., *Pneumonia mortality and healthcare utilization in young children in rural Bangladesh: a prospective verbal autopsy study.* Tropical medicine and health, 2018. **46**(1): p. 1-10.

37. Engel, M.S. and L.K. Kochilas, *Pulse oximetry screening: a review of diagnosing critical congenital heart disease in newborns.* Medical Devices (Auckland, NZ), 2016. **9**: p. 199.

38. Tekleab, A.M. and Y.C. Sewnet, *Role of pulse oximetry in detecting critical congenital heart disease among newborns delivered at a high altitude setting in Ethiopia.* Pediatric Health, Medicine and Therapeutics, 2019. **10**: p. 83.

39. Jawin, V., et al., *Beyond critical congenital heart disease: newborn screening using pulse oximetry for neonatal sepsis and respiratory diseases in a middle-income country.* PLoS One, 2015. **10**(9): p. e0137580.

For peer review only - http://bmjopen.bmj.com/site/about/guidelines.xhtml

516 40. Latunji, O. and O. Akinyemi, *Factors influencing health-seeking behaviour among civil servants in Ibadan, Nigeria.* Annals of Ibadan postgraduate medicine, 2018. **16**(1): p. 52-60.

518 41. Musoke, D., et al., *Health seeking behaviour and challenges in utilising health facilities in Wakiso district, Uganda.* African health sciences, 2014. **14**(4): p. 1046-1055.

520 42. Das, R.R. and M. Singh, *Treatment of severe community-acquired pneumonia with oral amoxicillin in under-five children in developing country: a systematic review.* PloS one, 2013. **8**(6): p. e66232.

522

523

For peer review only

For peer review only - http://bmjopen.bmj.com/site/about/guidelines.xhtml

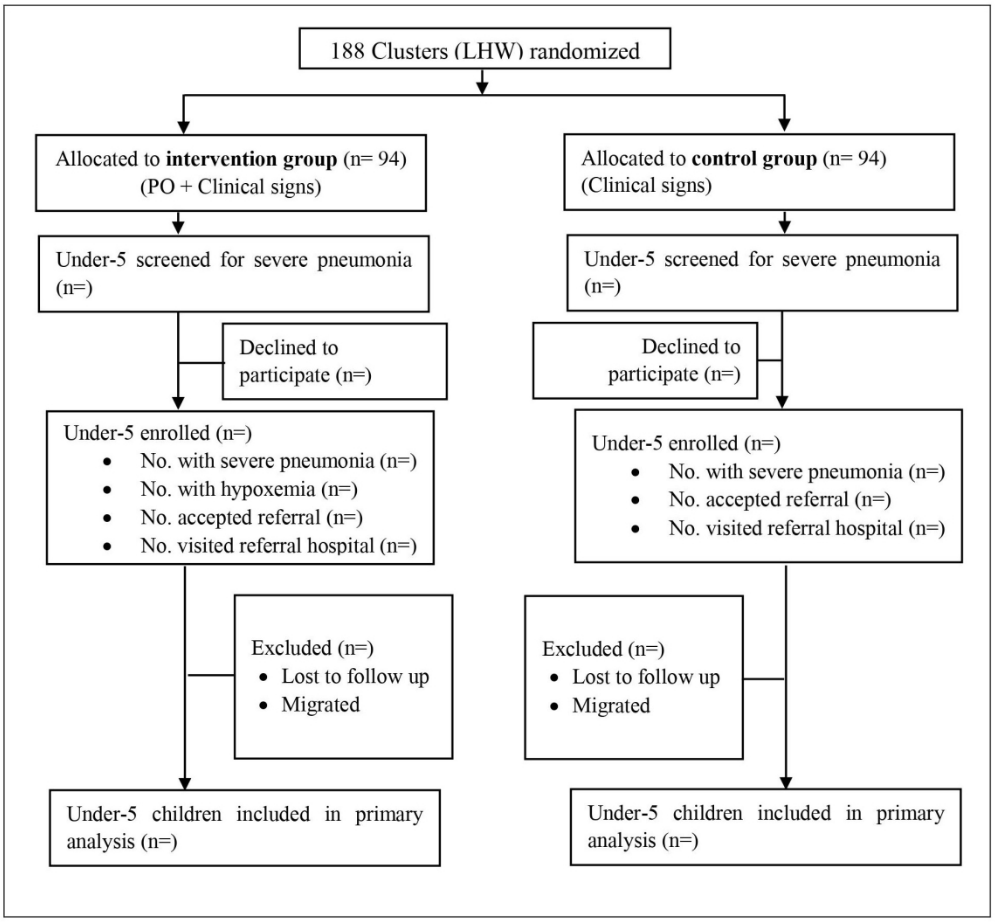

Trial Profile

514x481mm (96 x 96 DPI)

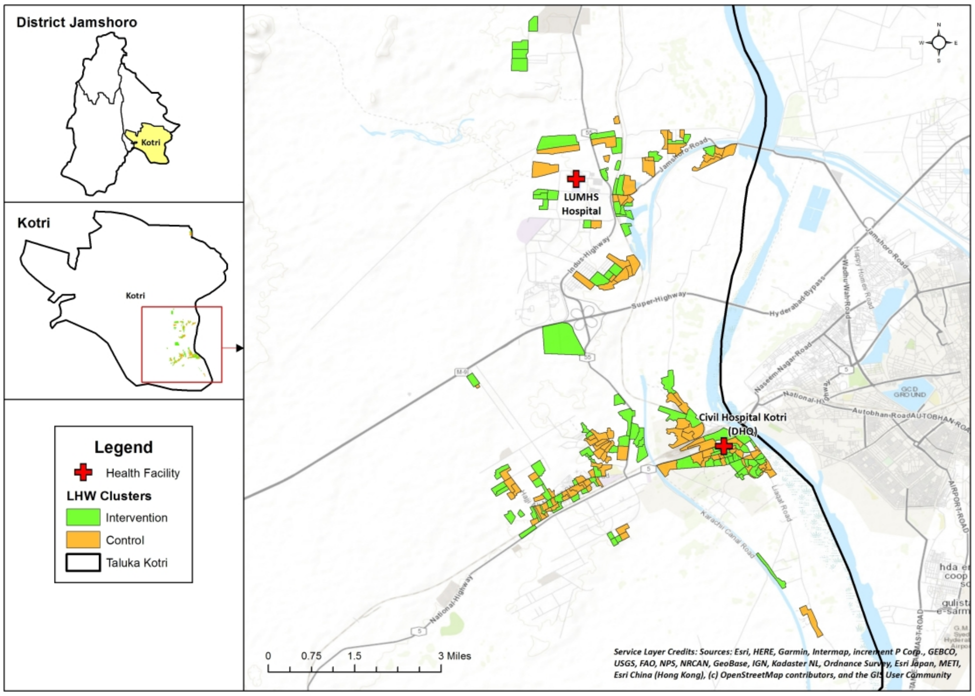

Study Intervention and Control Sites

296x210mm (300 x 300 DPI)

Appendix 1: Eligibility/Hospital Referral Criteria

**Hospital Referral Criteria**	
**Intervention Group**	**Control Group**
**Severe pneumonia AND/OR Hypoxemia**<br><br>**Children of 0 – 6 days:**<br>**1.** 1. Fast Breathing (≥ 60 breaths/min)<br>**AND/OR**<br>***2. Hypoxemia (<92 SpO2 Blood Oxygen Level)***<br><br>**Children of 7 days – 59 months:**<br>**1.** Fast Breathing and/or Chest In-drawing<br>➢ 0-2months: ≥ 60 breaths/min<br>➢ 2-12months: ≥50 breaths/min<br>➢ 12-59months ≥40 breaths/min<br>**AND**<br>**2.** Any ONE General Danger Sign* and/or Stridor<br>➢ *Unable to drink/eat<br>➢ Vomiting<br>➢ Convulsions<br>➢ Lethargy/Unconsciousness<br><br>**AND/OR**<br><br>**Hypoxemia (<92 SpO2 Blood Oxygen Level)**	**Severe pneumonia**<br><br>**Children of 0 – 6 days:**<br>**1.** Fast Breathing (≥ 60 breaths/min)<br><br>**Children of 7 days – 59 months:**<br>**1.** Fast Breathing and/or Chest In-drawing<br>➢ 0-2months: > 60 breaths/min<br>➢ 2-12months: ≥50 breaths/min<br>➢ 12-59months ≥40 breaths/min<br>AND<br>**2.** Any ONE General Danger Sign* and/or Stridor<br>➢ *Unable to drink/eat<br>➢ Vomiting<br>➢ Convulsions<br>➢ Lethargy/Unconsciousness

For peer review only

Appendix 2: Informed Consent

Research Consent Form

Title of Research Project:

Impact of Pulse Oximetry on Hospital Referral Acceptance in children under 5 with severe pneumonia in rural Pakistan (District Jamshoro): a cluster randomized trial (GAPPD Scale up Project)

Investigators:

Dr. Fatima Mir (Principal investigator PO Project, AKU)

Department of Pediatrics and Child Health, Aga Khan University, Karachi, Pakistan

Tel: 92-21-34864955

Dr. Sajid Soofi (PI, Scale up of GAPPD in Pakistan)

Centre of Excellence in Women & Child Health, The Aga Khan University, Pakistan.

Tel: 92-21-34864955

Professor Zulfiqar Bhutta (Senior Investigator, Scale up of GAPPD in Pakistan)

Centre of Excellence in Women & Child Health, The Aga Khan University, Pakistan.

Tel: 92-21-34864955

Purpose of the Research:

Severe Pneumonia is a serious and possibly life-threatening infection in young children aged under 5 years. It may involve fast breathing, chest indrawing, and danger signs like inability to feed, convulsions, persistent vomiting and decreased movement. Hypoxemia is now recognized as an additional sign of severe pneumonia needing hospital admission. Pulse oximetry (the 'oxygen test') is a simple method whereby lady health workers can check level of oxygen in blood of children and decide which young babies need immediate medical care and referral to hospital for antibiotics and oxygen and which babies can be treated in the health center itself.

In this study, we are testing whether use of pulse oximetry in addition to other danger signs of severe pneumonia will influence families to accept hospital referral in comparison to areas where danger signs other than hypoxemia are used.

This project is a collaboration between Aga Khan University (AKU) in Karachi and the Gates Foundation, Seattle, USA.

For peer review only

Description of the Research:

We are inviting children aged 0 to 59 months screened at home to join this research study. If your baby joins the study, the following activities will take place:

1) **Questionnaire**. We will ask several questions about the health of your baby, as well as check him/her for presence of pneumonia. If he/she has signs of severe pneumonia (with or without hypoxemia), he will be eligible to take part in our study.

2) **Pulse oximetry**. Next your child may or may not undergo a pulse oximetry measurement dependent on whether he resides in an intervention or non-intervention cluster. The process will be painless for the child and will involve placing a sensor on your baby's foot or hand. Each sensor is attached to a pulse oximetry machine. A number on the screen of the machine shows the oxygen level. A level including and above 92% will be regarded as normal.

   All babies with fast breathing or chest in drawing pneumonia AND any one of general danger signs (inability to feed, persistent vomiting, decreased movement, convulsions) with or without hypoxemia (SaO2 <92%) will be advised and helped to go to an assigned hospital for treatment (antibiotics and possibly oxygen supportive therapy). Their clinical status over there will be followed and transfer to hospital and subsequent treatment will be facilitated by the study.

   All responses will be documented on paper forms. We will arrange for quick referral to hospital if your baby has low oxygen level or other signs of serious illness. Follow-up visits by our study staff will be conducted to check the status of your baby.

3) You may be asked detailed questions based on whether you accept or refuse hospital referral to allow us to understand what factors contributed to your decision making. Since this process will take time, we will do it after the child's treatment plan has been started at hospital or at home.

4) **Video recording**. Some infants will be video recorded during the study. The researchers will look at these videos to make quality checks on study conduct and procedures. You can refuse video recording but still join the rest of the study. If you provide permission, some videos may be shown publicly for education purposes, but your child's name will be kept private. The videos or images from them will not be shown or sold for financial profit.

5) **Stored information**. This study is connected to other studies coordinated by the Aga Khan University. If you agree to join this study, we will access information about your baby collected as part of the Aga Khan University demographic surveillance system and other Aga Khan University studies to which you have already allowed, or will allow, your baby to join.

We expect to enrol about 4160 children with severe pneumonia in this study.

Potential Harms, Discomforts, or Inconveniences:

There are no harms or discomforts that could be caused to your baby by taking part in this study.

Potential Benefits to individual participants:

Your baby will receive free treatment at the hospital if referral is accepted. The baby will also receive two follow up visits to check if he/she is recovering as expected on day 7 and 14 as part of the study. If you refuse to accept hospital referral for injectable antibiotics and oxygen, we will provide less ideal oral treatment options at home.

Potential Benefits to Society:

We may learn more about the usefulness of pulse oximetry for assessment of young babies in your community and other similar places.

Confidentiality:

We will respect your privacy. No information about you or your child will be given to anyone or be published without your permission, unless required by law. The paper forms, videos and electronic information made in this study will be stored in a secure, locked location. Only members of the research team will have access to them. BMGF (the sponsor) or AKU Clinical Research Monitors may look at your child's records to check on the study. By signing this consent form, you agree to let these people look at your child's records. We will put a copy of this research consent form in your child's patient health record and give you a copy as well. After the study has been completed, the forms and videos will be kept as long as required by BMGF and AKU policies. They will then be destroyed according to these same policies. Paper and electronic forms from this study will be stored for at least 7 years after publication of the study. Published study results will not reveal your identity or the identity of your baby.

Payment

No payment will be given for participation in this study.

Participation:

You decide if your child joins this study. If you choose to let your child take part, you can take your child out of the study at any time.

In the unlikely situation that your child becomes ill or is harmed because of study participation, we will treat your child for free. Your signing this consent form does not interfere with your legal rights in any way. The staff of the study, any people who gave money for the study, or the hospital are still responsible, legally and professionally, for what they do during this study.

Sponsorship:

The funder of this research is the Bill and Melinda Gates Foundation Seatttle, USA.

Conflicts of interest:

None of the researchers have any conflicts of interest to declare.

Consent:

By signing this form, you agree that:

1) The study has been explained to you.
2) All of your questions have been answered.
3) The possible harms and benefits of this study have been explained to you.
4) You may ask questions about the study now and in the future.
5) You have been told that your child's medical records will be kept private except as described to you.
6) You understand that information about your child will not be given to anyone or be published without first asking your permission.

I agree that my child____ may take part in this study.

I understand that videos may be used to assess the success of pulse oximetry. I understand that I may refuse to permit video-recording of my child, yet take part in other aspects of the study. I understand that even if I permit video-recording now, I may refuse the use of these videos at a later time. I understand that I may permit video-recording of my child for researchers to view, but refuse any public display of the videos.

For peer review only

I agree to permit my child and me to be video-recorded during this study.

☐ YES ☐ NO

I agree to permit whole or parts of videos of my child and me to be shown publicly for educational purposes.

☐ YES ☐ NO

______________________________ ______________________________

Printed Name of Parent/Legal Guardian | Parent/Legal Guardian's signature/Thumb print & date

______________________________ ______________________________

Printed Name of person who explained consent | Signature of Person who explained consent & date

______________________________ ______________________________

Printed Witness' name | Witness' signature & date

(If the parent/legal guardian does not read Sindhi)

If you have any questions about this study, please call Dr Fatima Mir at __________________

If you have questions about your child's rights as a subject in a study or injuries during a study, please call Coordinator, Bioethics Unit at ___________________

For peer review only

Appendix 3: Study Activities

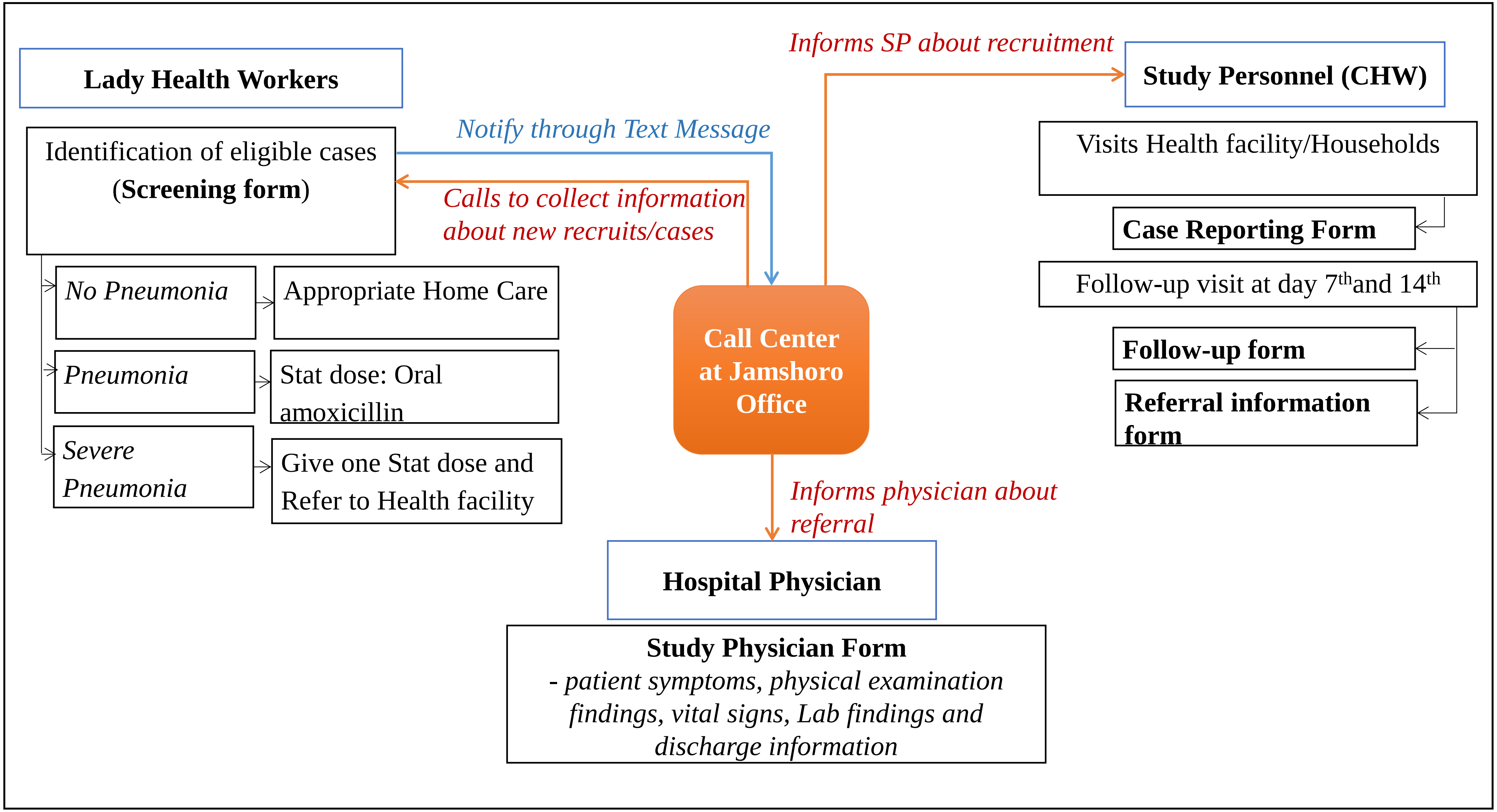

For peer review only

SPIRIT 2013 Checklist: Recommended items to address in a clinical trial protocol and related documents*

Section/item	Item No	Description	Page numbers:
**Administrative information**			
Title	1	Descriptive title identifying the study design, population, interventions, and, if applicable, trial acronym	Title page
Trial registration	2a	Trial identifier and registry name. If not yet registered, name of intended registry	Page 1
	2b	All items from the World Health Organization Trial Registration Data Set	Page 1
Protocol version	3	Date and version identifier	
Funding	4	Sources and types of financial, material, and other support	Page 14
Roles and responsibilities	5a	Names, affiliations, and roles of protocol contributors	Title page and page 14
	5b	Name and contact information for the trial sponsor	
	5c	Role of study sponsor and funders, if any, in study design; collection, management, analysis, and interpretation of data; writing of the report; and the decision to submit the report for publication, including whether they will have ultimate authority over any of these activities	
	5d	Composition, roles, and responsibilities of the coordinating centre, steering committee, endpoint adjudication committee, data management team, and other individuals or groups overseeing the trial, if applicable (see Item 21a for data monitoring committee)	
**Introduction**			

For peer review only

Background and rationale	6a	Description of research question and justification for undertaking the trial, including summary of relevant studies (published and unpublished) examining benefits and harms for each intervention	Pages 3 - 5
	6b	Explanation for choice of comparators	Page 4
Objectives	7	Specific objectives or hypotheses	Page 5
Trial design	8	Description of trial design including type of trial (eg, parallel group, crossover, factorial, single group), allocation ratio, and framework (eg, superiority, equivalence, noninferiority, exploratory)	Page 5
**Methods: Participants, interventions, and outcomes**			
Study setting	9	Description of study settings (eg, community clinic, academic hospital) and list of countries where data will be collected. Reference to where list of study sites can be obtained	Page 6
Eligibility criteria	10	Inclusion and exclusion criteria for participants. If applicable, eligibility criteria for study centres and individuals who will perform the interventions (eg, surgeons, psychotherapists)	Page 6
Interventions	11a	Interventions for each group with sufficient detail to allow replication, including how and when they will be administered	Pages 7 - 9
	11b	Criteria for discontinuing or modifying allocated interventions for a given trial participant (eg, drug dose change in response to harms, participant request, or improving/worsening disease)	Not applicable
	11c	Strategies to improve adherence to intervention protocols, and any procedures for monitoring adherence (eg, drug tablet return, laboratory tests)	Not applicable
	11d	Relevant concomitant care and interventions that are permitted or prohibited during the trial	

For peer review only - http://bmjopen.bmj.com/site/about/guidelines.xhtml

Outcomes	12	Primary, secondary, and other outcomes, including the specific measurement variable (eg, systolic blood pressure), analysis metric (eg, change from baseline, final value, time to event), method of aggregation (eg, median, proportion), and time point for each outcome. Explanation of the clinical relevance of chosen efficacy and harm outcomes is strongly recommended	Pages 5-6
Participant timeline	13	Time schedule of enrolment, interventions (including any run-ins and washouts), assessments, and visits for participants. A schematic diagram is highly recommended (see Figure)	Page 6
Sample size	14	Estimated number of participants needed to achieve study objectives and how it was determined, including clinical and statistical assumptions supporting any sample size calculations	Page 12
Recruitment	15	Strategies for achieving adequate participant enrolment to reach target sample size	Pages 7 - 9

Methods: Assignment of interventions (for controlled trials)

Allocation:

Sequence generation	16a	Method of generating the allocation sequence (eg, computer-generated random numbers), and list of any factors for stratification. To reduce predictability of a random sequence, details of any planned restriction (eg, blocking) should be provided in a separate document that is unavailable to those who enrol participants or assign interventions	Pages 6 - 7
Allocation concealment mechanism	16b	Mechanism of implementing the allocation sequence (eg, central telephone; sequentially numbered, opaque, sealed envelopes), describing any steps to conceal the sequence until interventions are assigned	Pages 6 - 7
Implementation	16c	Who will generate the allocation sequence, who will enrol participants, and who will assign participants to interventions	Pages 6 - 8
Blinding (masking)	17a	Who will be blinded after assignment to interventions (eg, trial participants, care providers, outcome assessors, data analysts), and how	

For peer review only - http://bmjopen.bmj.com/site/about/guidelines.xhtml

	17b	If blinded, circumstances under which unblinding is permissible, and procedure for revealing a participant's allocated intervention during the trial	
**Methods: Data collection, management, and analysis**			
Data collection methods	18a	Plans for assessment and collection of outcome, baseline, and other trial data, including any related processes to promote data quality (eg, duplicate measurements, training of assessors) and a description of study instruments (eg, questionnaires, laboratory tests) along with their reliability and validity, if known. Reference to where data collection forms can be found, if not in the protocol	Page 9
	18b	Plans to promote participant retention and complete follow-up, including list of any outcome data to be collected for participants who discontinue or deviate from intervention protocols	Page 12
Data management	19	Plans for data entry, coding, security, and storage, including any related processes to promote data quality (eg, double data entry; range checks for data values). Reference to where details of data management procedures can be found, if not in the protocol	Page 10
Statistical methods	20a	Statistical methods for analysing primary and secondary outcomes. Reference to where other details of the statistical analysis plan can be found, if not in the protocol	Page 12
	20b	Methods for any additional analyses (eg, subgroup and adjusted analyses)	Page 12
	20c	Definition of analysis population relating to protocol non-adherence (eg, as randomised analysis), and any statistical methods to handle missing data (eg, multiple imputation)	
**Methods: Monitoring**			
Data monitoring	21a	Composition of data monitoring committee (DMC); summary of its role and reporting structure; statement of whether it is independent from the sponsor and competing interests; and reference to where further details about its charter can be found, if not in the protocol. Alternatively, an explanation of why a DMC is not needed	Page 10

	21b	Description of any interim analyses and stopping guidelines, including who will have access to these interim results and make the final decision to terminate the trial	
Harms	22	Plans for collecting, assessing, reporting, and managing solicited and spontaneously reported adverse events and other unintended effects of trial interventions or trial conduct	Page 11
Auditing	23	Frequency and procedures for auditing trial conduct, if any, and whether the process will be independent from investigators and the sponsor	
**Ethics and dissemination**			
Research ethics approval	24	Plans for seeking research ethics committee/institutional review board (REC/IRB) approval	Page 14
Protocol amendments	25	Plans for communicating important protocol modifications (eg, changes to eligibility criteria, outcomes, analyses) to relevant parties (eg, investigators, REC/IRBs, trial participants, trial registries, journals, regulators)	Page 10-11
Consent or assent	26a	Who will obtain informed consent or assent from potential trial participants or authorised surrogates, and how (see Item 32)	Page 6
	26b	Additional consent provisions for collection and use of participant data and biological specimens in ancillary studies, if applicable	
Confidentiality	27	How personal information about potential and enrolled participants will be collected, shared, and maintained in order to protect confidentiality before, during, and after the trial	Page 14
Declaration of interests	28	Financial and other competing interests for principal investigators for the overall trial and each study site	Page 14
Access to data	29	Statement of who will have access to the final trial dataset, and disclosure of contractual agreements that limit such access for investigators	Page 14
Ancillary and post-trial care	30	Provisions, if any, for ancillary and post-trial care, and for compensation to those who suffer harm from trial participation	

For peer review only

For peer review only - http://bmjopen.bmj.com/site/about/guidelines.xhtml

Dissemination policy	31a	Plans for investigators and sponsor to communicate trial results to participants, healthcare professionals, the public, and other relevant groups (eg, via publication, reporting in results databases, or other data sharing arrangements), including any publication restrictions	Page 14
	31b	Authorship eligibility guidelines and any intended use of professional writers	Page 14
	31c	Plans, if any, for granting public access to the full protocol, participant-level dataset, and statistical code	
**Appendices**			
Informed consent materials	32	Model consent form and other related documentation given to participants and authorised surrogates	
Biological specimens	33	Plans for collection, laboratory evaluation, and storage of biological specimens for genetic or molecular analysis in the current trial and for future use in ancillary studies, if applicable	

*It is strongly recommended that this checklist be read in conjunction with the SPIRIT 2013 Explanation & Elaboration for important clarification on the items. Amendments to the protocol should be tracked and dated. The SPIRIT checklist is copyrighted by the SPIRIT Group under the Creative Commons "Attribution-NonCommercial-NoDerivs 3.0 Unported" license.

For peer review only

For peer review only - http://bmjopen.bmj.com/site/about/guidelines.xhtml
